# Ribosome stalling is a signal for metabolic regulation by the ribotoxic stress response

**DOI:** 10.1016/j.cmet.2022.10.011

**Published:** 2022-12-06

**Authors:** Goda Snieckute, Aitana Victoria Genzor, Anna Constance Vind, Laura Ryder, Mark Stoneley, Sébastien Chamois, René Dreos, Cathrine Nordgaard, Frederike Sass, Melanie Blasius, Aida Rodríguez López, Sólveig Hlín Brynjólfsdóttir, Kasper Langebjerg Andersen, Anne E. Willis, Lisa B. Frankel, Steen Seier Poulsen, David Gatfield, Zachary Gerhart-Hines, Christoffer Clemmensen, Simon Bekker-Jensen

**Affiliations:** 1 Center for Healthy Aging, Department of Cellular and Molecular Medicine, University of Copenhagen, Blegdamsvej 3B, 2200 Copenhagen, Denmark; 2 MRC Toxicology Unit, University of Cambridge, Tennis Court Road, Cambridge CB2 1QR, UK; 3 Center for Integrative Genomics, University of Lausanne, 1015 Lausanne, Switzerland; 4 Novo Nordisk Foundation Center for Basic Metabolic Research, Faculty of Health and Medical Sciences, University of Copenhagen, Blegdamsvej 3B, 2200 Copenhagen, Denmark; 5 Danish Cancer Research Center, Strandboulevarden 49, 2100 Copenhagen, Denmark; 6 Biotech Research and Innovation Center, University of Copenhagen, Ole Maaløes Vej 5, 2200 Copenhagen, Denmark; 7 Department of Biomedicine, University of Copenhagen, Blegdamsvej 3B, 2200 Copenhagen, Denmark

**Keywords:** amino acid starvation, metabolic regulation, ribosome collision, ribotoxic stress response, mouse models, ZAK-alpha, mTOR, AMPK, FGF21

## Abstract

Impairment of translation can lead to collisions of ribosomes, which constitute an activation platform for several ribosomal stress-surveillance pathways. Among these is the ribotoxic stress response (RSR), where ribosomal sensing by the MAP3K ZAKα leads to activation of p38 and JNK kinases. Despite these insights, the physiological ramifications of ribosomal impairment and downstream RSR signaling remain elusive. Here, we show that stalling of ribosomes is sufficient to activate ZAKα. In response to amino acid deprivation and full nutrient starvation, RSR impacts on the ensuing metabolic responses in cells, nematodes, and mice. The RSR-regulated responses in these model systems include regulation of AMPK and mTOR signaling, survival under starvation conditions, stress hormone production, and regulation of blood sugar control. In addition, ZAK^−/−^ male mice present a lean phenotype. Our work highlights impaired ribosomes as metabolic signals and demonstrates a role for RSR signaling in metabolic regulation.

## Highlights

• ZAKα is activated by nutrient starvation and amino acid deprivation
• Ribotoxic stress signaling intersects with signaling through AMPK and mTOR kinases
• ZAK^−/−^ mice are deficient for hepatic FGF21 induction following leucine starvation
• ZAK^−/−^ mice present with a number of basal metabolic phenotypes

## Graphical abstract

**Figure:**
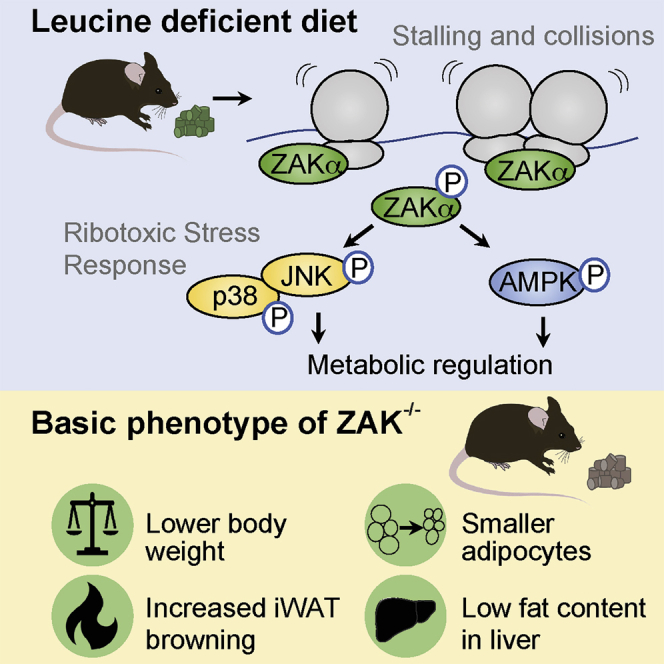

## Teaser

Stalling and collision of ribosomes activate ZAKα and the ribotoxic stress response with poorly understood physiological ramifications. Here, Snieckute et al. show that ZAKα is activated upon amino acid deprivation and intersects with AMPK and mTOR signaling. In addition, ZAK^−/−^ mice display basal as well as leucine starvation-induced metabolic phenotypes.

## Introduction

Organisms experience considerable fluctuations in food availability, necessitating an ability to flexibly store energy when resources are abundant and utilize it when resources are scarce. To achieve energy homeostasis in the face of such challenges, a large degree of metabolic flexibility is required.^1^^,^^2^ This flexibility necessitates fine-tuning by cellular sensors of energy and nutrient availability that mediate switches between anabolism and catabolism. Among these, the mTOR kinase monitors the availability of amino acids and directly impacts protein translation and other anabolic reactions.^3^ Conversely, the AMPK kinase responds to low energy levels by increasing glucose uptake and lipid oxidation.^4^ In mammalian organisms, the liver is the main site of glucose storage and release, while adipose tissues store excess energy in the form of lipids. The interplay between these storage sites is particularly evident in the face of energy or nutrient shortage, when mTOR and AMPK signaling mediate release of lipids from adipose tissues, hepatic oxidation of lipids and an increase in hepatic, and peripheral insulin sensitivity.^5^

The ribotoxic stress response (RSR) denotes a pathway that responds to translational aberrations and activates the p38 and JNK kinases^6^ to mediate cell cycle arrest,^7^ production of inflammatory cytokines,^8^ and activate apoptotic signaling.^9^ However, it is not known whether RSR signaling also directly impacts translation or removal of the underlying lesions. Signaling through p38 and JNK kinases impacts multiple aspects of cellular and organismal physiology, not least metabolic regulation.^10^ Thus, p38 isoforms control browning of white adipose tissue (WAT)^11^ and activation of brown adipose tissue (BAT),^12^ while JNK is a negative regulator of insulin sensitivity in multiple tissues,^10^ just to mention a few. The upstream sensor in the RSR pathway is the MAP3 kinase ZAKα, which senses ribotoxic stress by virtue of two C-terminal ribosome binding domains.^8^ At least one activation signal for ZAKα is generated by the collision of ribosomes, and these structures also constitute an activation signal for ZNF598 and GCN2, the upstream activators of ribosome-associated quality control (RQC) and the integrated stress response (ISR), respectively.^6^^,^^9^ Despite these insights, the RSR remains an “orphan pathway” as we do not understand the physiological contexts in which ZAKα-activating translational aberrations occur. Here, we show that starvation and amino acid deprivation induce physiologically relevant translational aberrations and RSR signaling that integrates with core metabolic signaling pathways. In an organismal context, male ZAK^−/−^ mice display several whole-body metabolic phenotypes, including decreased adiposity, adipocyte hypotrophy, and leanness. Upon leucine starvation, ZAK^−/−^ mice respond with over-compensation of blood sugar control and fail to maximally induce hepatic FGF21 production. We conclude that stalled ribosomes constitute an unappreciated signal for metabolic regulation in cells and that the RSR regulates metabolism in whole organisms.

## Results

### The RSR is activated by starvation-induced ribosome stalling

**Figure 1:**
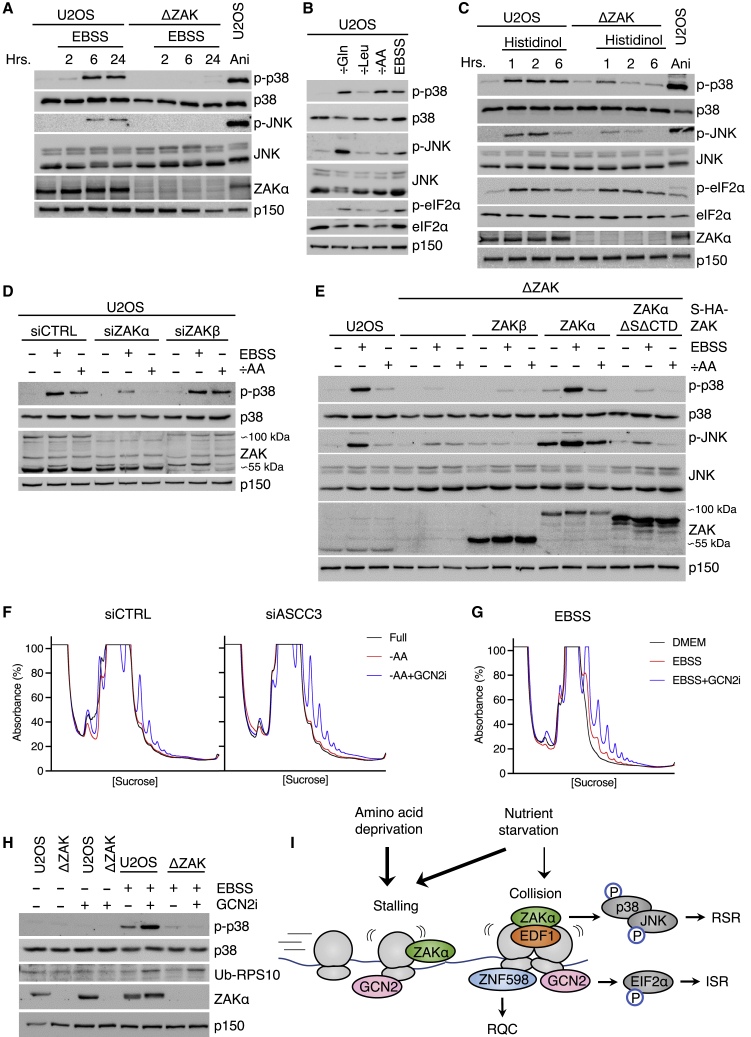
Starvation and amino acid deprivation induce ZAKα-activating ribosome stalls (A) ZAK-dependent phosphorylation of p38 and JNK upon EBSS treatment of U2OS cells. (B) Comparison of different amino acid starvation media (18 h) with respect to phosphorylation of p38, JNK, and eIF2α in U2OS cells. (C) ZAK-dependent phosphorylation of p38, JNK, and eIF2α upon histidinol treatment of U2OS cells. (D) Contribution of ZAK isoforms toward starvation-induced p38 phosphorylation in U2OS cells. (E) Stable rescue of ΔZAK cells with WT and mutated forms of ZAKα and WT ZAKβ. (F) MNase assay to measure ribosome collisions in amino acid starved HeLa cells treated with GCN2 inhibitor (GCN2i) and ASCC3 siRNA as indicated. (G) As in (F), except that cells were incubated in an EBSS medium. (H) Exacerbated ZAK-dependent p38 phosphorylation and appearance of a ribosome collision marker (Ub-RPS10) upon combination of EBSS and GCN2i. (I) Signaling pathways activated at stalled and collided ribosomes induced by starvation and amino acid deprivation. RSR, ribotoxic stress response; RQC, ribosome-associated quality control; ISR, integrated stress response. See also Figures S1 and S2.

Incubation of human U2OS and HeLa cells in an Earle’s balanced salt solution (EBSS) starvation medium for several hours led to a marked activation of p38 and JNK, and these effects were abolished in corresponding ZAK knockout (KO) cells (Figures 1A and S1A). This response appeared to be related to amino acids availability, as incubation with histidinol or medium depleted of either glutamine, leucine or lysine, arginine, and leucine in combination (÷AA) all decreased global translation and triggered RSR signaling (Figures 1B, 1C, and S1A–S1C), as also previously reported for glutamine starvation.^9^ All of the above conditions also activated the ISR, as evidenced by eIF2α phosphorylation (Figures 1B and 1C). While GCN2 appeared to be the most relevant kinase (Figures S1D and S1E), very weak activation of PERK could also be observed (Figure S1F), as previously reported.^13^ Activation of p38 could be clearly attributed to the RSR, as p38 activation in both EBSS and ÷AA medium was abolished upon knockdown of the ribosome-binding ZAKα kinase, but not upon knockdown of its splice variant ZAKβ (Figures 1D and S1G). Furthermore, the phenotype of ZAK KO cells could be rescued by WT ZAKα, but not by WT ZAKβ or a mutant of ZAKα deficient for ribosome binding (ZAKαΔSΔCTD)^8^ (Figures 1E and S1G).

Amino acid starvation has previously been reported to be associated with both stalling and collision of ribosomes.^9^^,^^14^ These structures can be distinguished by *in vitro* digestion of polysomes with micrococcal nuclease (MNase), which leaves the mRNA spanning disomes and trisomes intact.^7^ Using this method, we did not observe any signs of collided ribosomes in cells incubated in ÷AA medium, even in the absence of the disassembly factor ASCC3^15^ (Figures 1F and S1H). However, inhibition of the ISR kinase GCN2, allowing new cap-dependent initiation of translation, was accompanied by ribosome collision, and this effect was exacerbated by ASCC3 knockdown (Figure 1F). The appearance of collision peaks exclusively with the combination of ÷AA medium and GCN2 inhibitor suggests that stalling of ribosomes is sufficient to induce the activation of ZAKα and RSR signaling.

We did, however, observe a mild increase in collision peaks as well as ribosomal recruitment of the collision sensors EDF1,^16^^,^^17^ ubiquitinated RPS10,^18^^,^^19^ and ubiquitinated RPS2^20^^,^^21^ when cells were treated with EBSS (Figures 1G, S1I, S1J, and S2A). The collision-promoting effects of EBSS treatment were clearly exacerbated by GCN2 inhibition (Figure 1G), to an extent where the appearance of mono-ubiquitinated RPS10 could be observed by straight western blotting (Figure 1H). These results indicate that ribosome stalling is also the major translational aberration induced by EBSS. However, conversion of these stalls to collisions was accompanied by elevated ZAK-dependent p38 activation (Figure 1H). We propose that both full starvation and amino acid starvation induce ribosome stalling that activates the RSR. While not an absolute requirement, collided ribosomes appear to provide a more robust platform for ZAKα activation compared with stalled ribosomes (Figure 1I).

### ZAKα regulates AMPK and mTOR activity during metabolic stress responses

**Figure 2:**
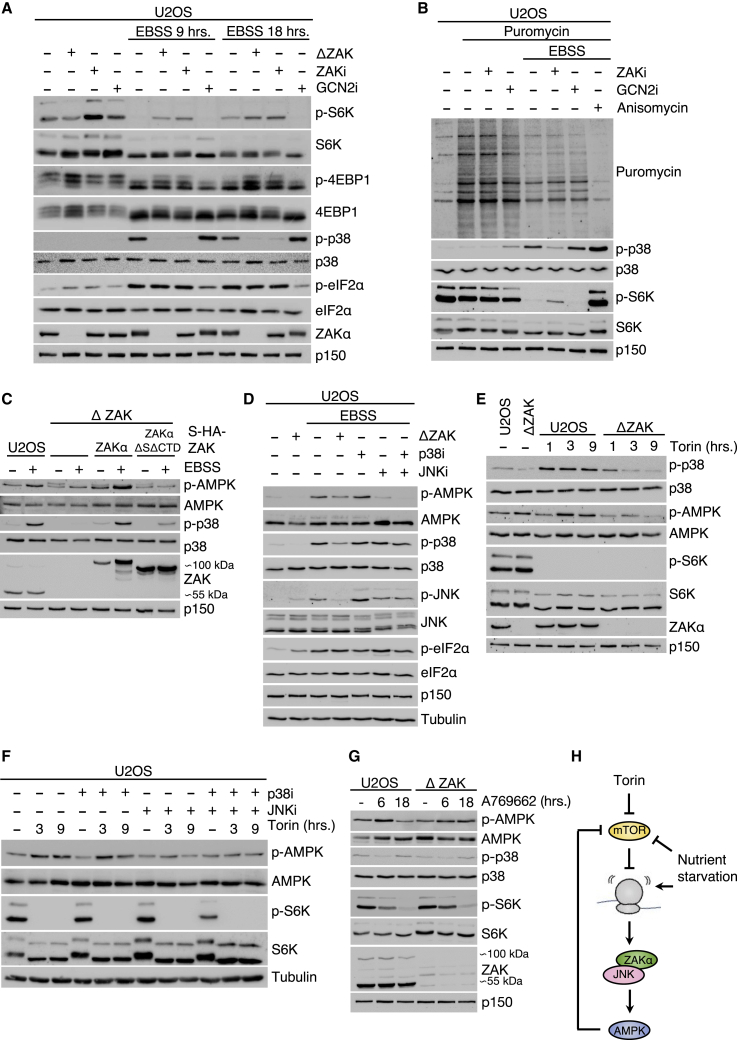
Crosstalk between RSR, AMPK, and mTOR signaling (A) ZAK-dependent regulation of the mTOR targets S6K and 4EBP1 upon EBSS treatment of U2OS cells. (B) Puromycin incorporation assay in EBSS-treated U2OS cells. (C) ZAKα-dependent activation of AMPK in EBSS-treated U2OS cells. (D) JNK-dependent activation of AMPK in EBSS-treated U2OS cells. (E) ZAK-dependent activation of AMPK and p38 in U2OS cells treated with the mTOR inhibitor torin. (F) JNK-dependent activation of AMPK in U2OS cells treated with torin. (G) Lack of ZAKα activation by the AMPK-activating compound A769662. (H) Model of cross-regulation between mTOR, AMPK, and the ribotoxic stress response upon nutrient starvation and catalytic mTOR inhibition (torin), respectively. See also Figure S2.

To investigate a role for the RSR in cellular responses to starvation, we incubated WT and ZAK KO U2OS cells in an EBSS medium and analyzed the phosphorylation status of the mTOR substrates S6 kinase (S6K) and 4EBP1. While robustly downregulated in WT cells, ZAK KO cells or WT cells treated with a ZAK inhibitor retained a pool of mTOR activity (Figures 2A and 2B). Furthermore, in three independent human cell lines (U2OS, TIG3, and HeLa), KO or inhibition of ZAK resulted in a mildly reduced translational downregulation with EBSS and or ÷AA medium, without affecting translation activity in non-perturbed cells (Figures 2B and S2B–S2F). These effects were quantitatively similar to GCN2 inhibition, but we did not observe any dependency of ZAK on eIF2α phosphorylation (Figures 1C and 2A).

EBSS and amino acid starvation-induced activation of the energy-sensing kinase AMPK also required ribosome binding and kinase activity of ZAKα (Figures 2C, S2G, and S2H). Downstream of ZAKα, control over AMPK appeared to be exerted by JNK kinases, as application of a JNK inhibitor, but not p38 inhibitor, phenocopied the effect of ZAK deletion (Figure 2D). Inhibition of mTOR catalytic activity by torin caused spontaneous activation of RSR signaling, and torin-induced AMPK activation was dependent on ZAK and JNK activity (Figures 2E and 2F). Of note, treatment of cells with torin did not result in ribosome collision, even in the presence of GCN2 inhibitor (Figure S2I). Thus, in the context of nutrient starvation, ZAKα exerts negative control on mTOR activity (Figure 2A), while ZAKα itself is activated upon pharmacological inhibition of mTOR (Figure 2E).

Direct activation of AMPK with the chemical compound A769662 also resulted in gradual inactivation of mTOR (Figures 2G and S2J), as previously reported.^22^ This effect was not abolished in ZAK KO cells (Figure 2G), and chemical AMPK activation was not accompanied by RSR activation (Figures 2G and S2J). These observations place the RSR in a signaling pathway leading from mTOR inactivation to AMPK activation, likely via the effects of mTOR on ribosomal translation. Our results implicate the RSR pathway in starvation-induced metabolic regulation and add to the best of our knowledge of the extensive crosstalk between the stress-responsive kinases mTOR and AMPK (Figure 2H).

### *zak-1* nematodes are sensitive to starvation

**Figure 3:**
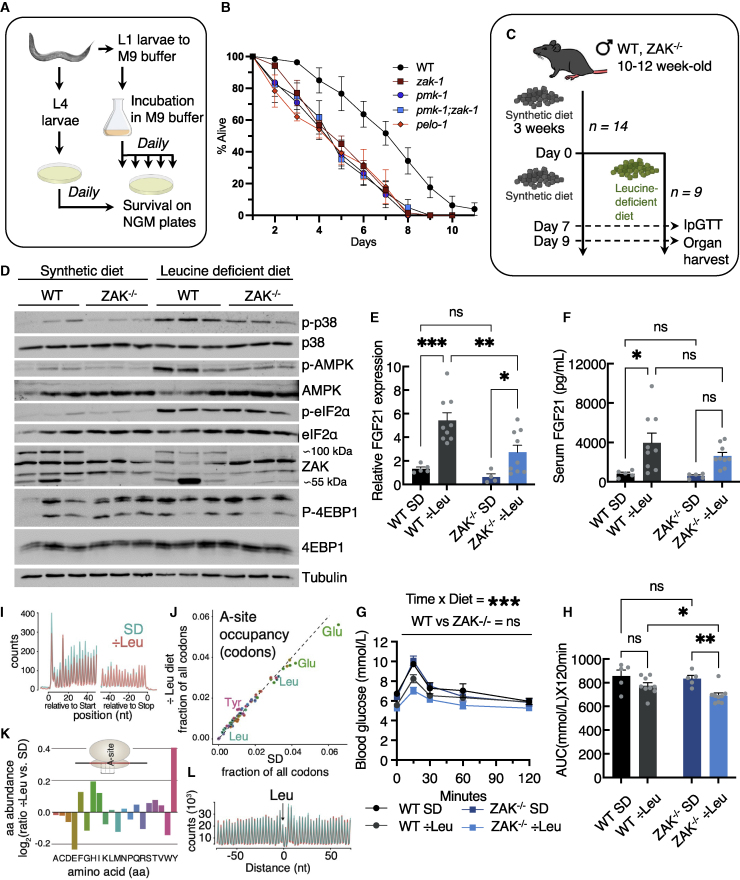
Metabolic regulation by the RSR pathway in model organisms under starvation (A) Schematic of starvation and lifespan experiments with *C. elegans* nematodes. (B) Survival curves for worms with the indicated genotypes in M9 starvation medium (n = 3 biological replicates for all strains). (C) Schematic of mouse leucine starvation experiment. Mice were acclimatized to a full synthetic diet for 3 weeks and randomly assigned to continued full (n = 5 biological replicates) or leucine-deficient (n = 9 biological replicates) synthetic diet. (D) Phosphorylation of p38, eIF2α, AMPK, and mTOR target 4EBP1 in representative livers from (C). (E) qPCR analysis of FGF21 mRNA levels in livers from (C). (F) ELISA detection of circulating serum levels of FGF21 in mice from (C). (G) Blood glucose concentrations of mice from (C) subjected to ipGTT assay. (H) Area under the curve (AUC) calculated for data in (G). All data are plotted as mean and all error bars represent the SEM (G). x, interaction; ns, non-significant; ^∗∗∗^p > 0.001 in three-way ANOVA. (E, F, and H) ns, non-significant; ^∗^p < 0.05; ^∗∗^p < 0.01; ^∗∗∗^p > 0.001 in two-way ANOVA. (I) Metagene analysis of ribosome footprint data, showing the predicted A-site distribution around start and stop codons. (J) Analysis of A-site codon occupancy, comparing datasets from Leu-deficient and SD livers. (K) Analysis of global amino acid occupancy changes, comparing datasets from Leu-deficient and SD livers. (L) Predicted footprint A-site density distributions around leucine (Leu), color-coded as in (I). ÷Leu, leucine-deficient diet; SD, full synthetic diet. See also Figures S3 and S4.

Having established a role for the RSR pathway in regulation of metabolic signaling at the cellular level, we proceeded by studying starvation responses in ZAK-deficient model organisms. Among these, the nematode *C. elegans* is particularly useful, as its genome only encodes the ZAKα isoform. Compared with the WT N2 strain, larvae deficient for the RSR components *zak-1* and *pmk-1* (the main p38 ortholog) survived for shorter time in a starvation medium, and these effects were fully epistatic (Figures 3A and 3B). *pelo-1* mutant worms, defective for the ortholog of human Pelo (hPelota), which is involved in splitting of ribosomes stalled at 3′ ends of mRNAs,^23^ were similarly sensitive to starvation (Figure 3B). Finally, a mutant of the human AMPKα catalytic subunit homolog, *aak-2*, was not further sensitized to starvation upon crossing with *zak-1* worms (Figures S3A and S3B), indicating a similar epistatic relationship between these kinases in *C. elegans* as observed in human cells. These results suggest that nutrient starvation represents a physiologically relevant trigger of ribosomal impairment in multicellular organisms and that the RSR pathway is required for adaptation at an organismal scale under such conditions.

### The RSR regulates hepatic FGF21 expression and glucose tolerance in leucine-starved mice

Mice KO for the *Zak* gene develop normally, are fertile, and do not present with obvious macroscopic phenotypes.^24^ We reared 10- to 12-week-old male mice on a fully synthetic diet for 3 weeks, before being randomly assigned to a leucine-deficient synthetic diet (n = 9) or continuation of full diet (n = 5) for another 9 days (Figure 3C). We noticed that male (but not female) ZAK^−/−^ mice had a slight yet significant reduction in their starting weight compared with their WT littermates, despite an identical food intake (Figures S3C–S3E). Nonetheless, leucine deprivation resulted in a genotype-independent transient reduction in food intake, overall weight loss, and reduction of relative liver mass (Figures S3F–S3H) as previously reported.^25^^,^^26^ Of note, liver is one of the very few tissues with a higher expression of ZAKα compared with ZAKβ (Figures S3I–S3L). Western blotting of liver lysates highlighted starvation-induced activation of p38 and AMPK signaling, both of which were attenuated in ZAK^−/−^ mice (Figures 3D and S4A). The GCN2 target eIF2α was similarly phosphorylated in WT and ZAK^−/−^ livers (Figures 3D and S4A), indicating a lack of dependency of ISR signaling on ZAKα under these conditions.

FGF21 and GDF15 are metabolic stress-induced hormones that regulate insulin sensitivity, thermogenesis, and food intake.^27^ They are strongly induced in the liver upon leucine starvation, and this reaction has been attributed to ISR signaling.^28^^,^^29^ FGF21 and GDF15 induction appeared reduced in ZAK^−/−^ livers (qPCR) and blood (ELISA); however, these effects only reached statistical significance for *Fgf21* mRNA levels (Figures 3E, 3F, S4B, and S4C). Leucine starvation is also known to improve blood glucose tolerance in male mice,^30^ and this response was even stronger in ZAK^−/−^ mice, compared with their WT littermates (Figures 3G and 3H) with no differences observed for HOMA-IR, blood glucose and insulin levels (Figures S4D–S4F). Our results indicate a role for RSR signaling in both liver-specific and whole-body metabolic adaptation to amino acid starvation in mice.

### Leucine starvation activates RSR signaling in the absence of ribosomal collisions or a generalized ribosomal decoding defect in mouse liver

We next carried out ribosome profiling (ribo-seq) on snap-frozen WT livers from Figure 3C according to established protocols.^31^ Our analysis indicated lower footprint coverage at the 5′ end of the coding sequence in leucine-starved mice (Figure 3I), consistent with ISR activation reducing overall translation initiation. To probe for ribosomal decoding defects associated with specific codons or amino acids, we first assessed whether the predicted A-sites of footprints showed diet-dependent changes in their codon distributions. Overall, codons were very similarly occupied in full diet and leucine-starved animals (Figure 3J). Of note, the actually depleted amino acid, leucine, was not affected (Figures 3K and 3L), reminiscent of previous findings from leucine-deprived cell cultures.^14^ Using a recently reported metric to quantify transcriptome-wide pause scores,^32^ we mapped sites with significantly increased (n = 835) and decreased (n = 861) relative ribosome occupancy in leucine-deprived animals (Figure S4G). A metagene alignment of footprints on the set of increased sites (with enriched position anchored at 0 nt) did not reveal any evidence for an increased signal ∼10 codons upstream that would be diagnostic of collided ribosomes (Figure S4H). In summary, these analyses indicate that despite changes to the translational landscape that occur under conditions of leucine deprivation, we could not find any evidence for widespread ribosomal collisions as a candidate for the major ZAKα-activating signal.

### Altered adipose metabolism confers a lean phenotype in ZAK^−/−^ mice

**Figure 4:**
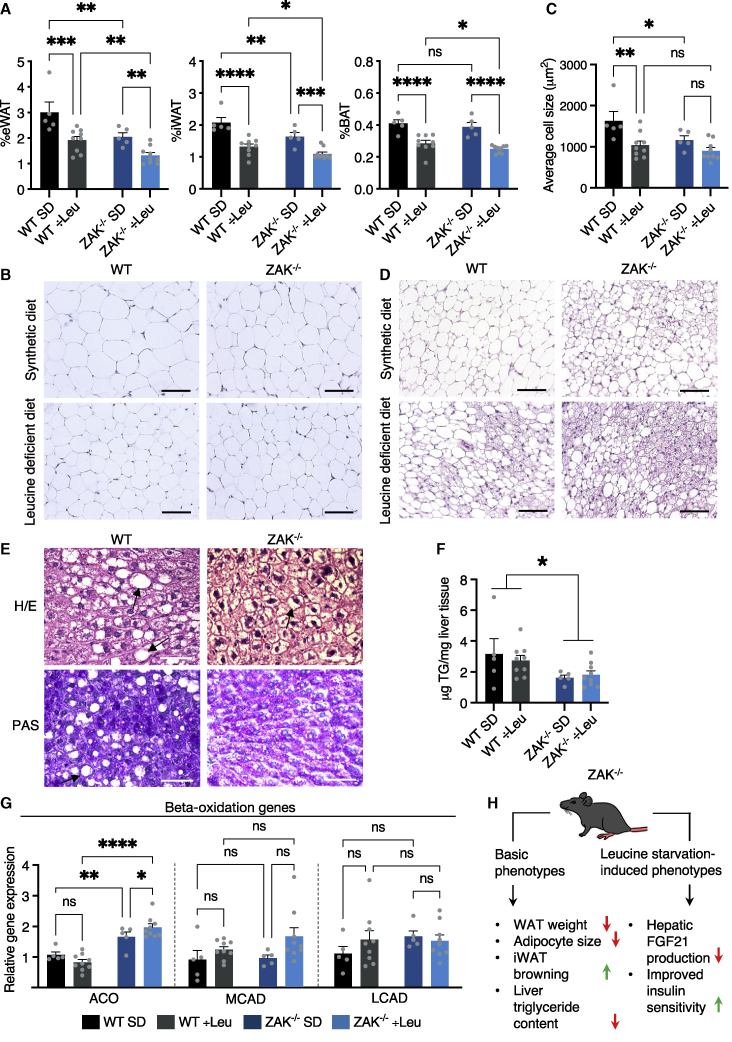
ZAK^−/−^ mice present with altered adipose metabolism and a lean phenotype (A) Percentage weight of eWAT, iWAT, and BAT from mice in Figure 3C. (B) Images of representative hematoxylin and eosin (H&E)-stained WAT. (C) Quantification of adipocyte size in WAT from all mice in (B). (D) Representative images of iWAT browning in mice from Figure 3C (H&E staining). (E) H&E (top) and periodic acid shift (PAS) (bottom) staining of liver sections from mice in Figure 3C. Arrows indicate areas of stochastic mild steatosis. (F) Hepatic triglyceride (TG) levels from mice in Figure 3C. (G) qPCR analysis of mRNA levels of beta-oxidation genes ACO, MCAD, and LCAD in livers from mice in Figure 3C. (H) Model of basic and leucine starvation-induced metabolic phenotypes in ZAK^−/−^ mice. ÷Leu, leucine-deficient diet; SD, full synthetic diet. All scale bars, 50 μm. Data are plotted as mean and all error bars represent the SEM. ns, non-significant; ^∗^p < 0.05; ^∗∗^p < 0.01; ^∗∗∗^p > 0.001, ^∗∗∗∗^p < 0.0001 in two-way ANOVA. See also Figure S4.

In both WT and ZAK^−/−^ mice, leucine starvation caused similar attrition of epididymal and inguinal WAT (eWAT and iWAT) as well as interscapular BAT (Figure 4A). However, independent of the diet, the weights of WAT depots and WAT adipocyte size were overall reduced in ZAK^−/−^ mice (Figures 4A–4C). In addition, iWAT from ZAK^−/−^ mice on full diet was abundant in patches of brown adipocytes (Figure 4D). Hematoxylin and eosin (H&E) and periodic acid shift (PAS) staining of liver sections indicated stochastic mild steatosis and increased hepatic triglyceride (TG) content in some WT mice, which we did not observe in ZAK^−/−^ mice (Figures 4E, 4F, S4I, and S4J). qPCR analysis on liver mRNA showed an upregulation of the gene encoding peroxisomal acyl coenzyme A oxidase (ACO), responsible for lipid degradation by beta-oxidation in ZAK^−/−^ mice (Figure 4G). Enzymes with similar activities (MCAD and LCAD) also appeared to be upregulated in ZAK^−/−^ livers, either under normal or starvation conditions, but these effects did not reach statistical significance (Figure 4G). In contrast, transcription factors and enzymes controlling lipogenesis were similarly regulated before and after leucine starvation (Figure S4K). Our results thus indicate that genetic ablation of RSR signaling is associated with a lean phenotype in mice, characterized by increased lipid turnover and reduced fat deposition.

## Discussion

Our results point to a role for ZAKα-activated JNK in potentiating AMPK activation upon mTOR inactivation. While mTOR itself responds directly to amino acid availability, ZAKα activation appears to rely on the translational shutdown caused by mTOR inactivation when amino acids are scarce. mTOR and AMPK engage in extensive crosstalk,^5^ and augmented AMPK signaling to mTOR is likely to underlie the faster re-appearance of mTOR activity upon prolonged starvation of ZAK KO cells (Figure 2H). Interestingly, while AMPK has been shown to support JNK activation in some biological settings,^33^^,^^34^ we have not been able to find reports on the inverse relationship. The precise mechanism by which ZAKα mediates AMPK activation upon mTOR inhibition thus awaits future investigation.

In mice, RSR signaling contributes to the induction of metabolic stress hormones such as FGF21 in the liver upon leucine starvation (Figures 3E, 3F, and 4H). This response has previously been attributed to GCN2 and the ISR.^28^ The *Fgf21* gene is a target of the transcription factor and ISR effector ATF4,^29^^,^^35^ providing a good rationale for the requirement of ISR signaling. *Fgf21* mRNA, however, is also a highly unstable AU-rich element (ARE) containing transcript that requires the well-known p38-MK2-14-3-3—mediated inactivation of tristetraprolin (TTP) for its stress-induced stabilization.^36^^,^^37^ We propose that these signaling pathways cooperate by inducing stress hormone transcription and mRNA stabilization, respectively. Induction of FGF21 is known to enhance systemic insulin sensitivity.^27^ In spite of the deficiency in inducing this hormone, ZAK^−/−^ mice display increased improvement in blood sugar control compared with WT when starved for leucine (Figures 3G, 3H, and 4H). This mechanism is likely to involve JNK activity, the other arm of the RSR. In the context of starvation, RSR-induced JNK signaling may act as a systemic buffer that limits insulin sensitivity within a biologically relevant range.

GCN2^−/−^ mice have a very different metabolic phenotype, compared with our ZAK^−/−^ strain. When deprived of leucine, these mice do not repress the expression of lipogenic genes and present with massive liver steatosis.^25^ Conversely, ZAK^−/−^ mice displayed upregulation of beta-oxidation genes and decreased hepatic fat content, potentially accounting for the lean phenotype. Another opposing starvation-induced phenotype is the regulation of insulin sensitivity and blood glucose control. While GCN2^−/−^ mice were shown to be defective for this response altogether,^30^ ZAK^−/−^ mice appear to exacerbate the normal improvement in these parameters. In our cell line experiments, and supported by the literature,^7^^,^^14^ we observed that GCN2 inhibition converted starvation-induced ribosomal stalls to ribosomal collisions (Figures 1F and 1G). The latter appears to be the more robust ZAKα-activating signal and was accompanied by substantially stronger RSR signaling (Figure 1H). Given the established links between JNK signaling, insulin sensitivity,^38^ and liver steatosis,^39^ it is interesting to speculate that the reported systemic and liver phenotypes in leucine-starved GCN2^−/−^ mice could be caused by pathological ribosome collision and unchecked RSR signaling.

In summary, our work establishes the RSR pathway as a regulatory node in cellular and organismal metabolism, which functionally interacts with well-established metabolic effectors (e.g., AMPK and mTOR). In the context of starvation and amino acid deprivation of cells, stalled ribosomes, and to a lesser extent, collided ribosomes, appear to constitute the underlying metabolic signal for ZAKα activation.

### Limitations of study

Using MNase-assisted polysome profiling, we did not observe evidence for ribosome collision induced by ZAKα-activating incubation of cells in ÷AA medium. Given the relatively low resolution of this technique (compared with ribosome profiling), we cannot exclude that ribosome collision also occurs under these conditions.

## STAR★Methods

### Key resources table

**Table:** 

REAGENT or RESOURCE	SOURCE	IDENTIFIER
**Antibodies**		

Mouse monoclonal anti-phospho-p38	Cell Signaling	Cat#9216; RRID: AB_331296
Rabbit monoclonal anti-phospho-p38	Cell Signaling	Cat#4511S; RRID: AB_2139682
Mouse polyclonal antibody anti-p38	Cell Signaling	Cat#9212; RRID: AB_330713
Mouse monoclonal anti-phospho-SAPK/JNK	Cell Signaling Technology	Cat#9255; RRID: AB_2307321
Rabbit monoclonal anti-SAPK/JNK	Cell Signaling	Cat#9258; RRID: AB_2141027
Rabbit polyclonal anti-ZAK	Proteintech	Cat#14945-1-AP; RRID: AB_1064269
Rabbit polyclonal anti- ZAKα	Bethyl	Cat#A301-993A; RRID: AB_1576612
Rabbit monoclonal anti-phospho-AMPK	Cell Signaling	Cat#2535, RRID: AB_331250
Rabbit polyclonal anti-AMPK-alpha	Cell Signaling	Cat#2532, RRID: AB_330331
Mouse monoclonal anti-p150	BD biosciences	Cat#610473, RRID: AB_397845
Mouse monoclonal anti-Actin	Millipore	Cat# MAB1501, RRID: AB_2223041
Mouse monoclonal anti-α-Tubulin	Sigma-Aldrich	Cat#T9026, RRID: AB_477593
Mouse monoclonal anti-HA-tag	Santa Cruz Biotechnology	Cat#sc-7392 HRP, RRID: AB_2894930
Rabbit monoclonal anti-phospho-GCN2	Abcam	Cat#ab75837, RRID: AB_1310587
Rabbit monoclonal anti-phospho-eIF2alpha	Cell Signaling	Cat#3398, RRID: AB_2096481
Rabbit polyclonal anti-eIF2alpha	Cell Signaling	Cat#9722, RRID: AB_2230924
Rabbit monoclonal anti-4EBP1	Cell Signaling	Cat#9644, RRID: AB_2097841
Mouse monoclonal anti-Puromycin	Millipore	Cat#MABE343, RRID: AB_2566826
Rabbit monoclonal anti-Ribosomal protein S10	Abcam	Cat#ab151550, RRID: AB_2714147
Rabbit monoclonal anti-phospho-4E-BP1	Cell Signaling	Cat#2855, RRID: AB_560835
Rabbit polyclonal anti-EDF1	Abcam	Cat#ab174651, RRID: AB_2893192
Rabbit polyclonal anti-RPS2	Bethyl	Cat#A303-794A, RRID: AB_11218192
Rabbit monoclonal anti-phospho-PERK	Cell Signaling	Cat#3179, RRID: AB_2095853
Rabbit polyclonal anti-ASCC3	Proteintech	Cat#17627-1-AP, RRID: AB_2059474
Mouse monoclonal anti- RPL19	Novus	Cat#H00006143-M01, RRID: AB_547825
Rabbit monoclonal anti-phospho-p70 S6 kinase	Cell Signaling Technology	Cat#9234, RRID: AB_2269803
Rabbit polyclonal anti-p70 S6 kinase	Cell Signaling Technology	Cat#9202, RRID: AB_331676

**Chemicals, peptides, and recombinant proteins**		

Doxycycline	Sigma-Aldrich	Cat#D3347
Anisomycin	Sigma-Aldrich	Cat#A9789
Puromycin	BioNordika	13884
Histidinol	Sigma-Aldrich	H6647
ZAK inhibitors	Gift from Xiaoyun Lu (Jinan University, China)	N/A
GCN2 inhibitor A-92	Axon medchem	Cat#2720
AMPKact A769662	Tocris	Cat#3336
p38 inhibitor: BIRB 796	Tocris biotechne	Cat#5989
JNK inhibitor: JNK-IN-8	Sigma-Aldrich	Cat#SML1246
PERKi: GSK2606414	SelleckChem	Cat#S7307
FUGENE6	Promega	Cat#E2692
RiboLock RNase Inhibitor	Thermo Fisher Scientific	Cat#EO0381
TRIzol Reagent	Thermo Fisher Scientific	Cat#15596026
NxGen RNase inhibitor	Lucigen	Cat#30281
Micrococcal nuclease	New England Biolabs	Cat#M0247
5′ Deadenylase	NEB	Cat#M0331S
CircLigase II ssDNA Ligase	Lucigen	Cat#CL9025K
COmplete EDTA-free EASYpack	Roche	Cat#4693132001
Corning Costar Spin-X centrifuge tube filters	Sigma-Aldrich	Cat#CLS8162-96EA
DNA Clean & Concentrator-5	Zymo research	Cat#D4014
EpiScript RNase H- Reverse Transcriptase	Lucigen	Cat#ERT12925K
Exonuclease I	Lucigen	Cat#X40520K
Phusion High-Fidelity PCR Master Mix with HF Buffer	NEB	Cat#M0531L
Rec J Exonuclease	Lucigen	Cat#RJ411250
RiboGuard RNase Inhibitor	Lucigen	Cat#RG90910K
RNAse I	Ambion	Cat#AM2295
RNasin Plus RNase Inhibitor	Promega	Cat#N2615
SUPERase⋅In RNase Inhibitor	Invitrogen	Cat#AM2696
SYBR Gold Nucleic Acid Gel Stain	Life Technologies	Cat#S11494
T4 Polynucleotide Kinase, Cloned	Lucigen	Cat#P0503K
T4 RNA Ligase 1	NEB	Cat#M0204L
T4 RNA Ligase 2, Deletion Mutant	Lucigen	Cat#LR2D11310K
Turbo DNAse	Ambion	Cat#AM2239
Torin 1	InvivoGen	Cat#inh-tor1

**Critical commercial assays**		

RNAiMAX	Life Technologies	Cat#13778150
RevertAid RT Reverse Transcription Kit	Thermo Fisher Scientific	Cat#K1691
Power SYBR green	ThermoFisher	Cat#4367659
SensiFAST SYBR green	Bioline	Cat#BIO-98020
QuickExtract DNA Extraction Solution	Lucigen	Cat#QE09050
Click-iT Plus OPP Alxa Fluor 488 protein synthesis assay kit	Thermo Fisher Scientific	Cat#C10456
Infinity Triglycerides Liquid Stable Reagent	Thermo Scientific	Cat#TR22421
*Mouse/Rat FGF-21 Quantikine ELISA Kit*	*R&D systems*	Cat#MF2100
Mouse/Rat GDS-15 Quantikine ELISA Kit	*R&D systems*	Cat#MGD150
Ultra Sensitive Mouse Insulin ELISA	Crystal Chem	Cat#90082

**Deposited data**		

RNA sequencing raw data files	https://www.ncbi.nlm.nih.gov/geo/query/acc.cgi?acc=GSE205191	N/A
Data S1 – Source Data	N/A	N/A

**Experimental models: Cell lines**		

Human osteosarcoma cells (U2OS)	ATCC	HTB-96; RRID: CVCL0042
U2OS Δ ZAK	Vind et al.^6^	N/A
U2OS Δ ZAK+Strep-HA-ZAKα	Vind et al.^6^	N/A
U2OS Δ ZAK+Strep-HA-ZAKα_ΔSΔCTD	Vind et al.^6^	N/A
U2OS Δ ZAK+Strep-HA-ZAKβ	Vind et al.^6^	N/A
human cervical cancer S3 (HeLa S3)	ATCC	CRL-4000; RRID: CVCL 4388
Hela S3 Δ ZAK	This paper	N/A
Mouse embryo fibroblast (NIH/3T3)	ATCC	RRID: CVCL_0594
human diploid embryonic lung fibroblasts (TIG3)	Lisa B. Frankel (Danish Cancer Society)	N/A
Mouse embryonic fibroblasts (MEF’s)	This paper	N/A

**Experimental models: Organisms/strains**		

*C. elegans* Bristol N2	*Caenorhabditis Genetics Center*	N/A
*C. elegans* KU23 *pmk-1(km25) IV*	*Caenorhabditis Genetics Center*	N/A
*C. elegans* RB754 *aak-2(ok524) X*	*Caenorhabditis Genetics Center*	N/A
*C. elegans* PD2860 *pelo-1(cc2849)III;skih-2(cc2854)IV*	*Caenorhabditis Genetics Center*	N/A
*Zak-1 -CRISPR*	Vind et al.^6^	N/A
ZAK knockout mice in the C57BL/6 background	Nordgaard et al.^24^	N/A

**Oligonucleotides**		

Genotyping primers zak-1: Fw1: 5′-CAACGAATGAAAGTCGAGCA-3′, Fw2: 5′-CCGATCAACGTGTGCTCTTT-3′, Rev: 5′-CGCATTCACTCTGTCCAAAA-3′	Vind et al.^6^	N/A
Genotyping primers *aak-2 (ok524*): Fw: 5’-TCATTTGCTGCAACTTCCTG-3’, Rev:5’- GCAGATTTACAATTGGGCG-3’	*Caenorhabditis Genetics Center*	N/A
Genotyping primers *pmk-1(ku25)*: Fw: 5′- CTATAAGTTGCCATGACCTCAG-3’, Rev: 5′- CCACGTATCCAGTCATTTCAG-3’	This paper	N/A
Quantitative RT-PCR FGF21: Fw: 5′- CACCGCAGTCCAGAAAGTCT-3’, Rev: 5′- GCAGGCCTCAGGATCAAAGT-3’	De Sousa-Coelho et al.^35^	N/A
Quantitative RT-PCR GDF15: Fw: 5′- CCGAGAGGACTCGAACTCAG-3’, Rev: 5′- ACCCCAATCTCACCTCTGGA-3’	Patel et al.^29^	N/A
Quantitative RT-PCR ACO: Fw:5’-AAGAGTTCATTCTCAACAGCCC-3’, Rev:5’- CTT GGA CAG ACT CTG AGC TGC-3’	Guo and Cavener^25^	N/A
Quantitative RT-PCR MCAD: Fw:5’-TGG AGA CAT TGC CAATCAGC-3’, Rev:5’ -ACCATAGAGCTGAAGACAGG-3’	Guo and Cavener^25^	N/A
Quantitative RT-PCR SREBP-1c: Fw: 5’-GGCCCGGGAAGTCACTGT-3’, Rev: 5’-GGAGCCATGGATTGCACATT-3’	Guo and Cavener^25^	N/A
Quantitative RT-PCR LCAD: Fw: 5’-AAGGATTTATTAAGGGCAAGAAGC-3’, Rev: 5’-GGAAGCGGAGGCGAGTC-3’	Guo and Cavener^25^	N/A
Quantitative RT-PCR FAS: Fw: 5’-GCTGCGGAAACTTCAGGAAAT-3’, Rev: 5’-AGAGACGTGTCACTCCTGGACTT-3’	Guo and Cavener^25^	N/A
Quantitative RT-PCR ACL: Fw 5’-GCCAGCGGGAGCACATC-3’, Rev 5’-CTTTGCAGGTGCCACTTCATC-3’	Guo and Cavener^25^	N/A
siRNA ZAKα GGTGCCCATTAAGTATCAA	This paper	N/A
siRNA ZAKβ CATGCAAGCCAAGCAGAAT	This paper	N/A
ZAK-sgRNA: Fw: 3′-CACCGTCGAGCCAA ATGGATATCAC-5’, Rev 3′-AAACGT GATATCCATTTGGCTCGAC-5′	Vind et al.^6^	N/A
8NI-810: /5Phos/NNN NNN NNA TCG TAG ATC GGA AGA GCA CAC GTC TGA A/3ddC/	This paper	N/A
8NI-811: /5Phos/NNN NNN NNA GCT AAG ATC GGA AGA GCA CAC GTC TGA A/3ddC/	This paper	N/A
8NI-812: /5Phos/NNN NNN NNC GTA AAG ATC GGA AGA GCA CAC GTC TGA A/3ddC/	This paper	N/A
8NI-813: /5Phos/NNN NNN NNC TAG AAG ATC GGA AGA GCA CAC GTC TGA A/3ddC/	This paper	N/A
8NI-814: /5Phos/NNN NNN NNG ATC AAG ATC GGA AGA GCA CAC GTC TGA A/3ddC/	This paper	N/A
8NI-815: /5Phos/NNN NNN NNG CAT AAG ATC GGA AGA GCA CAC GTC TGA A/3ddC/	This paper	N/A
8NI-816: /5Phos/NNN NNN NNT AGA CAG ATC GGA AGA GCA CAC GTC TGA A/3ddC/	This paper	N/A
8NI-817: /5Phos/NNN NNN NNT CTA GAG ATC GGA AGA GCA CAC GTC TGA A/3ddC/	This paper	N/A
NI802: /5Phos/RNA GAT CGG AAG AGC GTC GTG TAG GGA AAG AG/iSp18/G TGA CTG GAG TTC AGA CGT GTG CTC	McGlincy and Ingolia^40^	N/A
i5: AL3: AATGATACGGCGACCACCG AGATCTACACCCTATCCTacacTC TTTCCCTACACGACGCTC; AL4: AATGATACGGCGACCACCGAGA TCTACACGGCTCTGAacacTCT TTCCCTACACGACGCTC	This paper	N/A
i7: NexteraD503; NexteraD504	Illumina	N/A

**Software and algorithms**		

PRISM 7	GraphPad Software	https://www.graphpad.com/scientific-software/prism/
Fiji	ImageJ2	https://imagej.net/software/fiji/
Cell profiler	CellProfiler	https://cellprofiler.org

**Other**		

Synthetic complete food	Research diets	Custom product based on recipe from Guo and Cavener^25^
Synthetic leucine deficient food	Research diets	Custom product based on recipe from Guo and Cavener^25^
Earle’s Balanced Salts media (EBSS)	Sigma-Aldrich	Cat#E3024
RPMI-1640 medium	Biowest	Cat#L0500
RPMI-1640 medium without L-leucine, L-lysine and L-arginine	Sigma-Aldrich	Cat#R1780
Powdered RPMI media without L-leucine, L-lysine and L-arginine	ThermoFisher Scientific	Cat#88426
RPMI-1640 medium without L-glutamine	Sigma-Aldrich	Cat#R0883

### Resource availability

#### Lead contact

Further information and requests for resources and reagents should be directed to and will be fulfilled by the lead contact, Simon Bekker-Jensen (sbj@sund.ku.dk).

#### Materials availability

Plasmids, cell lines, *C. elegans* strains, and other materials generated in this study are available upon reasonable request to the lead contact.

### Experimental model and subject details

#### Cell lines and primary cells

Female human osteosarcoma cells (U2OS), female human malignant cervical epithelial cells (Hela), male human diploid embryonic lung fibroblasts (TIG3), and mouse embryo fibroblast (NIH/3T3) were cultured in Dulbecco’s Modified Eagle’s Medium (DMEM, Biowest) supplemented with a 10% fetal bovine serum (FBS, Biowest), L-glutamine, penicillin and streptomycin. Primary mouse embryonic fibroblasts (MEF’s) were cultured in DMEM medium supplemented with 15% FBS, L-glutamine, penicillin and streptomycin. All cells were cultured at 37°C in a humidified 5-8% CO_2_ cell incubator.

#### *C. elegans*

*C. elegans* nematodes were maintained as previously described.^41^ In general, all *C. elegans* strains were cultured at 20°C on a standard nematode growth medium (NMG). Plates were seeded with *Escherichia coli* OP50 bacteria as a food source. Strains used in this study were Bristol N2, KU25 *pmk-1(km25) IV, aak-2(ok524), and* PD2860 *pelo-1 (cc2849)III;skih-2(cc2854)IV* that were provided by *Caenorhabditis Genetic Center* (CGC), and *zak-1* deletion strain was generated as previously described.^8^

#### Mice

Mice were housed at the animal facility of the Department of Experimental Medicine at the University of Copenhagen and the research was monitored by the Institutional Animal Care and Use Committee. All the mouse work was performed in compliance with Danish and European regulations. ZAK knockout mouse in the C57BL/6 background was a gift from Vivian S. W. Li (Crick Institute, United Kingdom). C57BL/6 WT and ZAK KO mice were obtained by in-house breeding. Mice were maintained on a 12-h light:dark cycle and were allowed to eat *ad libitum* of commercial rodent chow and water prior to the experiment. At the time of the experiment littermate males were randomly assigned to experimental groups when they were 10-12 weeks of age.

### Method details

#### Cell culture and reagents

Stable cell lines expressing WT or truncated versions of ZAKα and WT ZAKβ under doxycycline inducible promoters were generated as previously described.^8^ To induce starvation cells were washed 3 times in PBS and then cultured in desired media for the time indicated in the figures. To induce full starvation cells were incubated in EBSS media (Sigma, E3024). To induce amino acid deprivation the following medias were used: for L-leucine, L-lysine and L-arginine deprivation (÷AA) RPMI-1640 (Sigma-Aldrich, 1780) or powdered RPMI media (ThermoFisher Scientific, 88426, prepared according to manufacturer’s instructions) was used, for L-Leucine deprivation (÷Leu) RPMI media was supplemented with 48mg/L of L-Lysine (Sigma-Aldrich, L8662) and 200mg/L of L-arginine (Sigma-Aldrich, A8094) and for L-glutamine deprivation (÷Gln) cells were incubated in RPMI-1640 media without L-glutamine (Sigma-Aldrich, R0883). All the RPMI medias were supplemented with 10% of dialysed fetal bovine serum (gift from Jesper V. Olsen, University of Copenhagen, Denmark). The chemicals and inhibitors used in this paper were histidinol (Sigma-Aldrich, H6647, 4 mM), doxycycline (Sigma-Aldrich, D3347, 0.13 μg/mL), anisomycin (Sigma-Aldrich, A9789, 10 μg/ml), puromycin (BioNordika, 13884, 10 μg/mL), torin (InvivoGen, inh-tor1, 0.5 μM), ZAK inhibitor (gift from Xiaoyun Lu, Jinan University, China),^42^ 1 μM), GCN2 inhibitor (Axon medchem, 2720, 1 μM), A769662 (Tocris, 3336, 0.1 mM), p38 inhibitor (Tocris biotechne, 5989, 1 μM), JNK inhibitor (Sigma-Aldrich, SML1246, 1 μM), and PERK inhibitor (SelleckChem, S7307, 1 μM).

#### Western blotting

Cells were lysed in EBC buffer (50 mM Tris, pH 7.5, 150 mM NaCl, 1 mM EDTA, 0.5% NP-40, protease and phosphatase inhibitors). Samples were mixed with Laemmli sample buffer and boiled before they were resolved by SDS-PAGE and transferred to nitrocellulose gel. Membranes were blocked in PBS-T + 5% milk followed by overnight incubation with antibodies at 4°C. Subsequently membranes were washed in PBS-T 5 times and incubated with Goat Anti-Rabbit or Goat Anti-Mouse IgG Antibody (H+L) Peroxidase for 1 h at room temperature. Membranes were washed again in PBS-T and visualized by chemiluminescence (Clarity Western ECL substrate, Bio-Rad) using the Bio-Rad Chemidoc imaging system. Antibodies used in this study: anti-phospho-p38 (Cell Signaling, mouse 9216, rabbit 4511S), anti-p38 (Cell Signaling, 9212, rabbit), anti-phospho-SAPK/JNK (Cell Signaling Technology, 9255, mouse), anti-SAPK/JNK (Cell Signaling, 9258, rabbit), anti-ZAK (Proteintech, 14945-1-AP, rabbit), anti-phospho-AMPK (Cell Signaling, 2535, rabbit), AMPK-alpha (Cell Signaling, 2532, rabbit), anti-p150 (BD biosciences, 610473, mouse), anti-Actin (Millipore, MAB1501, mouse), anti-α-Tubulin (Sigma-Aldrich, T9026, mouse), anti-HA-tag (Santa Cruz Biotechnology, sc-7392 HRP, mouse), anti-phospho-GCN2 (Abcam, ab75837, rabbit), anti-phospho-eIF2alpha (Cell Signaling, 3398, rabbit), anti-eIF2alpha (Cell Signaling, 9722, rabbit), anti-4EBP1 (Cell Signaling, 9644, rabbit), anti-Puromycin (Millipore, MABE343, mouse), anti-Ribosomal protein S10 (Abcam, ab151550, rabbit), anti-phospho-4E-BP1 (Cell Signaling, 2855, rabbit), anti-EDF1 (Abcam, ab174651, rabbit), anti-RPS2 (Bethyl, A303-794A, rabbit), anti-phospho-PERK (Cell Signaling, 3179, rabbit), anti-ASCC3 (Proteintech, 17627-1-AP, rabbit), anti- RPL19 (Novus, H00006143-M01, mouse), anti-phospho-p70 S6 kinase (Cell Signaling Technology, 9234, rabbit), anti-p70 S6 kinase (Cell Signaling Technology, 9202, rabbit).

#### RT-qPCR analysis

Total RNA was purified using TRIzol reagent (Thermo Fisher Scientific, 15596026) according to the manufacturer’s instructions. For reverse transcription 1000 ng of purified RNA was used with Oligo(dT) and RevertAid RT Transcription Kit (Thermo Fisher Scientific Cat # K1691) according to manufacturer’s protocol. For qPCR reactions 5 μl of 10-fold diluted cDNA was used together with SensiFAST SYBR green (Bioline) or Power SYBR green master mix (ThermoFisher) according to the manufacturer’s protocol. RNA abundances were deduced from ΔCt values, normalized to actin mRNA abundance, and compared to the corresponding control sample replicate. Primer sequences can be found in key resources table.

#### Puromycin incorporation assays

##### By western blotting

80% confluent cells were treated as shown in the figures for a desired time followed by addition of puromycin (10 μg/ml) to the media for exactly 10 minutes. Afterwards cells were washed with ice cold PBS and immediately lysed. Western blot was performed to visualize puromycin incorporation into nascent polypeptide chains.

##### With click-iT chemistry

Hela cells were seeded in 96-well plates at a density of 1.5x10ê4 cells per cm^2^. After 42 h cells were exposed to starvation media for 3 h with and without ZAKi (1 μM) and GCN2i (1 μM, A-92 Axon Medchem). Cells stimulated with Cycloheximide (50 μg/ml) were used as a negative control. Protein synthesis was measured using Click-iT Plus OPP Alexa Fluor 488 protein synthesis assay kit (ThermoFisher, C10456). Briefly, cells were incubated with 20 μM Click-iT working solution for 30 min. After one washing step with PBS cells were fixed with 3.7% formaldehyde in PBS and permeabilized with 0.5% Triton X-100 in PBS. After two washing steps with PBS, cells were incubated with Click-iT Plus OPP Alexa 488 reaction cocktail for 30 min protected from light. For DNA staining cells were incubated for 30 min with NuclearMask Blue Stain. Fluorescence intensity of the samples was detected using high-content confocal fluorescent microscopy (ImageXpress Micro Confocal system) at 20× magnification, constant gain and exposure time. Images were analyzed with CellProfiler and intensity of the green channel was used to determine protein synthesis.

#### Sucrose cushions

Crude cellular ribosome fractions were purified by sedimentation through a 30% sucrose cushion. Briefly, cells were lysed (15 mM Tris, pH 7.5, 0.5% NP40, 6 mM MgCl_2_, 300 mM NaCl, RiboLock) and centrifuged at 12,000 g, 4 °C, 10 min. The supernatant was carefully layered onto a sucrose cushion (30% sucrose in 20 mM Tris, pH 7.5, 2 mM MgCl_2_, 150 mM KCl) and ultra-centrifuged at 38,800 rpm for 16 h using Sorvall wX+ Ultrafuge and FIBERlite F50L-8x39 rotor. Pellets were washed thrice in PBS and suspended in 100 mM KCl, 5 mM MgCl_2_, 20 mM HEPES, pH 7.6, 1 mM DTT and 10 mM NH_4_Cl. Purified ribosome fractions were analyzed by SDS-PAGE and western blotting.

#### Ribosome collision assays

After cells were exposed to various treatments, cytosolic lysates were prepared using 20 mM Hepes pH 7.5, 100 mM NaCl, 5 mM MgCl_2_, 100 μg/ml digitonin, 100 μg/ml cycloheximide, 1X protease inhibitor cocktail, 200U NxGen RNase inhibitor. Extracts were incubated on ice for 5 minutes prior to centrifugation at 17,000 g for 5 minutes at 4 °C. After adding calcium chloride to a final concentration of 1 mM, lysates were digested with 500U micrococcal nuclease for 30 minutes at 22 °C. Digestion was terminated by adding 2 mM EGTA. Equivalent amounts of lysate (350-400 mg of RNA) were resolved on 15-50% sucrose gradients. Gradients were subjected to centrifugation at 38,000 rpm in a Sorvall TH64.1 rotor for two hours at 4 °C. The gradients were passed through an ISCO density gradient fractionation system with continuous monitoring of the absorbance at 254 nm. For the amino acid starvation experiments, HeLa cells were starved for 12 hours in RPMI without lysine, arginine and leucine. For the EBSS starvation experiments, HeLa cells were starved for 6 hours in EBSS. For the Torin experiments, U2OS cells were exposed to Torin1 (0.5 μM) for one hour. Where indicated, cells were pre-treated with 1 μM GCN2i (A-92) for 30 minutes.

#### siRNAs

siRNA transfections were carried out using RNAiMAX (Life Technologies, 13778150) following the manufacturer’s instructions. Briefly, cells were seeded onto 6-cm plates and transfected with 200 μM control or target siRNAs. 18 h post transfections siRNAs were washed off and cells were allowed to grow for 48 h before treatment. Sequences used in this study were: ZAKα: 5’- GGTGCCCATTAAGTATCAA (dTdT) and ZAKβ: 5’-CATGCAAGCCAAGCAGAAT (dTdT).

#### Genotyping

##### Genotyping of ZAK^-/-^ mice

Genomic DNA was extracted from ear clippings using QuickExtract DNA Extraction Solution kit (Lucigen, QE09050). PCR product was amplified (Fw: 5’-GCAAGGGGTGAAAATAGGGAG-3’; Rev: 5’-GTGAGTGCTTTCATTTCGACTTG-3’) and digested with EcoRV. KO mutation disrupts a single EcoRV restriction site in the WT product (WT bands: 430 bp and 270 bp; KO band:700 bp).

##### Genotyping of *C. elegans* strains

Mixed stage nematodes were washed off the plates with lysis buffer (20 mM Tris pH 7.5, 50 mM EDTA, 200 mM NaCl, 0,5% SDS, 100 μg/ml Proteinase K) and transferred into a PCR tube. Afterwards, nematodes were freeze-cracked at -80 °C for 30 min and lysed in a thermocycler at 65 °C for 60 minutes followed by proteinase inactivation at 95 °C for 20 min. For genotyping 2 μl of the lysis buffer was used as a template in the PCR reaction. Primer sequences used can be found in the key resources table.

#### *C. elegans* starvation and lifespan

For the starvation survival experiment the worms were synchronized by treating gravid hermaphrodites with an alkaline hypochlorite solution and the surviving embryos were allowed to hatch overnight in sterilized M9 buffer. Synchronized L1’s were incubated in 15 ml of sterilized M9 buffer for the times indicated in the Figure 3B. At each time point, an aliquot from each sample was placed on a plate seeded with OP50. The number of worms surviving to adulthood were determined 3 days later. The number of worms from day 0 of starvation was used as control and as the denominator to calculate the percentage of worms recovering after starvation.

For the lifespan analysis synchronized L4 stage worms were transferred to 4 NGM plates, 30 worms per plate and incubates for 24 h to reach adulthood. To avoid overcrowding from progeny the worms were transferred daily to a new plate for the first 4 days. The worms were observed daily for survival by checking their movement and pharyngeal pumping. A worm was assumed dead if it did not respond to light prodding three times.

#### Leucine deprivation in mice

Male mice were 10-12 weeks old when switched to a nutritionally complete, synthetic control diet for 3 weeks. Afterwards the mice were randomly assigned to dietary treatments: either continued nutritionally complete control diet or diet that was lacking the essential amino acid leucine for 9 days. During the experiment body weight and food intake of the mice were monitored. Synthetic nutritionally complete and leucine deficient diets were obtained from Research Diets based on the recipe from Guo and Cavener.^25^ The two synthetic diets were isocaloric and had identical carbohydrate and lipid composition.

#### Glucose tolerance test

For analysis of glucose tolerance, the mice were fasted for 6 h before intraperitoneal injection with 2 g/kg of glucose. Tail blood glucose concentration was measured at different time points as indicated in Figure 3G using Contour XT glucometer. Homeostasis model assessment for insulin resistance (HOMA-IR) index was calculated according to the formula: [fasting glucose levels (mmol/L)] × [fasting serum insulin (μU/mL)]/22.5.

#### ELISA

To measure plasma insulin concentration mice were fasted for 6 h and blood was collected from the tip of the tail. The insulin concentration was measured using a commercially available kit (Crystal Chem, #90082) according to the manufacturer’s instructions. Serum levels of FGF21 and GDF15 were measured using Mouse/Rat FGF-21 Quantikine ELISA Kit and Mouse/Rat GDF15 Quantikine ELISA Kit, respectively (R&D systems) as described in the manufacturer’s protocol.

#### Tissue processing

Mouse livers were snap-frozen in liquid nitrogen and crushed using a tissue pulverizer. Crushed livers were lysed in ice-cold RIPA buffer (65 mM Tris, 150 mM NaCl, 5 mM EDTA, 1% NP40, 0.5% sodiumdeoxycholate, 0.1% SDS, 10% glycerol, pH 7.4) for protein extraction or TRIzol for RNA extraction using steel beads and a TissueLyzer II (QIAGEN, Hilden, Germany). For protein extraction homogenates were centrifuged at 16,000 g for 20 min and lysates were collected and stored at -80 °C for later western blot analyses.

#### Triglyceride extraction and analysis

Lipids were extracted from 100-200 mg of liver tissue by overnight incubation at 55 °C in ethanolic KOH (66.6% of 96% ethanol and 33.3% KOH). Samples were brought to a volume of 1,200 ml by addition of 50% ethanol followed by centrifugation (5 min, maximum speed). 100 ml of supernatant was mixed with 100 ml of 0.5 M MgCl_2_, and samples were kept on ice for 10 min before centrifugation (5 min, maximum speed). The supernatant was transferred to a new tube. Triglycerides in this material, or from undiluted serum, was measured with Thermo Scientific Triglycerides Reagent (Thermo Fisher Scientific, TR22421) according to the manufacturer’s protocol.

#### Ribosome profiling of mouse livers

Liver pieces from control diet-fed or leucine-starved WT mice were collected and flash-frozen in liquid nitrogen. Using 190 mg of pooled frozen livers (from 2-3 individual animals per processed sample), lysates and ribosome footprints were generated by RNase I digestion and purified according to Janich et al.^31^ with minor modifications. Briefly, 2 μg of RNA was separated and excised from a 15% urea-polyacrylamide gel. From the gel slices, RNA was extracted overnight at 4 °C on a rotating wheel before precipitation in isopropanol during 3 h at -20 °C. As described and adapted from McGlincy and Ingolia,^40^ RNA 3’-end repair was carried out with 2 U/μl of T4 PNK (Lucigen) prior to a 2 h adaptor ligation at 25 °C using 1.5 μl of ligase mix (3.3 U/μL of T4 RNA Ligase 1 (NEB), 66.6 U/μl of T4 RNA Ligase 2 Deletion Mutant (Lucigen) and 13.3 U/μL of riboguard (Lucigen)) and 1 μl of a 20 μM 5’-adenylated DNA adaptor (containing sample barcode and an 8N unique molecular identifiers (UMIs)). Adaptor removal was carried out by treating individual libraries with 2.7 U/μl of 5’deadenylase (NEB) and 5-5 U/μL of RecJ exonuclease (Lucigen) for 1 h at 30 °C and 1 h at 37 °C. Using Zymo Clean & Concentrator columns, samples were purified and pooled. Ribosomal RNA was depleted according to siTools Biotech rRNA depletion kit specifications with minor variations: 4 μl of a custom-made riboPOOL was used to deplete rRNA and the clean-up step was done using Zymo Clean & Concentrator columns. Further library preparation steps were performed as described.^40^ The amplification of the library was carried out using i5 (AL3 or AL4) and i7 (NexteraD503 or NexteraD504) primers. The libraries were sequenced on a NovaSeq6000 (Illumina).

#### Histology

Liver, WAT, and BAT tissues were fixed in 10% formalin solution, (Sigma, HT501128) for 48 h at 4 °C. Fixed tissues were imbedded in parafilm and sections were stained with hematoxylin and eosin (H/E) and Periodic acid-Schiff (PAS) for histology. Liver images were acquired through a 40X objective. Lipid deposits were identified as white empty areas.

### Quantification and statistical analysis

#### Data representation and statistical analysis

Data in bar and line graphs are presented as mean ± SEM. Statistical analyses were performed in GraphPad Prism 9 applying multiple unpaired t test (Figures S3C–S3E), 2-way (Figures 4A, 4C, 4F, 4G, S4A–S4F, S4J, and S4K) or 3-way ANOVA (Figures 3E, 3F, and 3H). Multiple comparisons were corrected by controlling FDR using the Benjamini, Krieger and Yekutieli method. x, interaction in ANOVA test; ns., non-significant; ^∗^, p < 0.05; ^∗∗^, p < 0.01; ^∗∗∗^, p > 0.001, ^∗∗∗∗^, p < 0.0001.

#### Western blot quantification

Quantification of the western blots bands was carried out using ImageJ software. The intensity of each band was normalized to either total p38 or p150 signal.

#### WAT cell size quantification

For WAT cell size quantification images were acquired through a 20X objective and analyzed with ImageJ Adiposoft plugin, n >500 cells per mouse.

#### Ribosome profiling data analysis

Read mapping was perform following publish protocol.^43^ Briefly, reads were trimmed from adapter sequence using cutadapt (version: 3.5; options: --match-read-wildcards --overlap 8 --discard-untrimmed --minimum-length 30) and quality filtered using fastx_toolkit (version: 0.0.14; options: -Q33 -q 30 -p 90). UMIs were extracted from each read with UMItools (version: 1.0.0git; options: extract --extract-method string --bc-pattern NNNNNNNNCCCCC --3prime --filter-cell-barcode –error-correct-cell). Then, reads where size-selected for single ribosome (monosomes; size 26 to 35). Subsequently, to estimate rRNA and tRNA contamination, reads were mapped to human and mouse rRNA and mouse tRNA databases using bowtie2 (version: 2.3.5; options: -p 2 -L 15 -k 20 --trim5 2). Reads that failed to map to these, were then mapped to the mouse transcript database (Ensembl database v. 100). Barcode demultiplexing was carried out with UMItools (options: group --method=directional --per-cell –read-length) and deduplication with an in-house script. Further analyses were restricted to one transcript per gene. Selection was based on APPRIS database^44^ primary isoform classification. If selection was not conclusive, the longer transcript with primary annotation was selected. Correlation plots were done using chip-seq tools (version: 1.5.5^45^). Metagene plots for start and stop codons were performed using predicted A-sites counts (15nt from read 5’-end) normalized by library total depth. Genes with very strong signal (total of 15 genes) where excluded from this analysis. An analogous analysis was performed on Leu (Figure 3K) counting predicted A-site density distributions from the top 10000 sites with the strongest signal in a window of 200 nt. The amino acid abundance plot (Figure 3K) was done as described in Rooijers et al.^46^ Briefly, for each of the 64 codons the total number of occurrences for the predicted A-sites was counted in the control and treated sample, and the relative frequencies were calculated and averaged by amino acid. The log_2_ values of the frequency ratios were plotted. Single codon pausing scores and odds ratios (Figure S4G) were calculated as described,^32^ but with codon counts that represented the aggregated counts of the 3 codons of the decoding center (predicted E-/P-/A-sites). Background amino acid frequency estimation was calculated on all expressed transcripts.

## Data Availability

• RNA sequencing raw data files have been deposited in NCBI’s Gene Expression Omnibus (GEO) archive with the accession code GEO: GSE205191 and are publicly available as of the date of publication (https://www.ncbi.nlm.nih.gov/geo/query/acc.cgi?acc=GSE205191). An Excel file with all plotted data points as well as a pdf document with all western blot raw scans can be found in Data S1. • The script for data analysis can be downloaded from https://github.com/talponer/StalledRibosomesLeucine as of the date of publication. • Any additional information required to reanalyze the data reported in this paper is available from the lead contact upon request.

## Supplemental information

Document S1. Figures S1–S4

Data S1. Source data 1: A PDF file containing uncropped hi-resolution scans of all the western blots presented in the paper, including those in the supplemental figures. 2: an Excel file containing the values that were used to create all graphs in the paper.

Document S2. Article plus supplemental information
