# Supplementary material for: Ribosome stalling is a signal for metabolic regulation by the ribotoxic stress response
Source: Cell Metab. 2022 Dec 6;34(12):2036–2046.e8. doi: 10.1016/j.cmet.2022.10.011 (PMC9763090; doi:10.1016/j.cmet.2022.10.011)
Supplement: Document S1. Figures S1–S4 [file mmc1.pdf]

Cell Metabolism, Volume *34*

Supplemental information

Ribosome stalling is a signal for metabolic regulation by the ribotoxic stress response

Goda Snieckute, Aitana Victoria Genzor, Anna Constance Vind, Laura Ryder, Mark Stoneley, Sébastien Chamois, René Dreos, Cathrine Nordgaard, Frederike Sass, Melanie Blasius, Aida Rodríguez López, Sólveig Hlín Brynjólfsdóttir, Kasper Langebjerg Andersen, Anne E. Willis, Lisa B. Frankel, Steen Seier Poulsen, David Gatfield, Zachary Gerhart-Hines, Christoffer Clemmensen, and Simon Bekker-Jensen

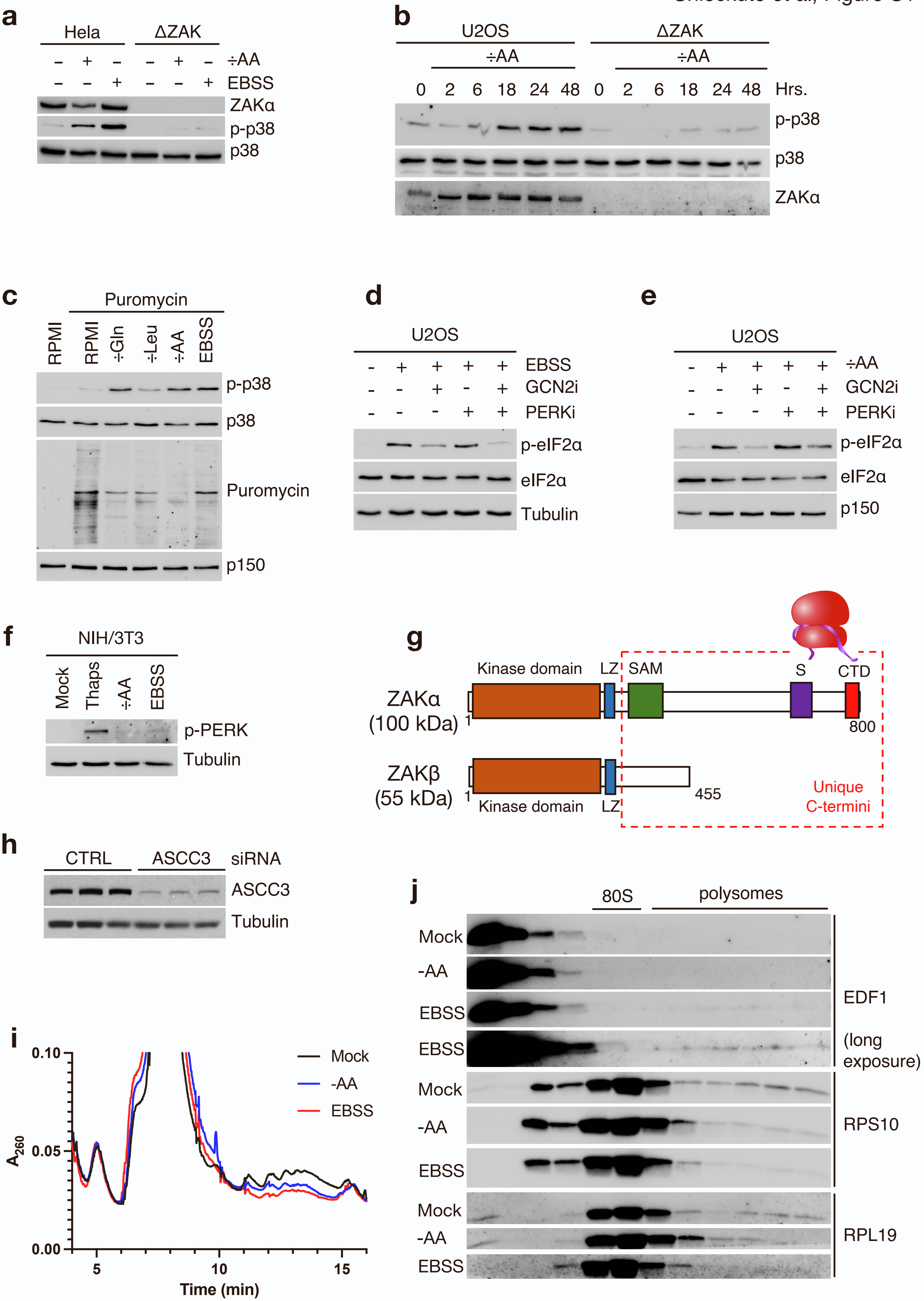
a
Hela
ΔZAK
÷AA
EBSS
ZAKα
p-p38
p38
b
U2OS
ΔZAK
÷AA
0 2 6 18 24 48 0 2 6 18 24 48 Hrs.
p-p38
p38
ZAKα
c
Puromycin
RPMI
RPMI
÷Gln
÷Leu
÷AA
EBSS
p-p38
p38
Puromycin
p150
d
U2OS
EBSS
GCN2i
PERKi
p-eIF2α
eIF2α
Tubulin
e
U2OS
÷AA
GCN2i
PERKi
p-eIF2α
eIF2α
p150
f
NIH/3T3
Mock
Thaps
÷AA
EBSS
p-PERK
Tubulin
g
Kinase domain
LZ
SAM
S
CTD
ZAKα
(100 kDa)
1
800
ZAKβ
(55 kDa)
1
455
Kinase domain
LZ
Unique
C-termini
h
CTRL
ASCC3
siRNA
ASCC3
Tubulin
i
0.10
0.05
0.00
A260
5
10
15
Time (min)
Mock
-AA
EBSS
j
80S
polysomes
Mock
-AA
EBSS
EBSS
EDF1
(long
exposure)
Mock
-AA
EBSS
RPS10
Mock
-AA
EBSS
RPL19

Figure S1.

ZAK-dependent p38 activation upon amino acid deprivation. Related to Figure 1

a. WT Hela cells or Hela cells deleted for ZAK (ΔZAK) were incubated in full RPMI (-), RPMI deficient for leucine, lysine and arginine (÷AA – 12 h) or EBSS starvation medium (12 h). Lysates were analyzed by immunoblotting with the indicated antibodies. **b.** WT U2OS cells or U2OS cells deleted for ZAK (ΔZAK) were incubated in full or ÷AA medium for the indicated times. Lysates were analyzed as in (a). **c.** U2OS cells were incubated in full medium, EBSS medium, ÷AA medium or medium deficient for glutamine (÷Gln) or leucine (÷Leu), respectively, for 18 h. Puromycin (10 µg/ml) was added to the culture 10 min prior to harvest and lysates were analyzed by immunoblotting with anti-puromycin antibodies. **d.** U2OS cells were incubated in EBSS medium (18 h) in the presence of inhibitors (i – 1 µM) of GCN2 and PERK. Lysates were analyzed as in (a). **e.** As in (d), except that cells were incubated in ÷AA medium. **f.** Murine NIH/3T3 cells were treated with thapsigargin (1 µM – 1 h) or incubated in EBSS or ÷AA medium (18 h). Lysates were analyzed as in (a). **g.** Schematic of ZAK protein isoforms. LZ, Leucine Zipper; SAM, Sterile Alpha-Motif; S, Sensor Domain; CTD, C-Terminal Domain. **h.** Hela cells were transfected with CTRL siRNA or siRNAs targeting ASCC3. Lysates were analyzed as in (a). **i.** Hela cells were incubated in EBSS or ÷AA medium (18 h). Lysates were passed through a sucrose gradient to separate monosome- and polysome-containing fractions. **j.** Fractions from (i) were analyzed for ribosome-associated content by immunoblotting.

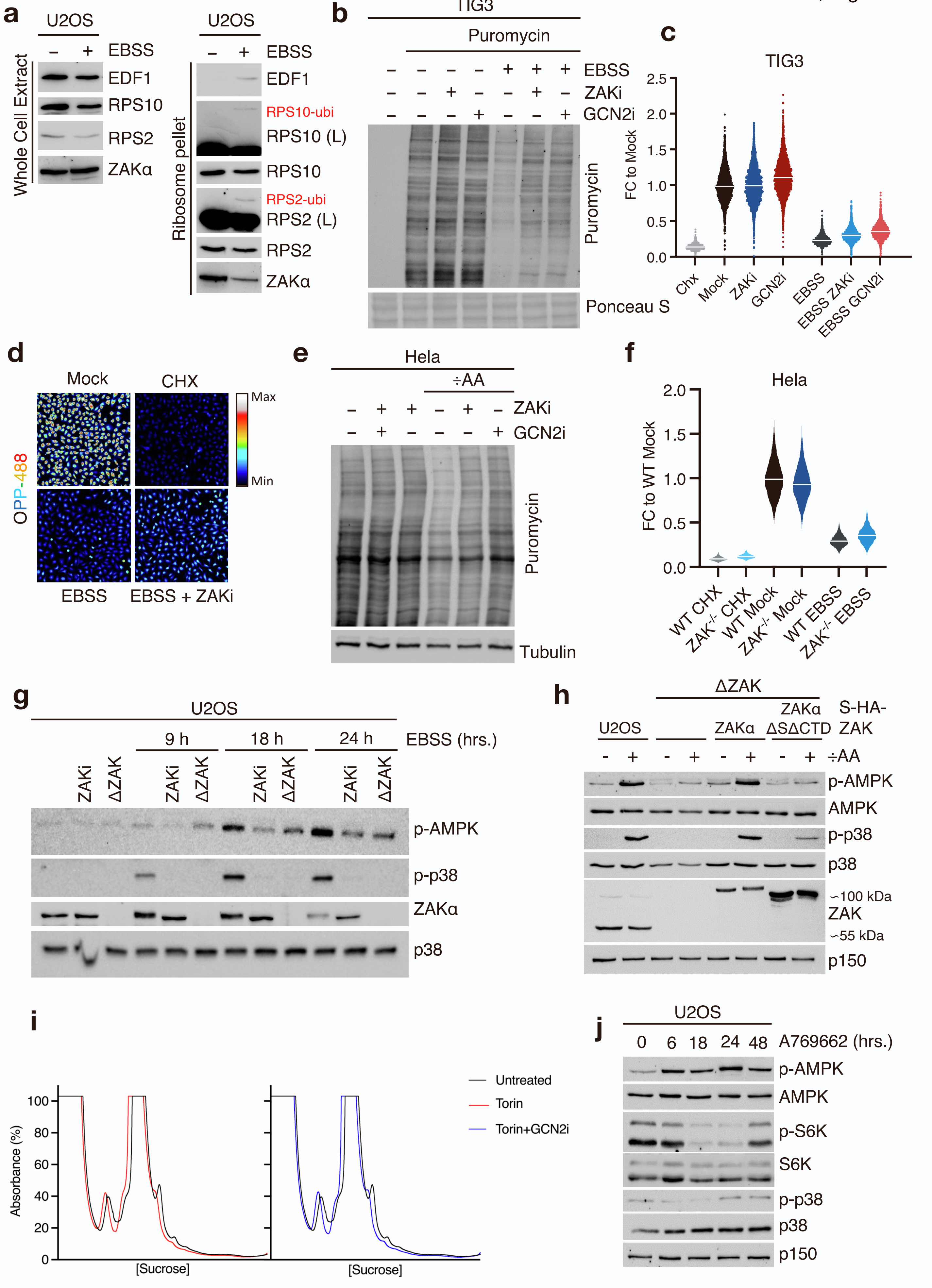
a
U2OS
– + EBSS
Whole Cell Extract
EDF1
RPS10
RPS2
ZAKα
U2OS
– + EBSS
Ribosome pellet
EDF1
RPS10-ubi
RPS10 (L)
RPS10
RPS2-ubi
RPS2 (L)
RPS2
ZAKα
b
TIG3
Puromycin
EBSS
ZAKi
GCN2i
Puromycin
Ponceau S
c
TIG3
FC to Mock
2.5
2.0
1.5
1.0
0.5
0.0
Chx
Mock
ZAKi
GCN2i
EBSS
EBSS ZAKi
EBSS GCN2i
d
Mock
CHX
Max
Min
OPP-488
EBSS
EBSS + ZAKi
e
Hela
÷AA
ZAKi
GCN2i
Puromycin
Tubulin
f
Hela
FC to WT Mock
2.0
1.5
1.0
0.5
0.0
WT CHX
ZAK-/- CHX
WT Mock
ZAK-/- Mock
WT EBSS
ZAK-/- EBSS
g
U2OS
9 h
18 h
24 h
EBSS (hrs.)
ZAKi
ΔZAK
p-AMPK
p-p38
ZAKα
p38
h
ΔZAK
U2OS
ZAKα
ZAKα ΔSΔCTD
S-HA-ZAK
÷AA
p-AMPK
AMPK
p-p38
p38
~100 kDa
ZAK
~55 kDa
p150
i
Untreated
Torin
Torin+GCN2i
Absorbance (%)
100
80
60
40
20
0
[Sucrose]
[Sucrose]
j
U2OS
0 6 18 24 48 A769662 (hrs.)
p-AMPK
AMPK
p-S6K
S6K
p-p38
p38
p150

Figure S2.

The ribotoxic stress response mediates starvation-induced translational downregulation. Related to Figure 1 and 2.

a. U2OS cells were incubated in EBSS (18 h) and lysates were ultra-centrifuged through a sucrose cushion. Whole cell extract and ribosome pelleted materials were analyzed by immunoblotting with the indicated antibodies. (L): long exposure. **b.** TIG3 cells were incubated in EBSS medium (9 h) in the presence of inhibitors (i – 1 μM) of ZAK and GCN2. Puromycin (10 μg/ml) was added to the culture 10 min prior to harvest and lysates were analyzed by immunoblotting with the indicated antibodies. **c.** TIG3 cells were treated with cycloheximide (chx – 10 mg/ml, 1 h) or incubated in EBSS medium (9 h). Cells were incubated with a puromycin analog (OPP - 100 μM) 30 min prior to fixation. OPP incorporation was visualized by "click-it" chemistry, and the fluorescence signal of individual cells were analyzed by high content microscopy (#cells >4000 per condition). Data is presented as a violin plot and the white lines represent the mean. **d.** Representative images of OPP incorporation from the analysis in (d). **e.** As in (b) except that Hela cells were incubated in medium deficient for leucine, lysine and arginine (÷AA - 9 h). **f.** As in (c), except that WT Hela cells or Hela cells deleted for ZAK (ΔZAK) were used. **g.** U2OS and ΔZAK cells were treated with GCN2i and incubated in EBSS medium (18 h) as indicated. Lysates were analyzed as in (b). **h.** U2OS, ΔZAK and ΔZAK cells rescued with WT and mutated forms of ZAKα were incubated in ÷AA medium (18 h). Lysates were analyzed as in (a). **i.** Hela cells were pre-treated with GCN2i (30 min - right) or not (left) and treated with torin (1 μM) for 1 h. Lysates were digested with micrococcal nuclease, separated on a linear sucrose gradient and ribosomes were detected by UV spectrophotometry. **j.** U2OS cells were incubated in the presence of an AMPK activating compound (A769662 – 0.1 mM) for the indicated times. Lysates were analyzed as in (a).

Snieckute et al, Figure S3

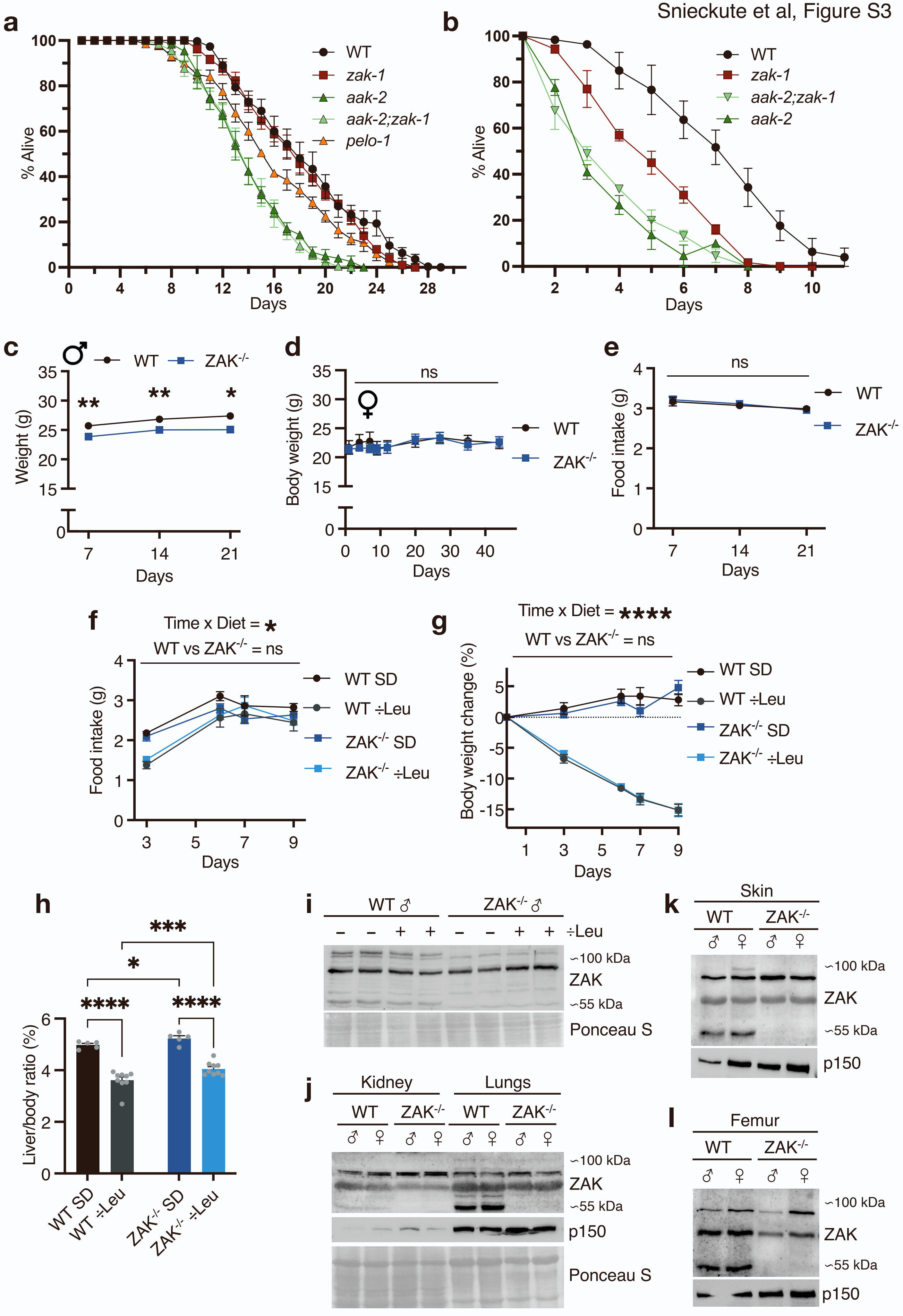

Figure S3.

Model organisms depend on the ribotoxic stress response for metabolic responses to starvation. Related to Figure 3.

a. Lifespan of worms with the indicated genotypes cultivated under normal conditions (20 °C). **b.** Survival curves for worms with the indicated genotypes in M9 starvation medium. (b,c) (n = 3 biological replicates for all strains). **c.** Weight of male mice during the 3 weeks on full synthetic diet (SD). Values indicate the average weight (n = 14 biological replicates). **d.** Body weight of 12-week-old female WT and ZAK$^{-/-}$ mice when fed a complete synthetic diet for 6 weeks. Values indicate average body weight (n = 7 biological replicates). **e.** Food intake of 12-week-old male WT and ZAK$^{-/-}$ mice during a three-week acclimatization from chow to full synthetic diet. Values indicate daily average food intake (n = 14 biological replicates). **f.** Food intake of male mice from (b) upon diet switch as outlined in Figure 3c. Values indicate daily average food intake. **g.** Weight change of mice between diet shift and termination. Values indicate weight gain or -loss for individual mice. **h.** Liver weight from mice in Figure 3c. Values indicate tissue weight as percentage of whole-body weight. **i.** ZAK isoform expression in mouse liver. Snap-frozen livers from four male WT and ZAK$^{-/-}$ mice were crushed, homogenized and analyzed by immunoblotting with the indicated antibodies. **j.** Snap-frozen kidney (left) and lung (right) from one male and female mouse with either WT or ZAK$^{-/-}$ genotype were crushed, homogenized and analyzed by immunoblotting with the indicated antibodies. **k.** As in (j), except that skin was analyzed. **l.** As in (j), except that femur bone was analyzed. (f, g, h) n = 5 for full diet and n = 9 biological replicates for leucine-deficient synthetic diet. All data is plotted as mean and all error bars represent the standard error of the mean (SEM). (c, d, e). ns., non-significant; *, $p<0.05$; **, $p<0.01$ in multiple unpaired t-test using Benjamini, Krieger and Yekutieli FDR. (f, g). x, interaction; ns., non-significant; *, $p<0.05$; ****, $p<0.0001$ in 3-way ANOVA.

Snieckute et al, Figure S4

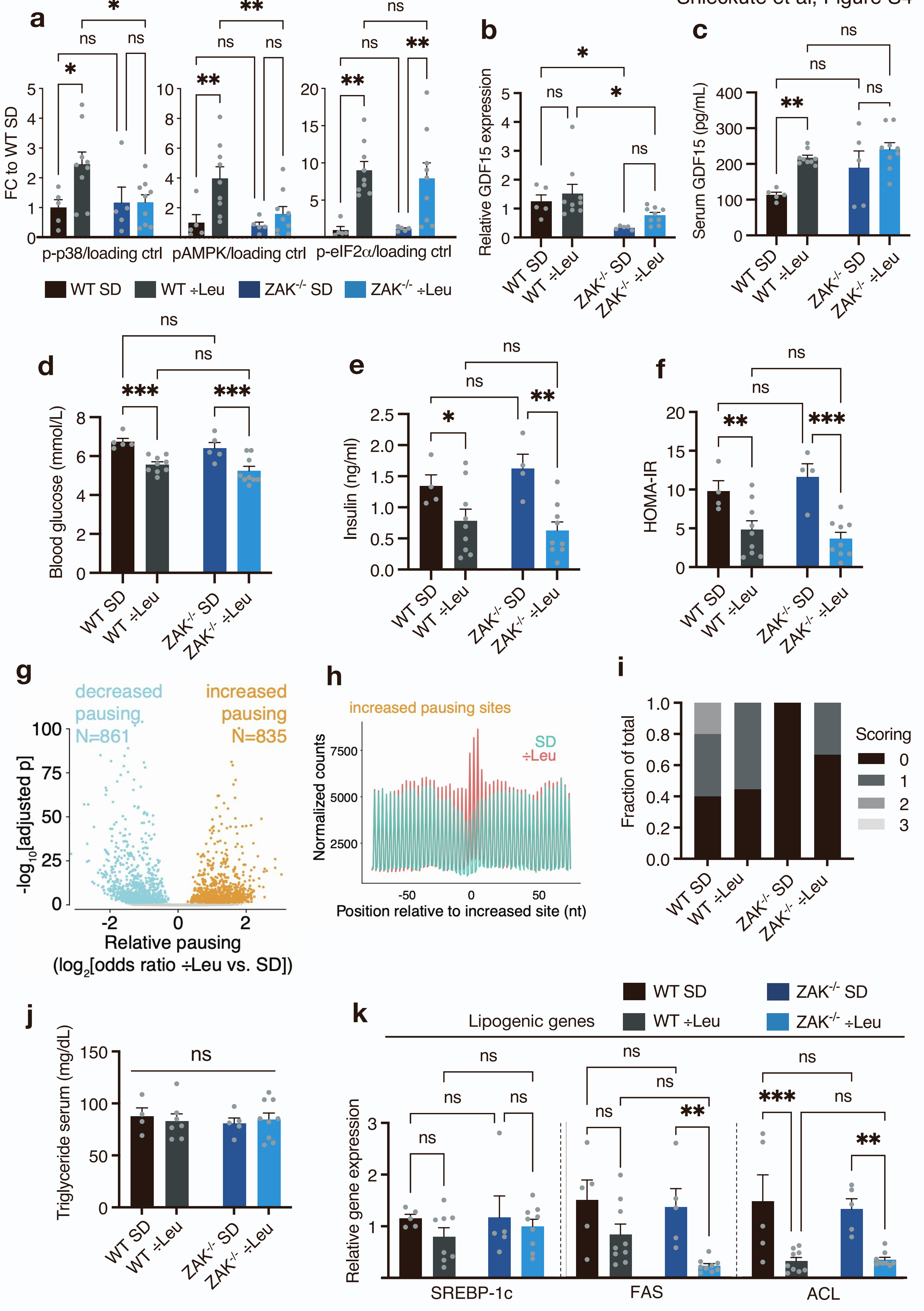

Figure S4.

Leucine starvation of ZAK$^{-/-}$ mice. Related to Figure 3 and 4.

a. Quantification of p-p38, p-AMPK and p-iEF2α signals in immunoblotting analysis of all livers from Figure 3c. Values indicate the average background corrected signal. **b.** qPCR analysis of GDF15 mRNA levels in livers from Figure 3c. Values indicate actin-normalized expression levels normalized to WT. **c.** ELISA-detection of circulating serum levels of GDF15 in mice from Figure 3c. Values indicate the absolute serum concentration. **d.** Fasting blood glucose concentration. **e.** Serum insulin concentration and **f.** HOMA-IR of mice from Figure 3c. **g.** Specific sites with changes in relative ribosome pausing in livers of leucine-deficient vs. SD animals. Codon positions with significantly decreased and increased pause scores (adjusted $p < 0.05$) are indicated in blue (N=865) and orange (N=835), respectively; all other translatome positions in grey. Statistical analysis was performed using a two-sided Fisher's exact test with Benjamini-Hochberg correction. **h.** Metagene analysis similar to Fig. 3i, yet anchoring footprints ("position 0 nt") on the increased sites from Fig. S4g. Counts were normalized by total library depth. **i.** Scoring of liver steatosis grade (scale 0 – 4) of mice from Figure 3c. **j.** Serum triglyceride concentrations of mice in Figure 3c. **k.** qPCR analysis of mRNA levels of lipogenic genes SREBP-1c, FAS and ACL in livers from mice in Figure 3c. Values indicate actin-normalized expression levels normalized to WT. All data is plotted as mean and all error bars represent the standard error of the mean (SEM). ns., non-significant; *, p<0.05; **, p<0.01; ***, p>0.001 in 2-way ANOVA with multiple comparisons using Benjamini, Krieger and Yekutieli FDR.
