# Supplementary material for: Ribosome stalling is a signal for metabolic regulation by the ribotoxic stress response
Source: Cell Metab. 2022 Dec 6;34(12):2036–2046.e8. doi: 10.1016/j.cmet.2022.10.011 (PMC9763090; doi:10.1016/j.cmet.2022.10.011)

Figure 1a

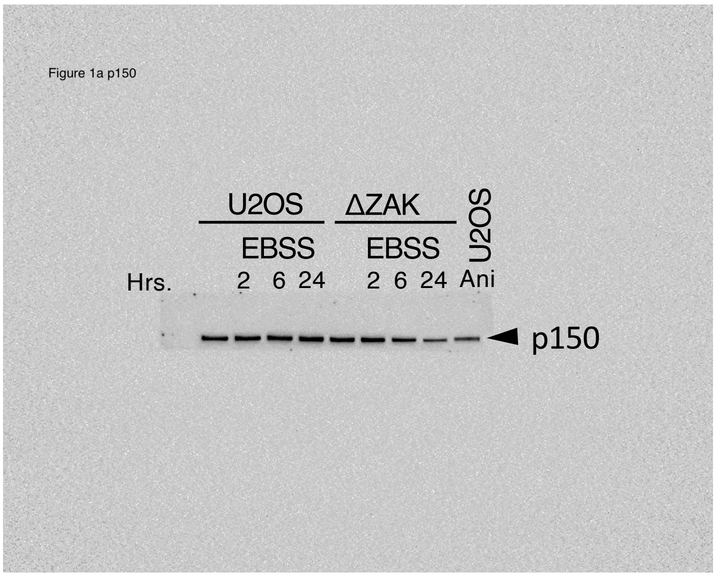
Figure 1a p150
U2OS
ΔZAK
EBSS
EBSS
U2OS
Hrs.
2 6 24
2 6 24
Ani
p150

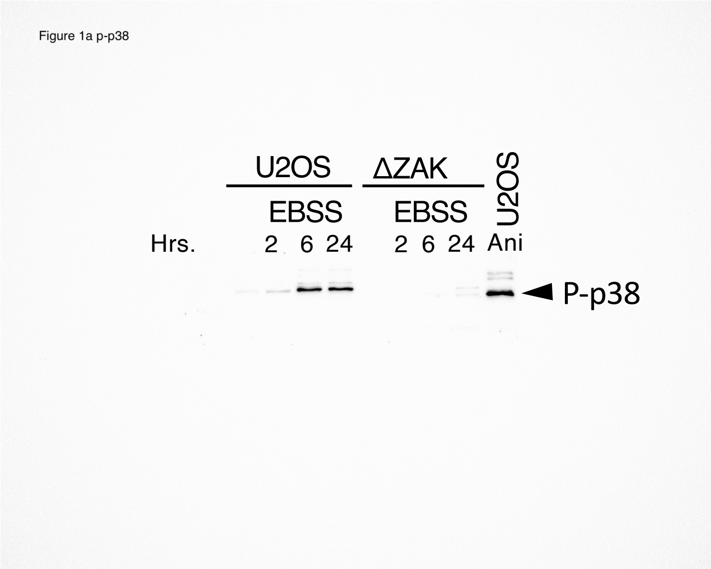
Figure 1a p-p38
U2OS
ΔZAK
EBSS
EBSS
U2OS
Hrs.
2 6 24
2 6 24
Ani
P-p38

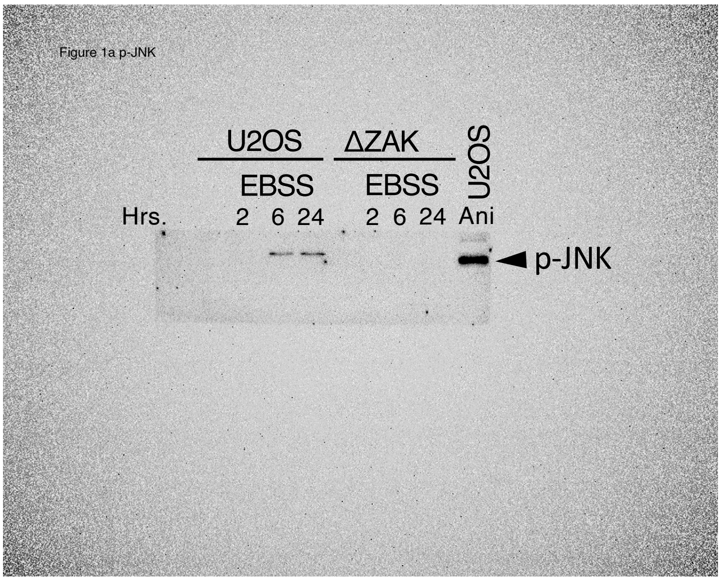
Figure 1a p-JNK
U2OS
ΔZAK
EBSS
EBSS
U2OS
Hrs.
2 6 24
2 6 24
Ani
p-JNK

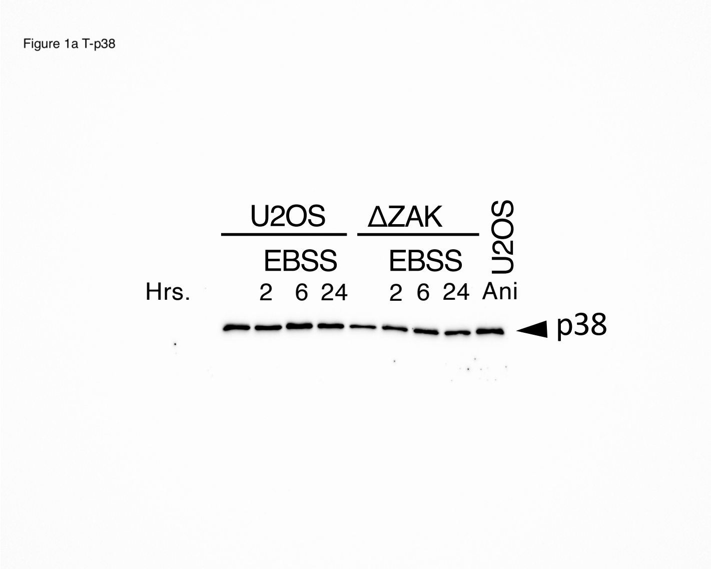
Figure 1a T-p38
U2OS
ΔZAK
EBSS
EBSS
U2OS
Hrs.
2 6 24
2 6 24
Ani
p38

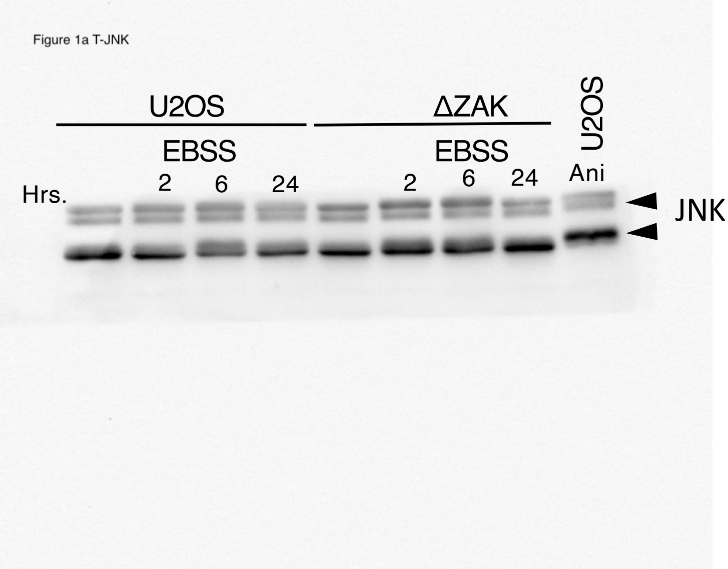
Figure 1a T-JNK
U2OS
ΔZAK
EBSS
EBSS
U2OS
Hrs.
2 6 24
2 6 24
Ani
JNK

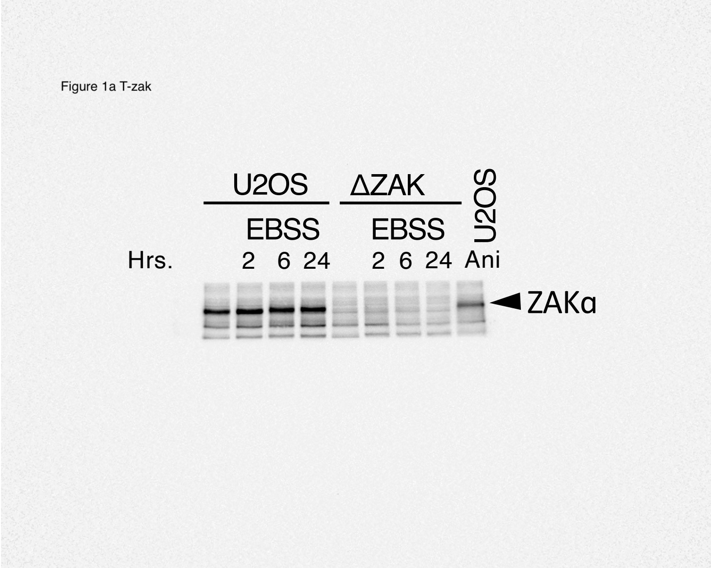
Figure 1a T-zak
U2OS
ΔZAK
EBSS
EBSS
U2OS
Hrs.
2 6 24
2 6 24
Ani
ZAKα

Figure 1b

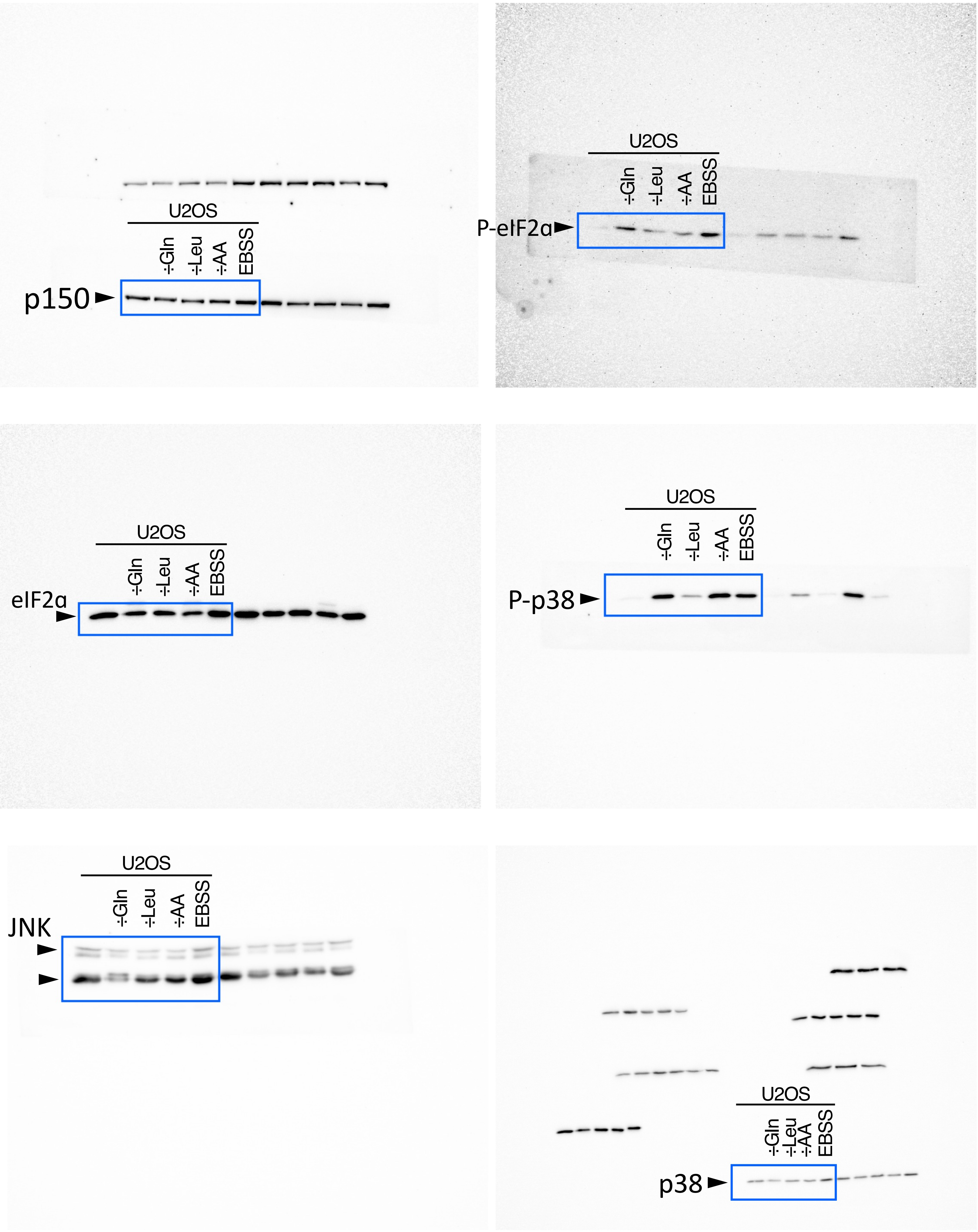
U2OS
÷Gln
÷Leu
÷AA
EBSS
p150
U2OS
÷Gln
÷Leu
÷AA
EBSS
P-eIF2α
U2OS
÷Gln
÷Leu
÷AA
EBSS
eIF2α
U2OS
÷Gln
÷Leu
÷AA
EBSS
P-p38
U2OS
÷Gln
÷Leu
÷AA
EBSS
JNK
U2OS
÷Gln
÷Leu
÷AA
EBSS
p38

Figure 1c

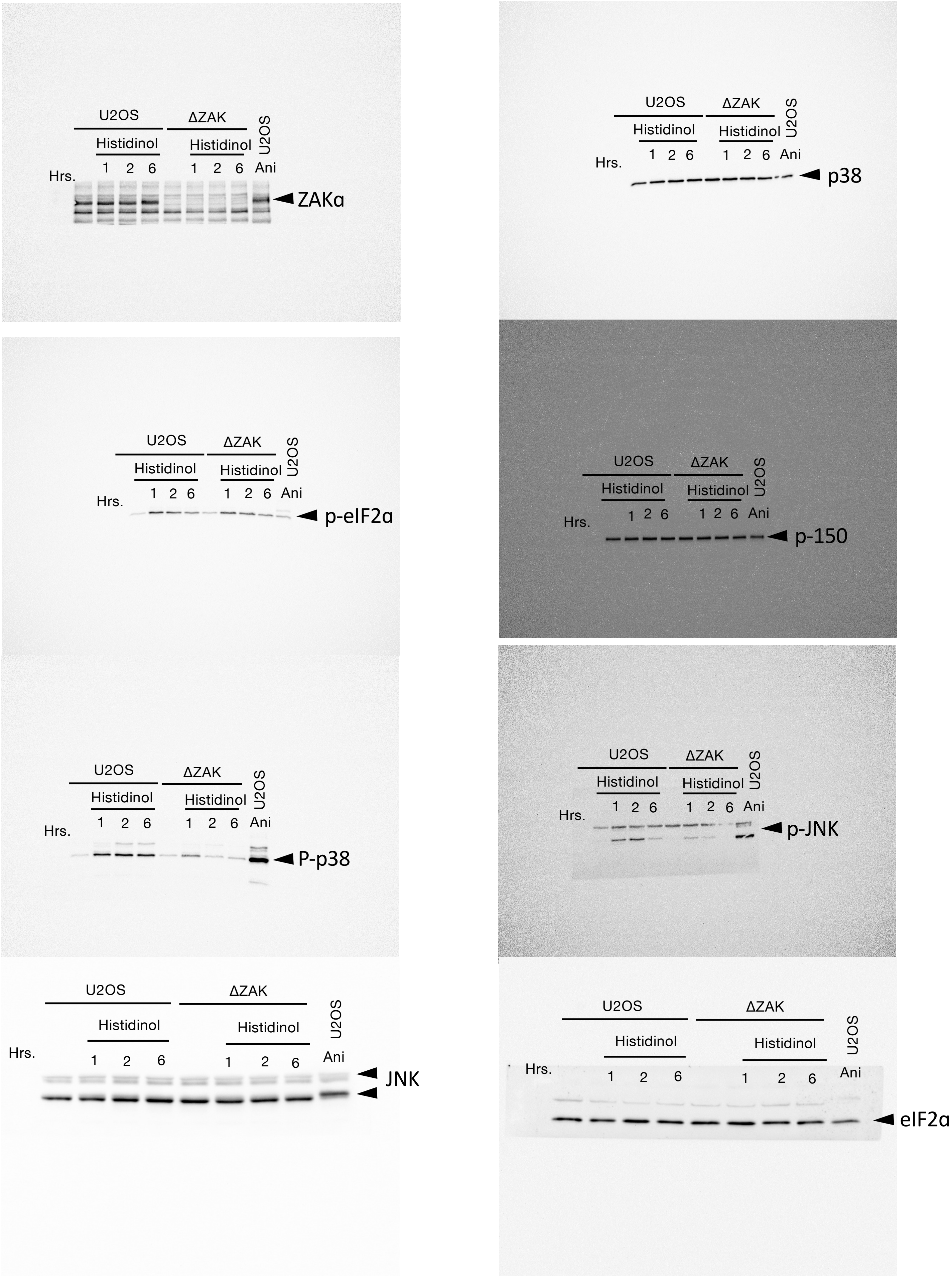

U2OS
ΔZAK
Histidinol
Histidinol
U2OS
Hrs.
1 2 6
1 2 6
Ani
ZAKα
p38
p-eIF2α
p-150
P-p38
p-JNK
JNK
eIF2α

Figure 1d

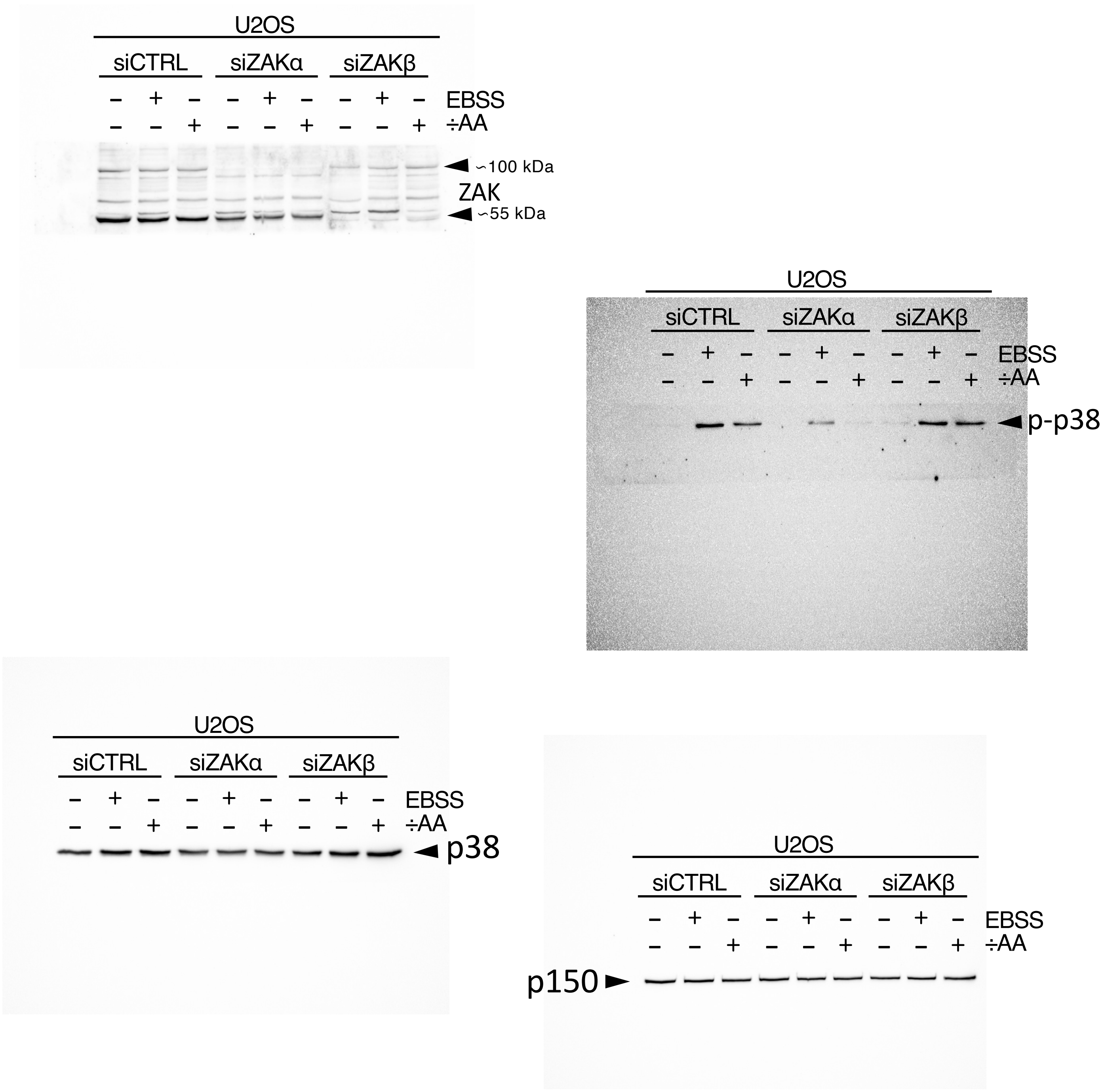

U2OS
siCTRL
siZAKα
siZAKβ
– + – – + – – + – EBSS
– – + – – + – – + ÷AA
~100 kDa
ZAK
~55 kDa
U2OS
siCTRL
siZAKα
siZAKβ
– + – – + – – + – EBSS
– – + – – + – – + ÷AA
p-p38
U2OS
siCTRL
siZAKα
siZAKβ
– + – – + – – + – EBSS
– – + – – + – – + ÷AA
p38
U2OS
siCTRL
siZAKα
siZAKβ
– + – – + – – + – EBSS
– – + – – + – – + ÷AA
p150

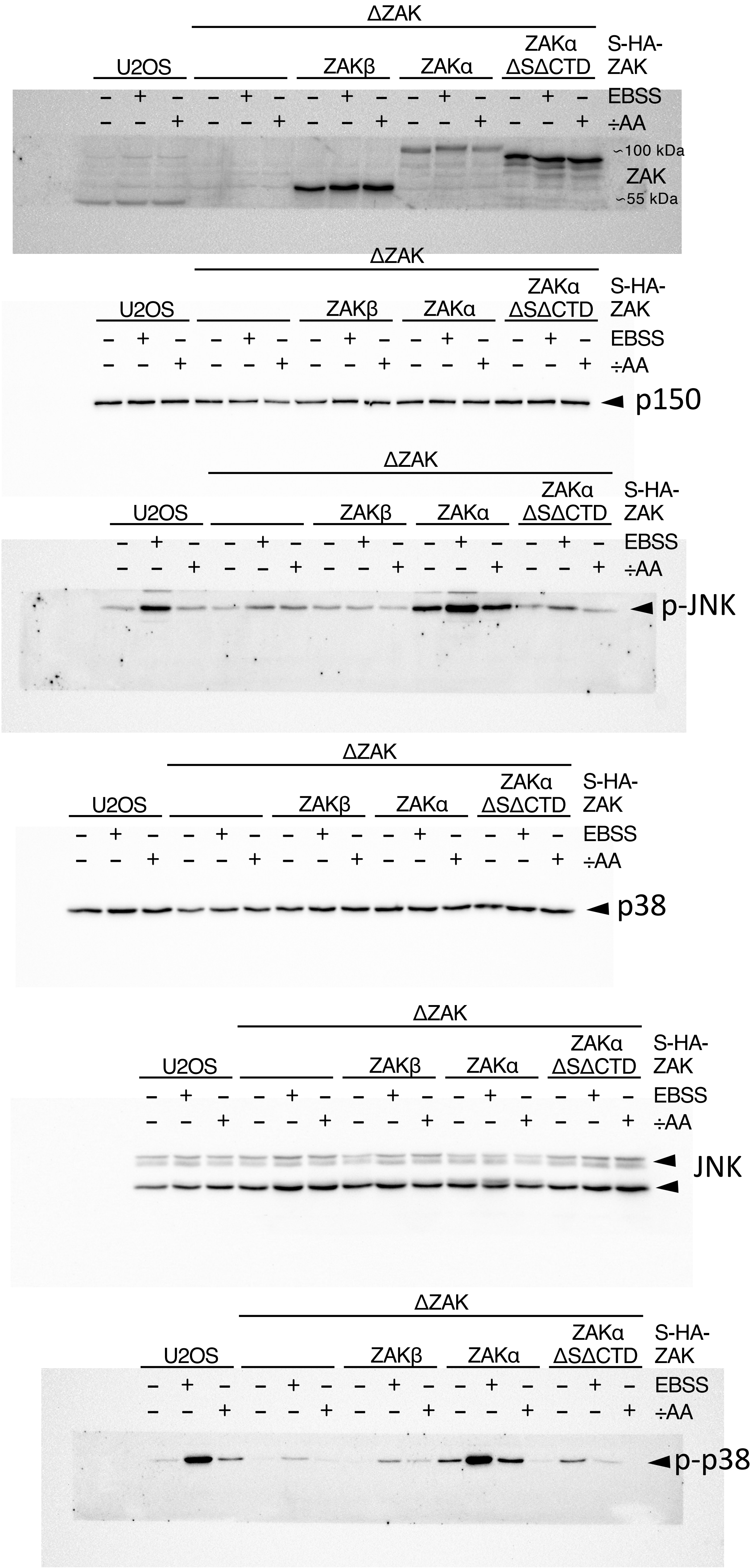
ΔZAK
U2OS
ZAKβ
ZAKα
ZAKα ΔSΔCTD
S-HA-ZAK
EBSS
÷AA
~100 kDa
ZAK
~55 kDa
p150
p-JNK
p38
JNK
p-p38

Figure 1e

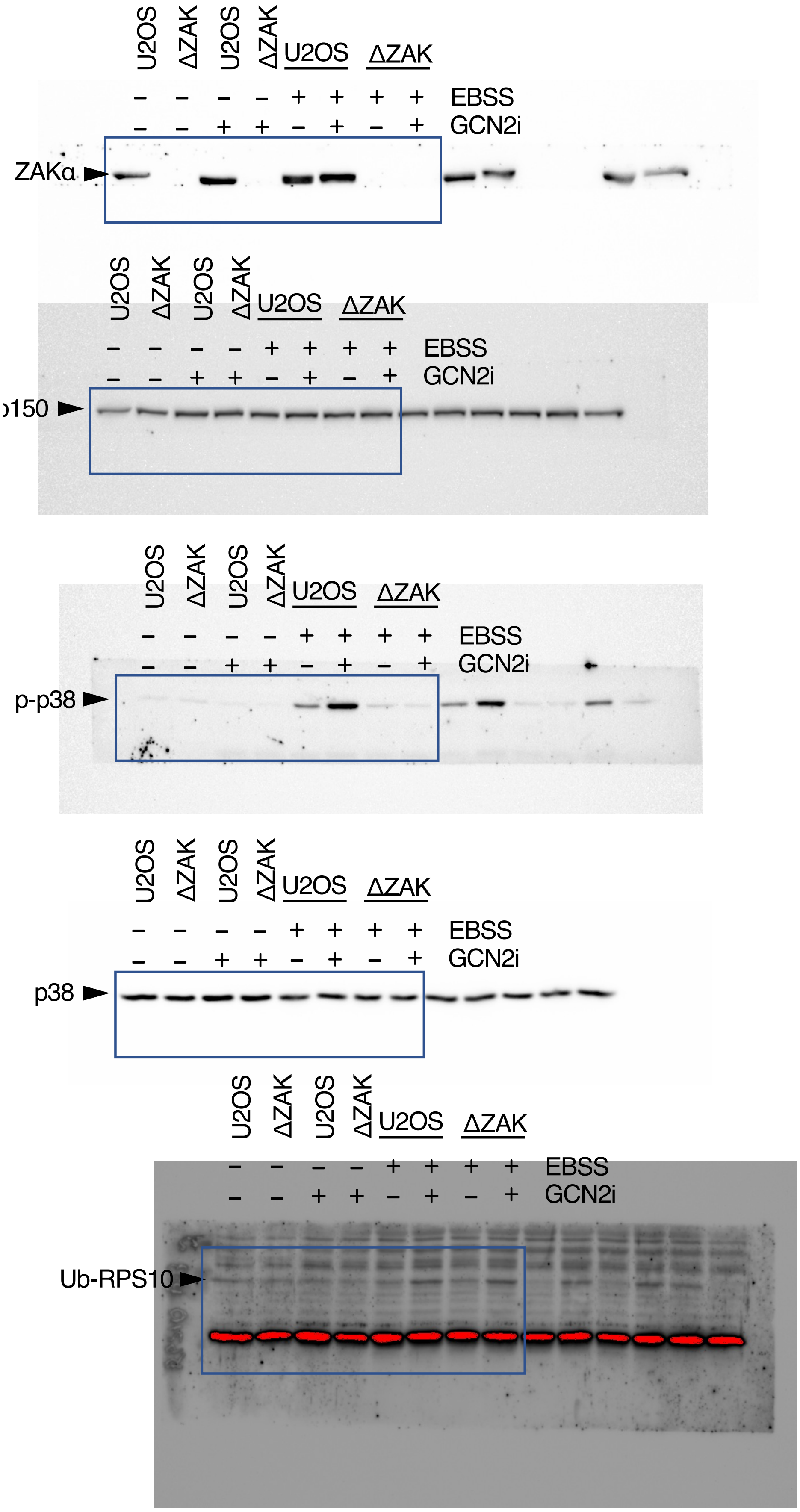
U2OS ΔZAK U2OS ΔZAK U2OS ΔZAK
– – – – + + + + EBSS
– – + + – + – + GCN2i
ZAKα
U2OS ΔZAK U2OS ΔZAK U2OS ΔZAK
– – – – + + + + EBSS
– – + + – + – + GCN2i
p-p38
U2OS ΔZAK U2OS ΔZAK U2OS ΔZAK
– – – – + + + + EBSS
– – + + – + – + GCN2i
p38
U2OS ΔZAK U2OS ΔZAK U2OS ΔZAK
– – – – + + + + EBSS
– – + + – + – + GCN2i
Ub-RPS10

Figure 1h

Figure 2a

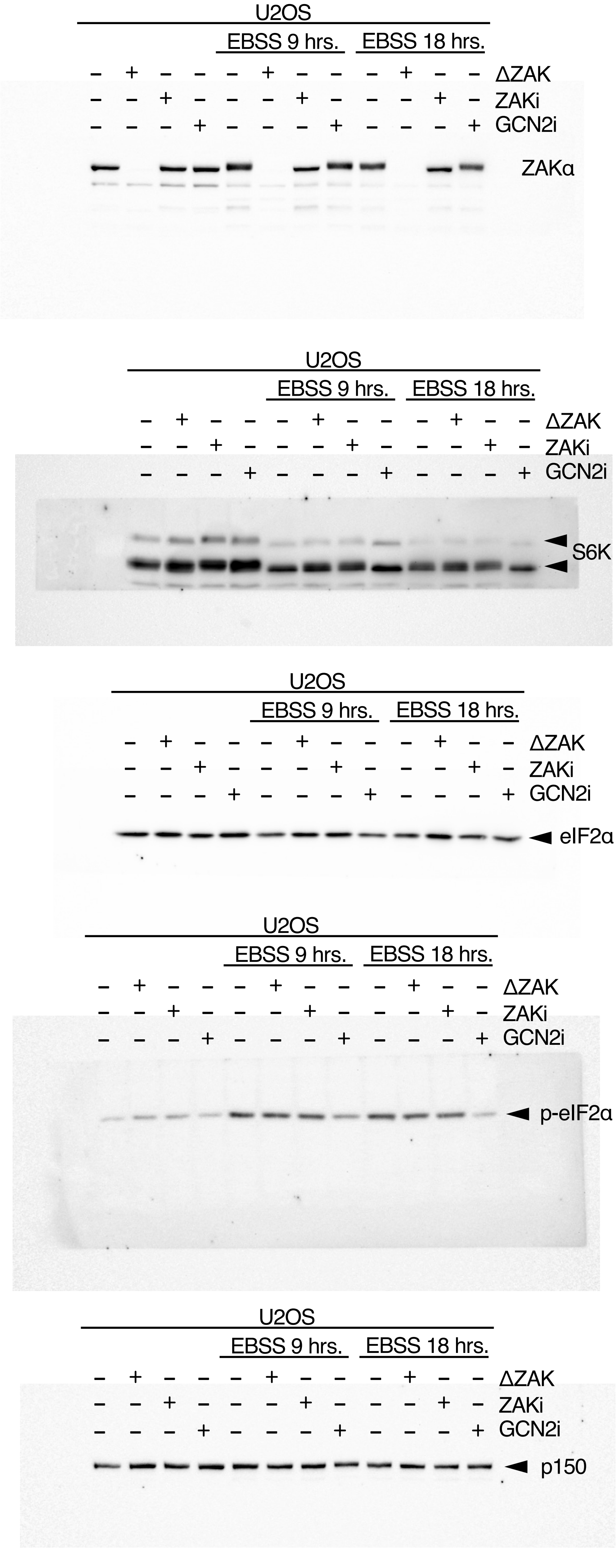
U2OS
EBSS 9 hrs.
EBSS 18 hrs.
– + – – – + – – – + – – ΔZAK
– – + – – – + – – – + – ZAKi
– – – + – – – + – – – + GCN2i
ZAKα
S6K
eIF2α
p-eIF2α
p150

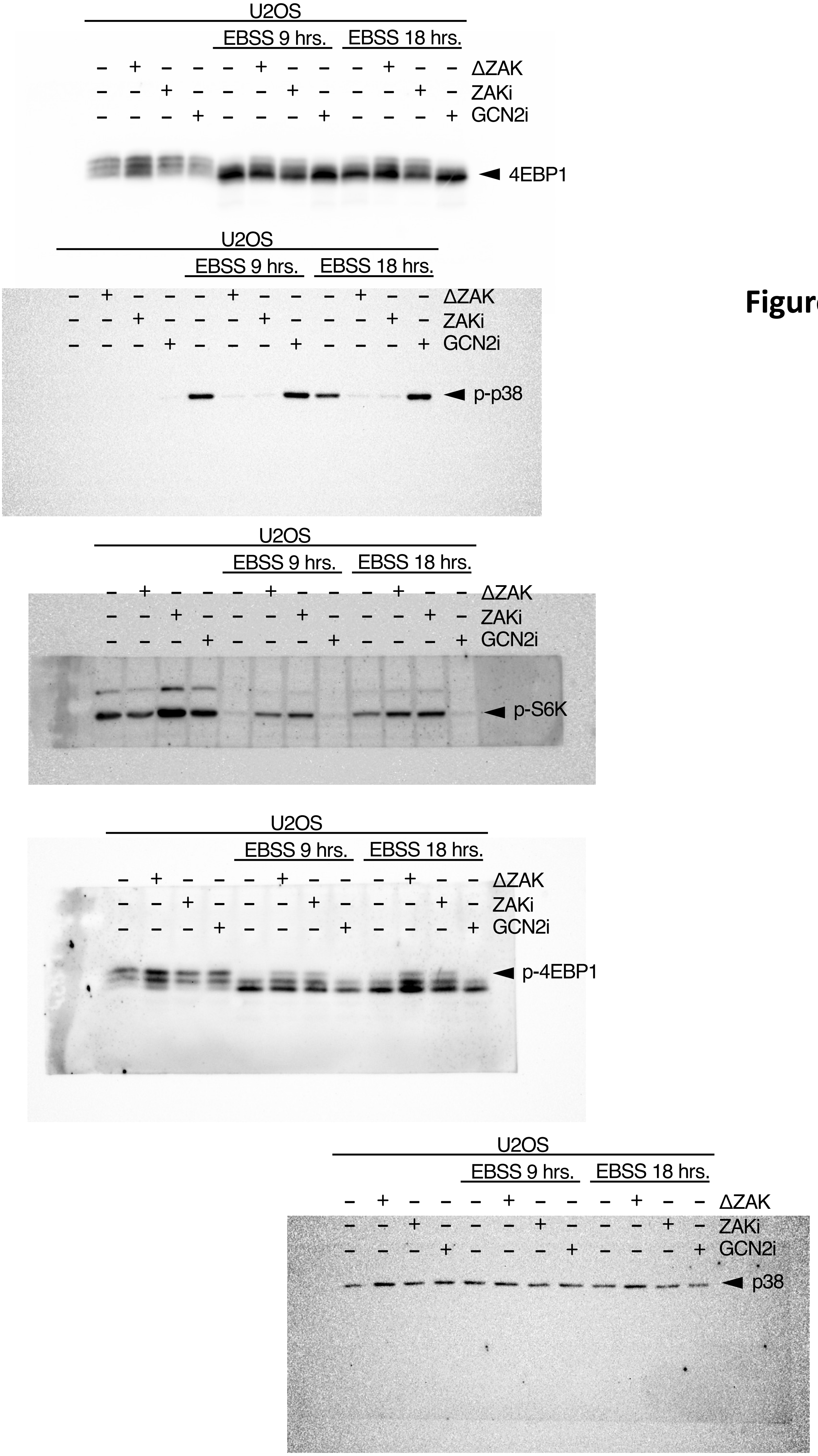
U2OS
EBSS 9 hrs.
EBSS 18 hrs.
– + – – – + – – – + – – ΔZAK
– – + – – – + – – – + – ZAKi
– – – + – – – + – – – + GCN2i
4EBP1
U2OS
EBSS 9 hrs.
EBSS 18 hrs.
– + – – – + – – – + – – ΔZAK
– – + – – – + – – – + – ZAKi
– – – + – – – + – – – + GCN2i
p-p38
U2OS
EBSS 9 hrs.
EBSS 18 hrs.
– + – – – + – – – + – – ΔZAK
– – + – – – + – – – + – ZAKi
– – – + – – – + – – – + GCN2i
p-S6K
U2OS
EBSS 9 hrs.
EBSS 18 hrs.
– + – – – + – – – + – – ΔZAK
– – + – – – + – – – + – ZAKi
– – – + – – – + – – – + GCN2i
p-4EBP1
U2OS
EBSS 9 hrs.
EBSS 18 hrs.
– + – – – + – – – + – – ΔZAK
– – + – – – + – – – + – ZAKi
– – – + – – – + – – – + GCN2i
p38

Figure 2a

Figure 2b

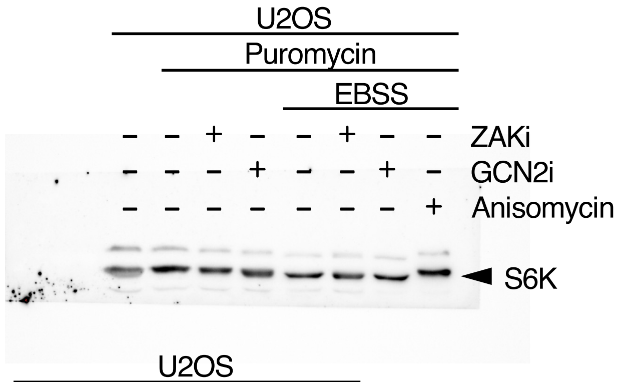
U2OS
Puromycin
EBSS
– – + – – + – – ZAKi
– – – + – – + – GCN2i
– – – – – – – + Anisomycin
S6K

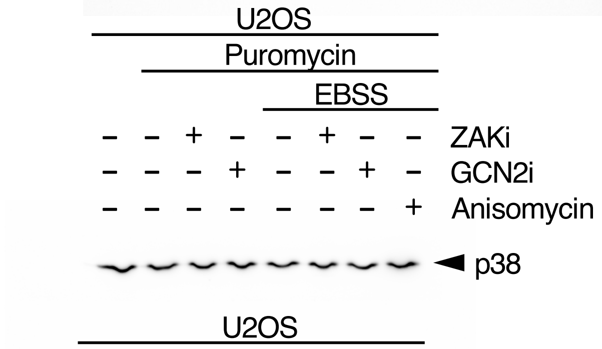
U2OS
Puromycin
EBSS
– – + – – + – – ZAKi
– – – + – – + – GCN2i
– – – – – – – + Anisomycin
p38

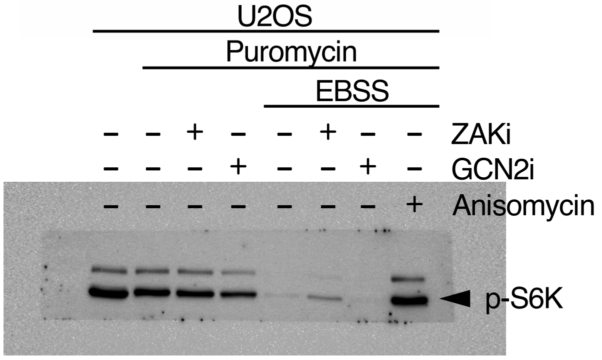
U2OS
Puromycin
EBSS
– – + – – + – – ZAKi
– – – + – – + – GCN2i
– – – – – – – + Anisomycin
p-S6K

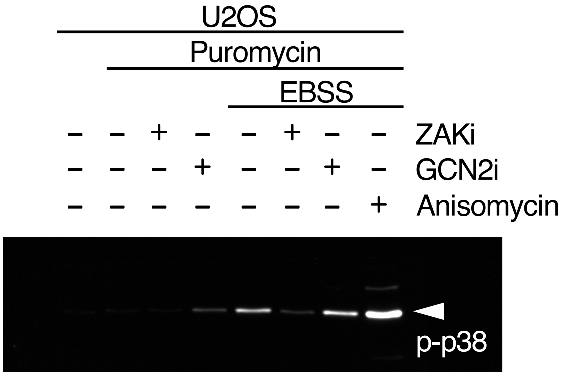
U2OS
Puromycin
EBSS
– – + – – + – – ZAKi
– – – + – – + – GCN2i
– – – – – – – + Anisomycin
p-p38

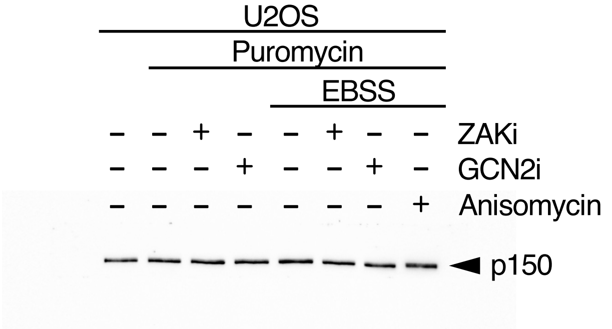
U2OS
Puromycin
EBSS
– – + – – + – – ZAKi
– – – + – – + – GCN2i
– – – – – – – + Anisomycin
p150

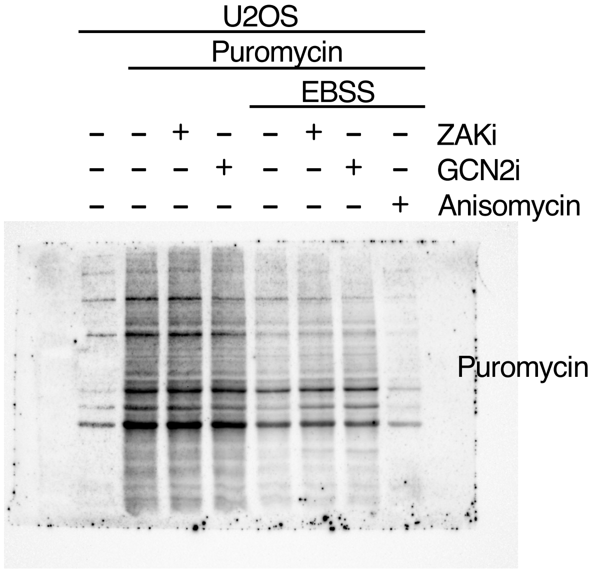
U2OS
Puromycin
EBSS
– – + – – + – – ZAKi
– – – + – – + – GCN2i
– – – – – – – + Anisomycin
Puromycin

Figure 2c

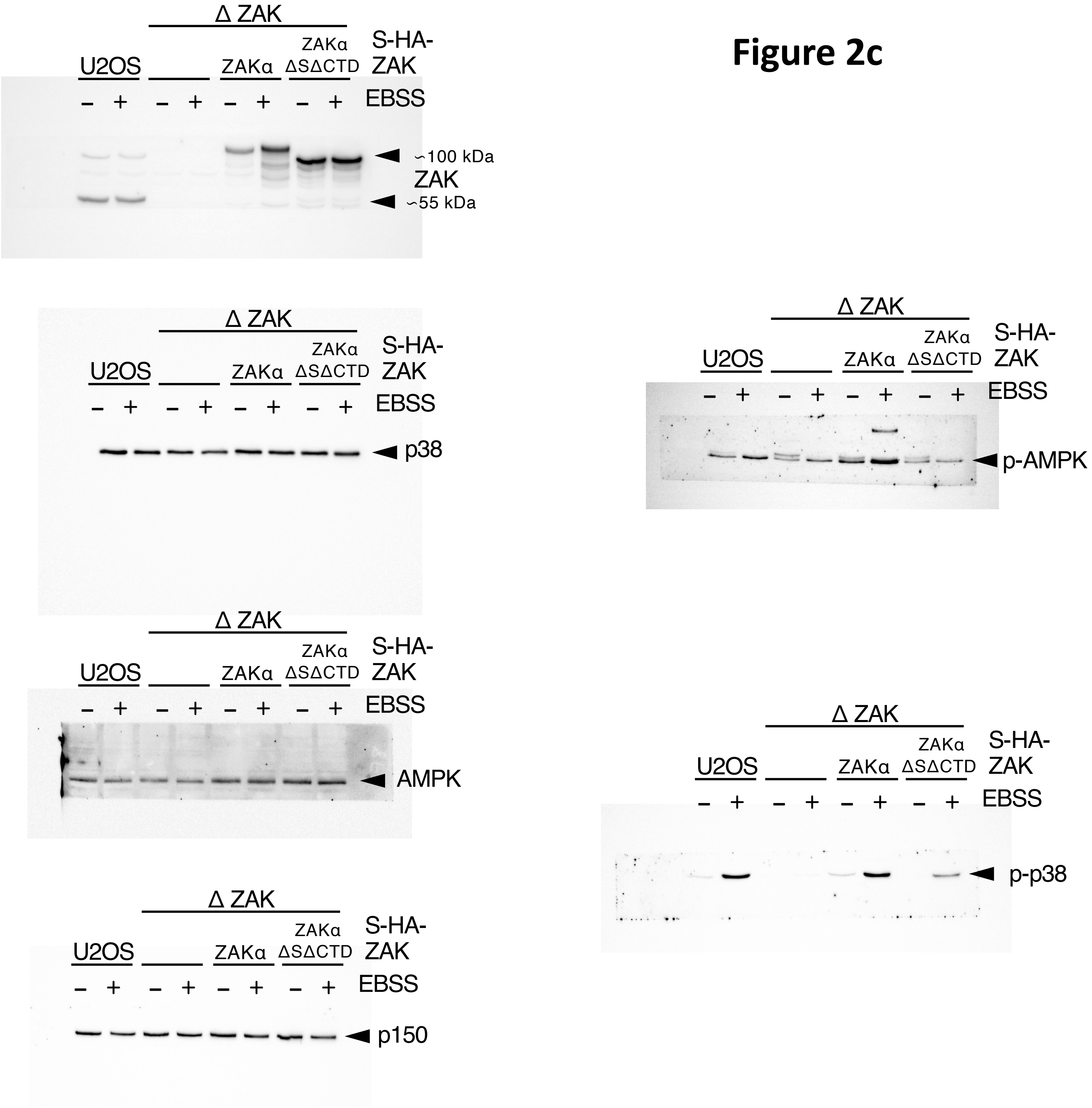

Δ ZAK
U2OS
ZAKα
ZAKα ΔSΔCTD
S-HA-ZAK
EBSS
~100 kDa
ZAK
~55 kDa
p38
p-AMPK
AMPK
p-p38
p150

Figure 2d

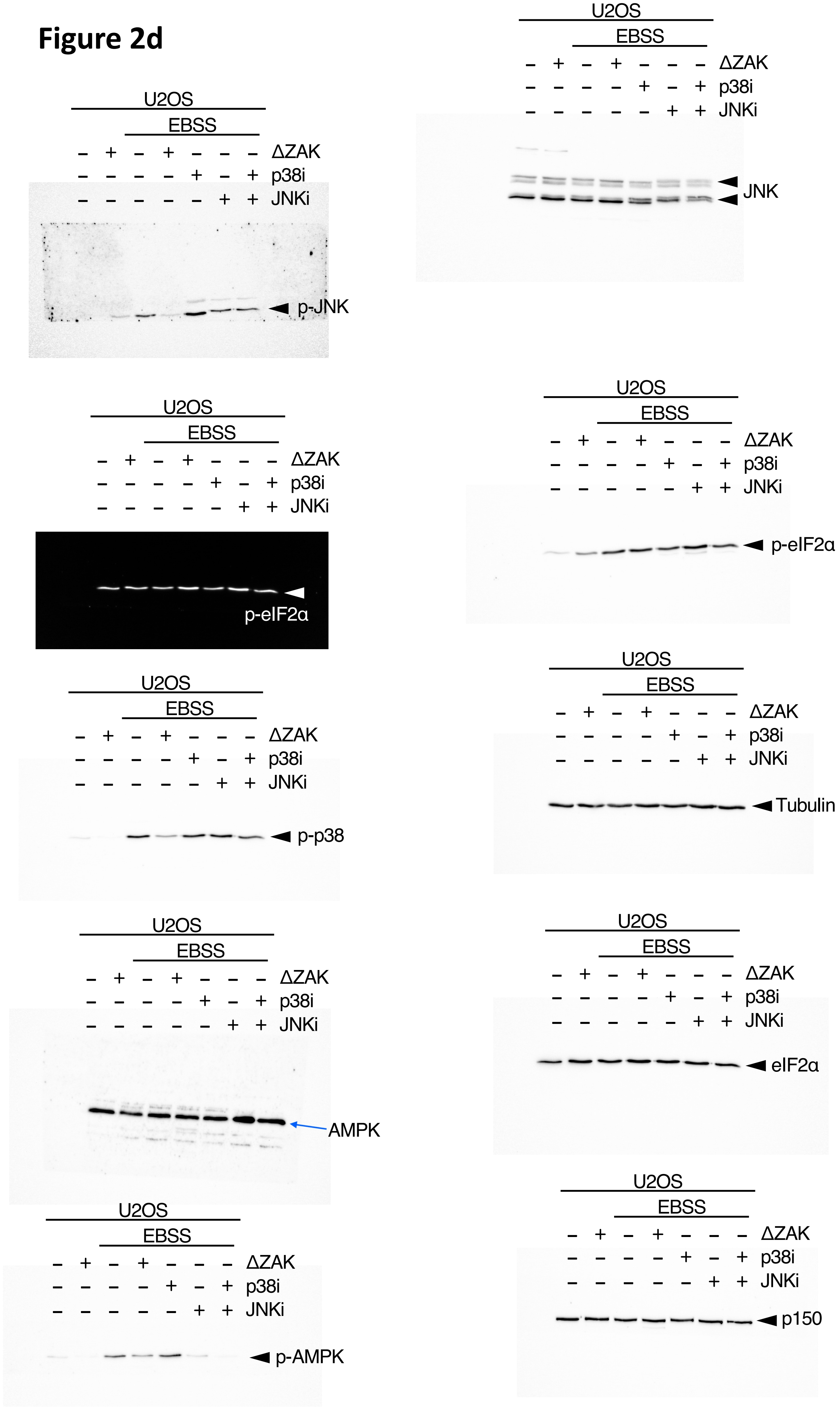
U2OS
EBSS
ΔZAK
p38i
JNKi
p-JNK
U2OS
EBSS
ΔZAK
p38i
JNKi
JNK
U2OS
EBSS
ΔZAK
p38i
JNKi
p-eIF2α
U2OS
EBSS
ΔZAK
p38i
JNKi
p-eIF2α
U2OS
EBSS
ΔZAK
p38i
JNKi
p-p38
U2OS
EBSS
ΔZAK
p38i
JNKi
Tubulin
U2OS
EBSS
ΔZAK
p38i
JNKi
AMPK
U2OS
EBSS
ΔZAK
p38i
JNKi
eIF2α
U2OS
EBSS
ΔZAK
p38i
JNKi
p-AMPK
U2OS
EBSS
ΔZAK
p38i
JNKi
p150

Figure 2e

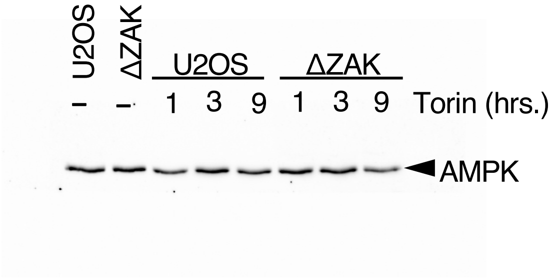
U2OS
ΔZAK
U2OS
ΔZAK
– – 1 3 9 1 3 9 Torin (hrs.)
AMPK

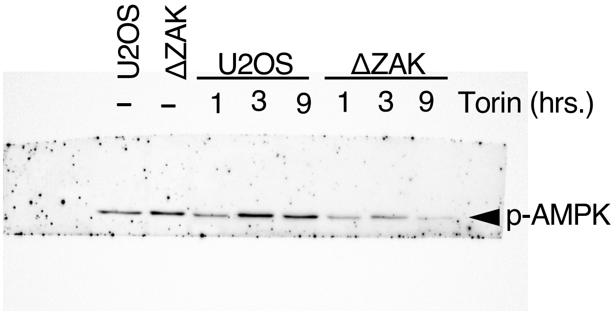
U2OS
ΔZAK
U2OS
ΔZAK
– – 1 3 9 1 3 9 Torin (hrs.)
p-AMPK

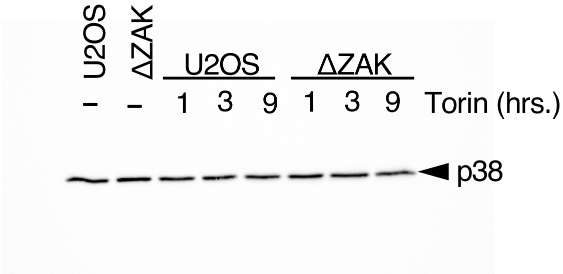
U2OS
ΔZAK
U2OS
ΔZAK
– – 1 3 9 1 3 9 Torin (hrs.)
p38

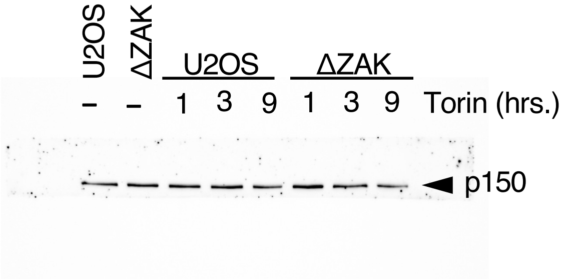
U2OS
ΔZAK
U2OS
ΔZAK
– – 1 3 9 1 3 9 Torin (hrs.)
p150

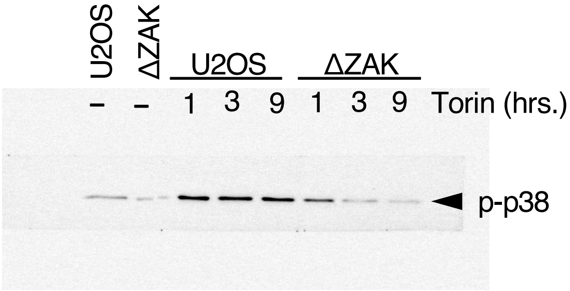
U2OS
ΔZAK
U2OS
ΔZAK
– – 1 3 9 1 3 9 Torin (hrs.)
p-p38

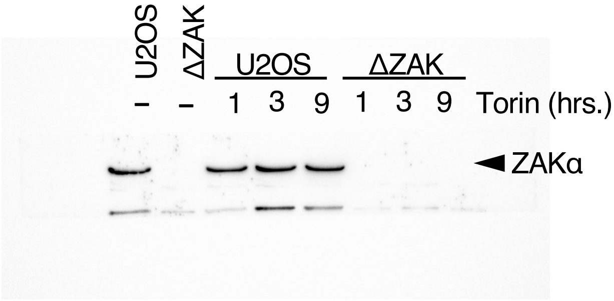
U2OS
ΔZAK
U2OS
ΔZAK
– – 1 3 9 1 3 9 Torin (hrs.)
ZAKα

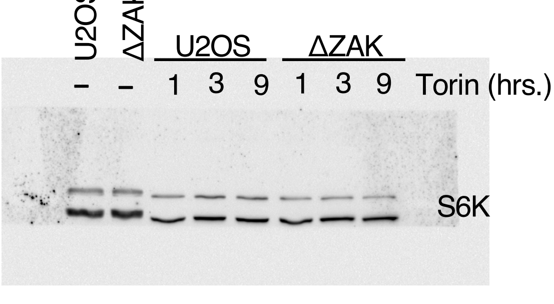
U2OS
ΔZAK
U2OS
ΔZAK
– – 1 3 9 1 3 9 Torin (hrs.)
S6K

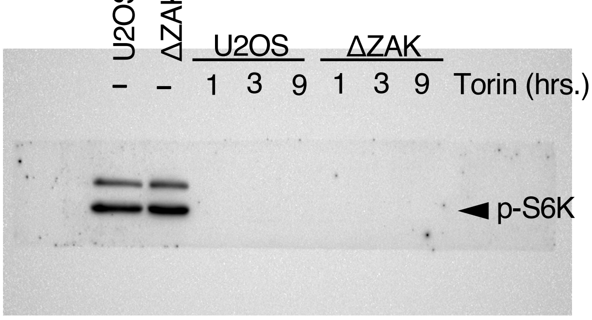
U2OS
ΔZAK
U2OS
ΔZAK
– – 1 3 9 1 3 9 Torin (hrs.)
p-S6K

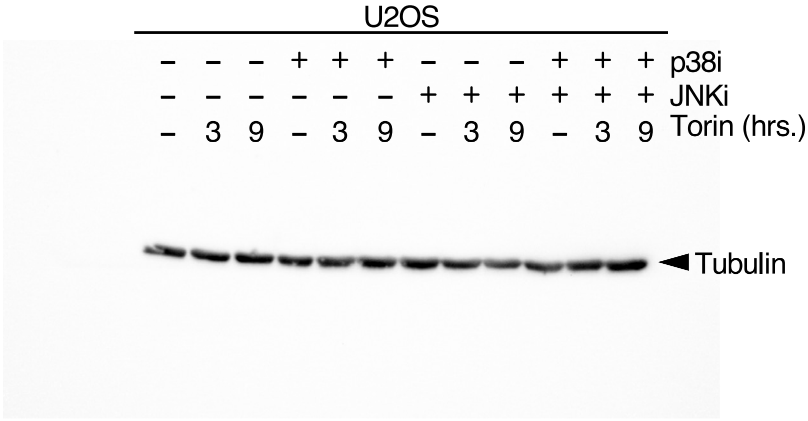
U2OS
– – – + + + – – – + + + p38i
– – – – – – + + + + + + JNKi
– 3 9 – 3 9 – 3 9 – 3 9 Torin (hrs.)
Tubulin

Figure 2f

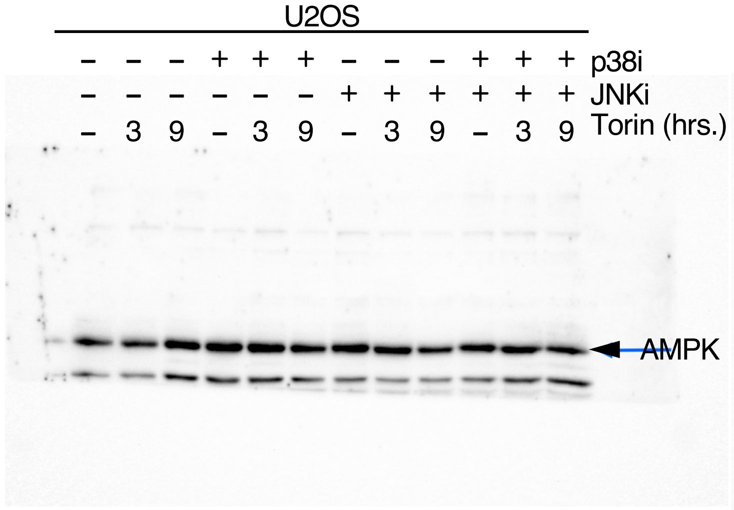
U2OS
– – – + + + – – – + + + p38i
– – – – – – + + + + + + JNKi
– 3 9 – 3 9 – 3 9 – 3 9 Torin (hrs.)
AMPK

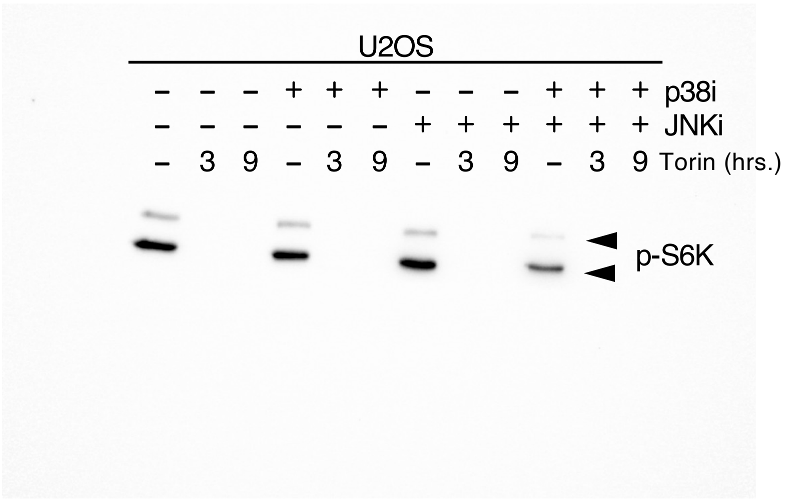
U2OS
– – – + + + – – – + + + p38i
– – – – – – + + + + + + JNKi
– 3 9 – 3 9 – 3 9 – 3 9 Torin (hrs.)
p-S6K

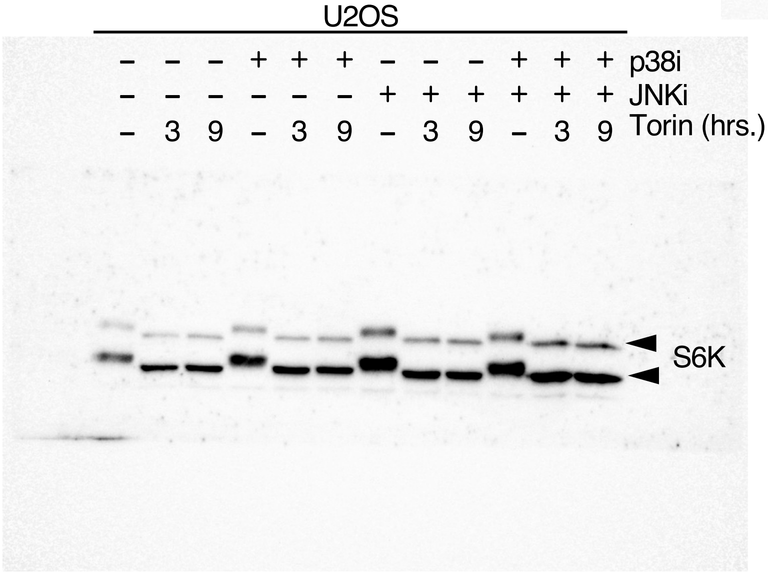
U2OS
– – – + + + – – – + + + p38i
– – – – – – + + + + + + JNKi
– 3 9 – 3 9 – 3 9 – 3 9 Torin (hrs.)
S6K

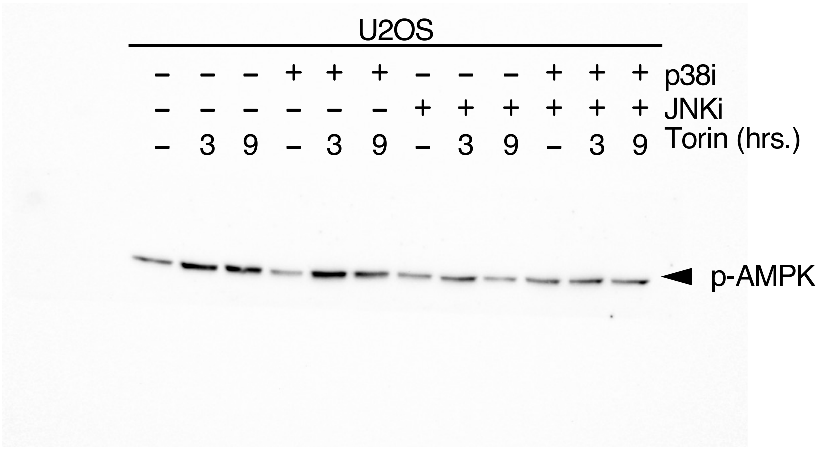
U2OS
– – – + + + – – – + + + p38i
– – – – – – + + + + + + JNKi
– 3 9 – 3 9 – 3 9 – 3 9 Torin (hrs.)
p-AMPK

Figure 2g

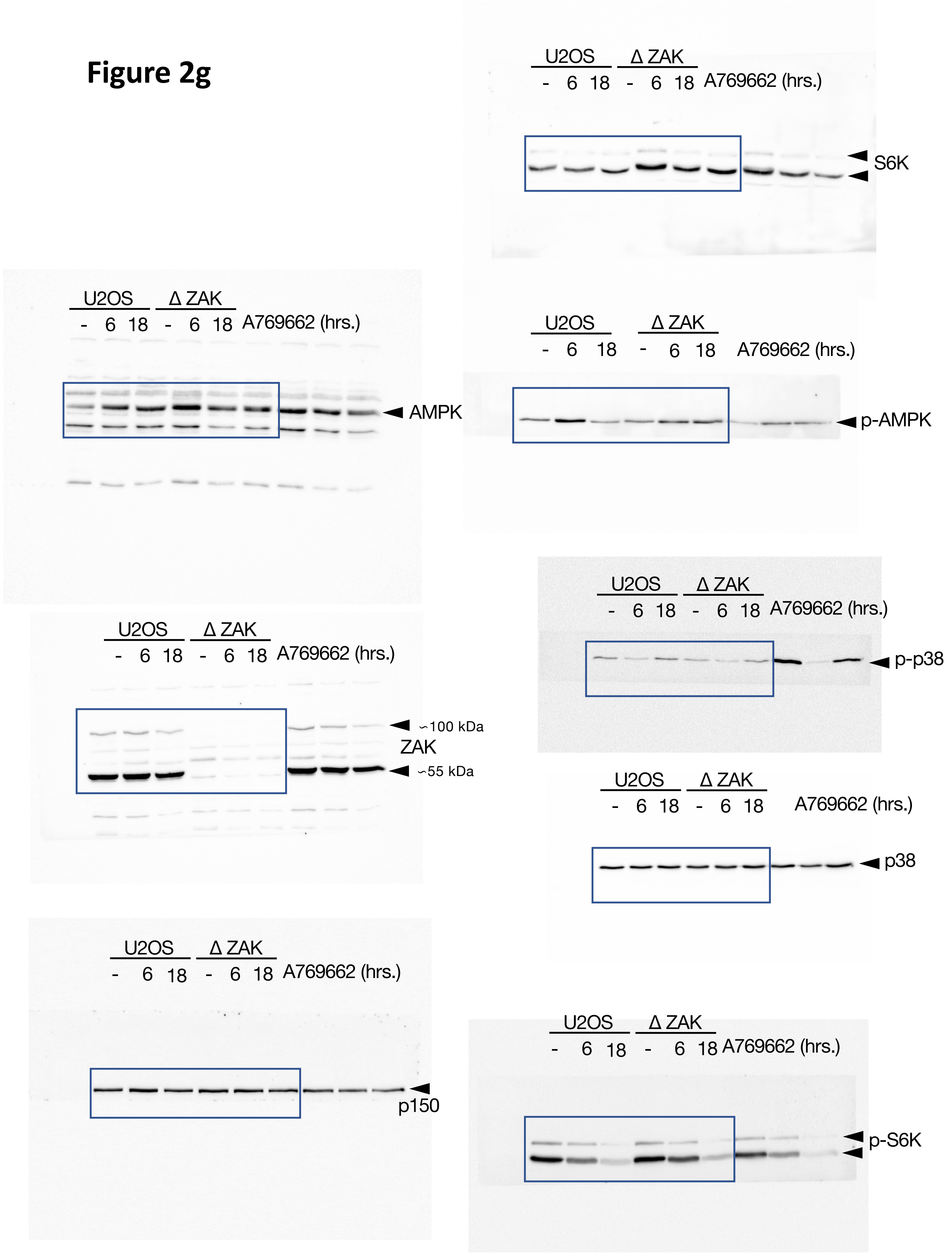
U2OS
Δ ZAK
- 6 18 - 6 18 A769662 (hrs.)
S6K
AMPK
p-AMPK
p-p38
~100 kDa
ZAK
~55 kDa
p38
p150
p-S6K

Figure 3d

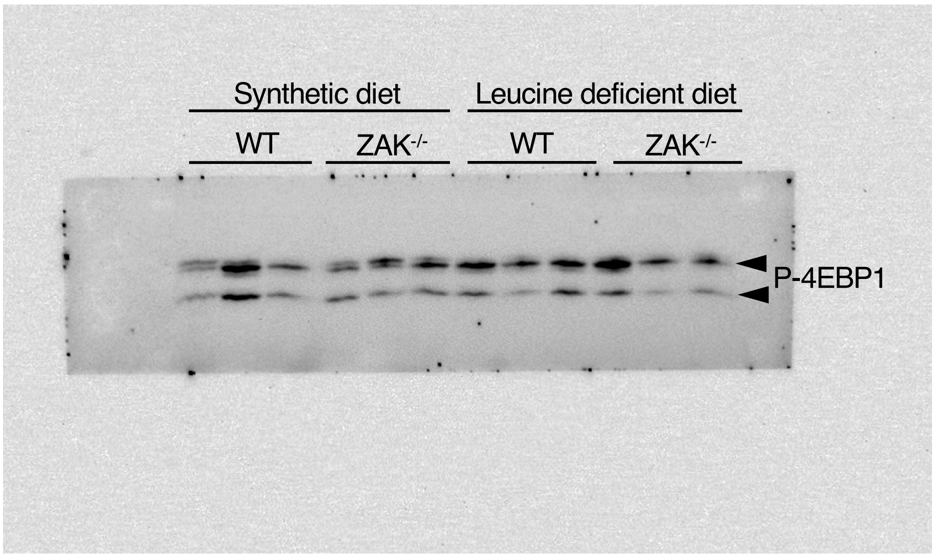
Synthetic diet
Leucine deficient diet
WT
ZAK-/-
WT
ZAK-/-
P-4EBP1

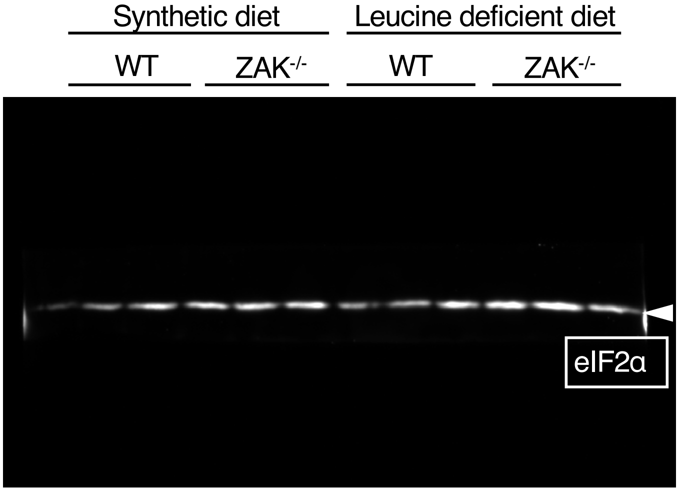
Synthetic diet
Leucine deficient diet
WT
ZAK-/-
WT
ZAK-/-
eIF2α

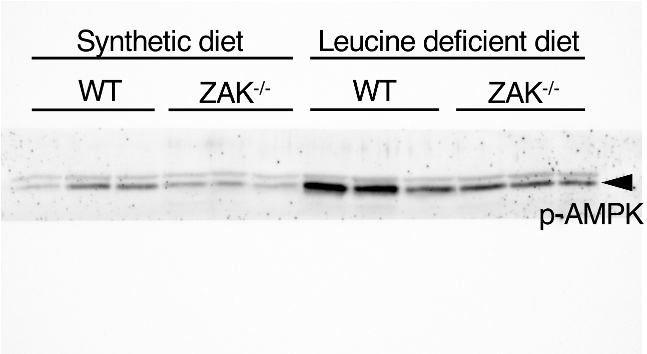
Synthetic diet
Leucine deficient diet
WT
ZAK-/-
WT
ZAK-/-
p-AMPK

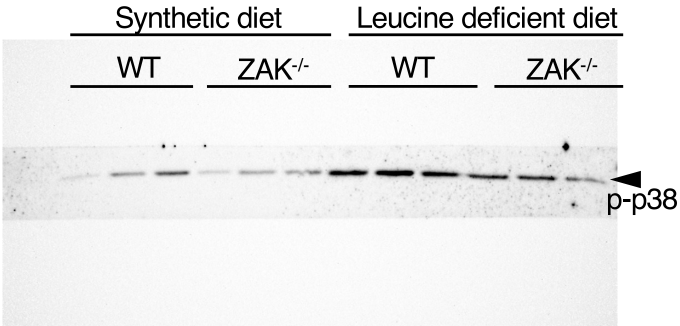
Synthetic diet
Leucine deficient diet
WT
ZAK-/-
WT
ZAK-/-
p-p38

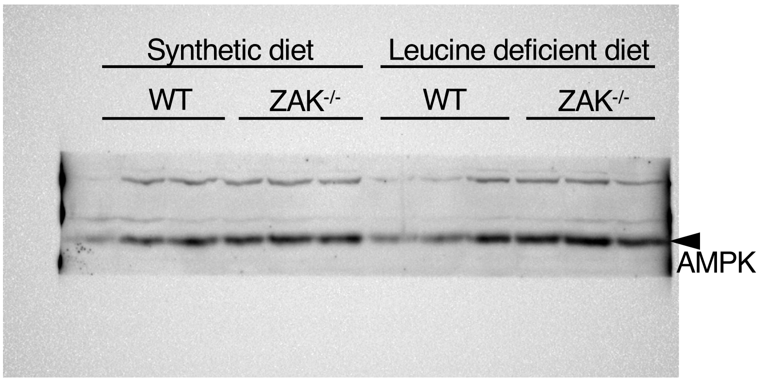
Synthetic diet
Leucine deficient diet
WT
ZAK-/-
WT
ZAK-/-
AMPK

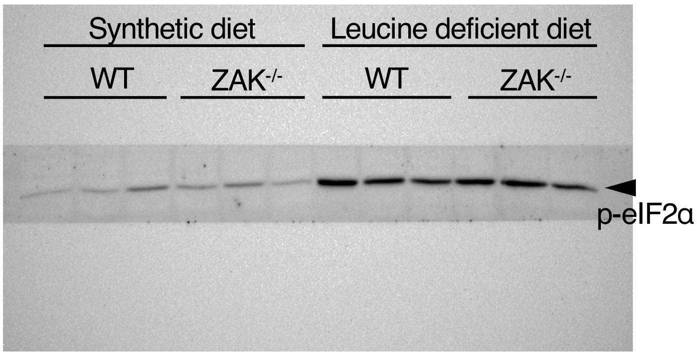
Synthetic diet
Leucine deficient diet
WT
ZAK-/-
WT
ZAK-/-
p-eIF2α

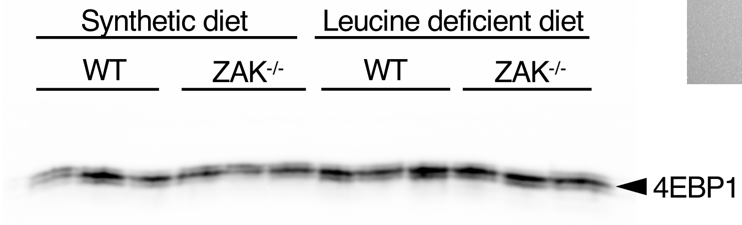
Synthetic diet
Leucine deficient diet
WT
ZAK-/-
WT
ZAK-/-
4EBP1

Figure 3d

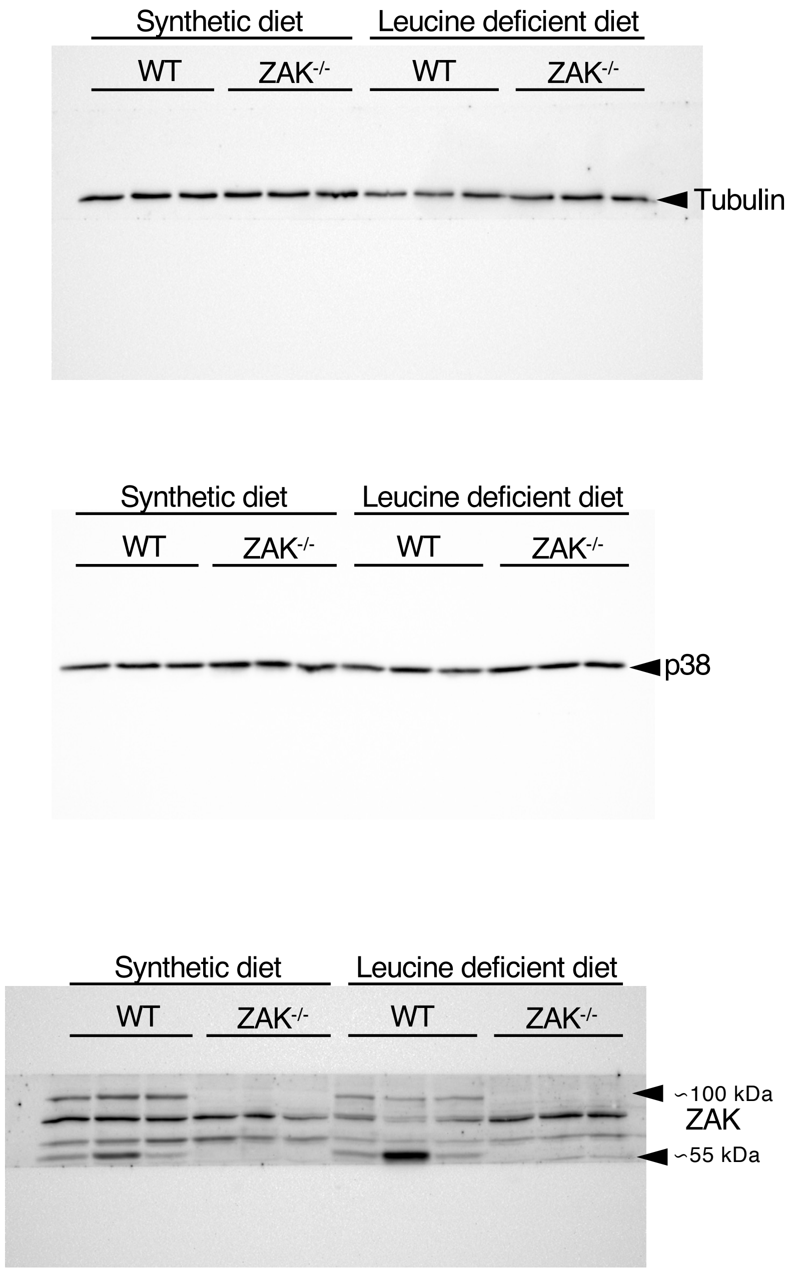
Synthetic diet
Leucine deficient diet
WT
ZAK-/-
WT
ZAK-/-
Tubulin
Synthetic diet
Leucine deficient diet
WT
ZAK-/-
WT
ZAK-/-
p38
Synthetic diet
Leucine deficient diet
WT
ZAK-/-
WT
ZAK-/-
~100 kDa
ZAK
~55 kDa

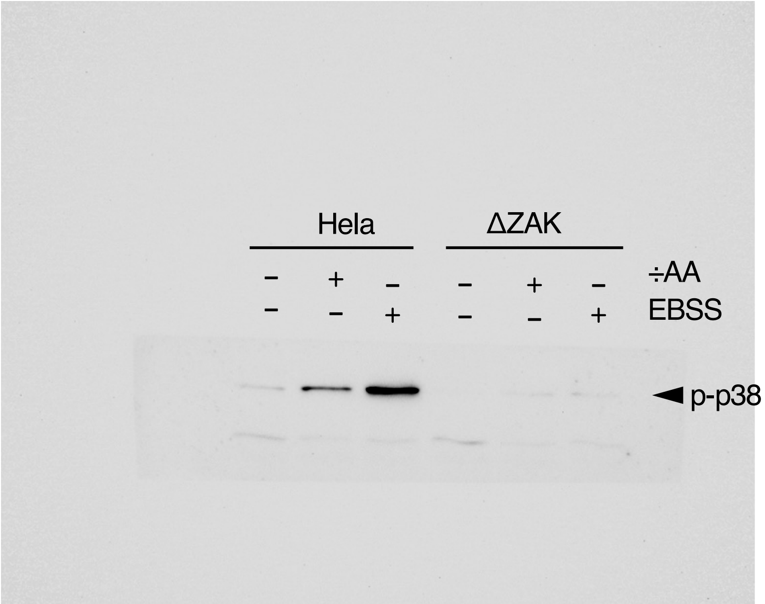

Figure S1a

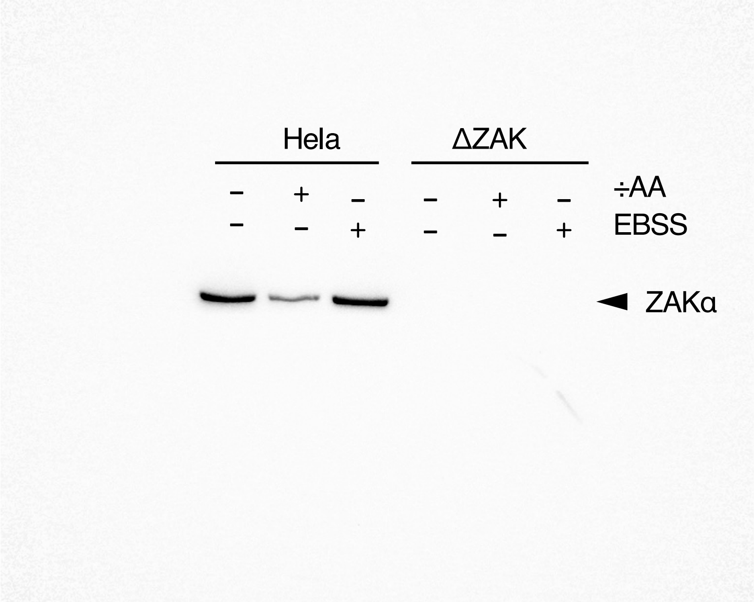

Figure S1b

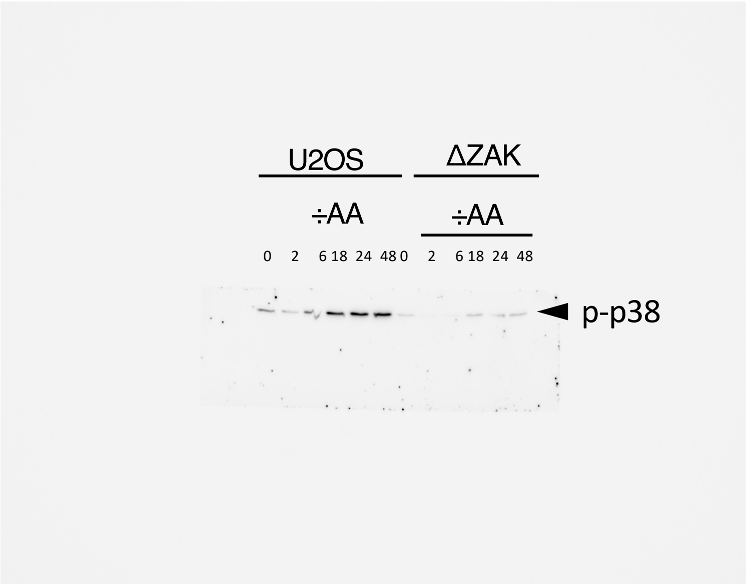
U2OS
ΔZAK
÷AA
÷AA
0 2 6 18 24 48 0 2 6 18 24 48
p-p38

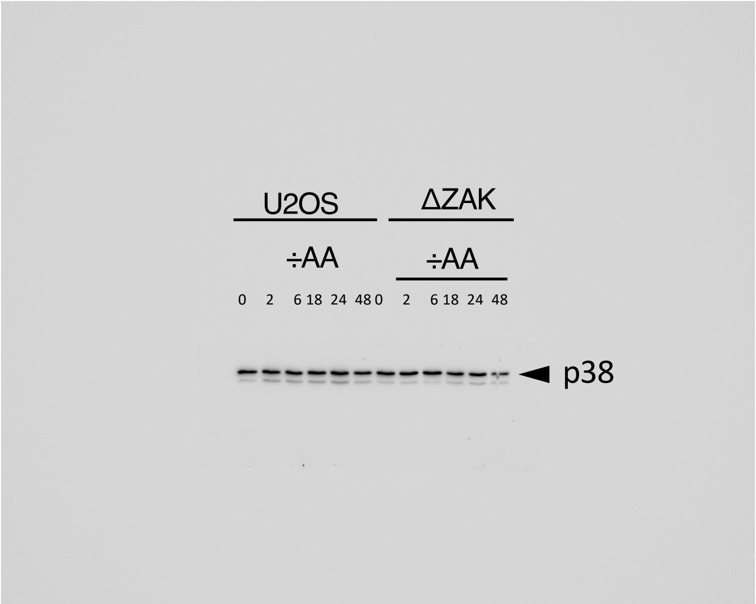
U2OS
ΔZAK
÷AA
÷AA
0 2 6 18 24 48 0 2 6 18 24 48
p38

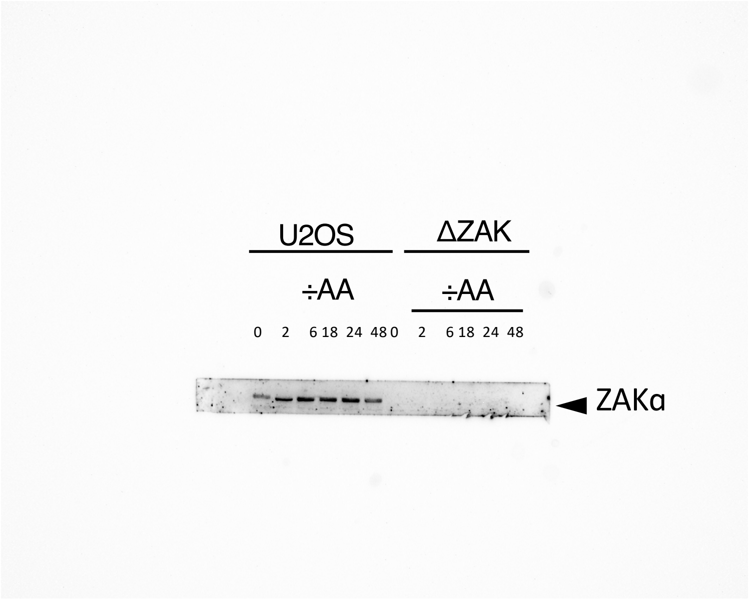
U2OS
ΔZAK
÷AA
÷AA
0 2 6 18 24 48 0 2 6 18 24 48
ZAKα

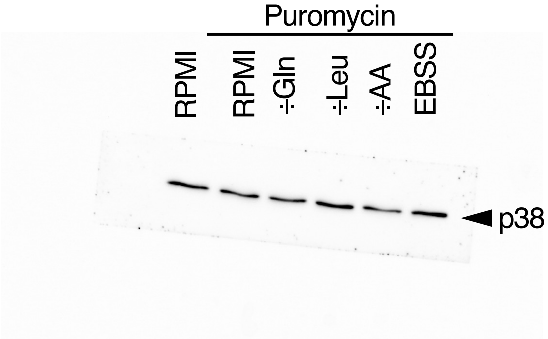
Puromycin
RPMI
RPMI
÷Gln
÷Leu
÷AA
EBSS
p38

Figure S1c

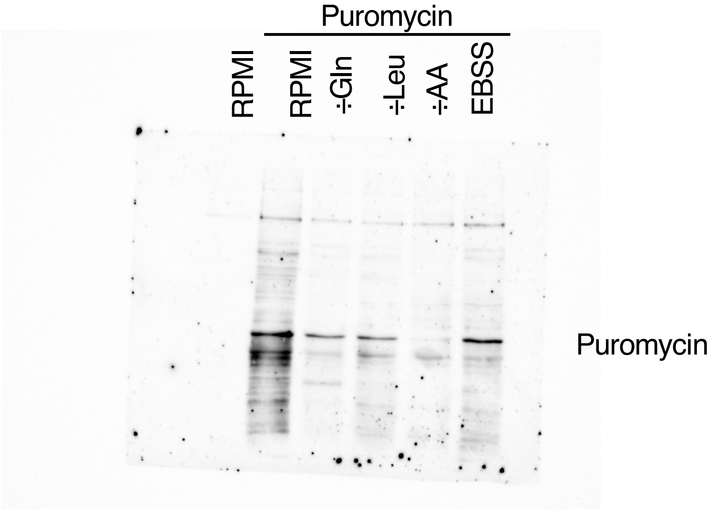
Puromycin
RPMI
RPMI
÷Gln
÷Leu
÷AA
EBSS
Puromycin

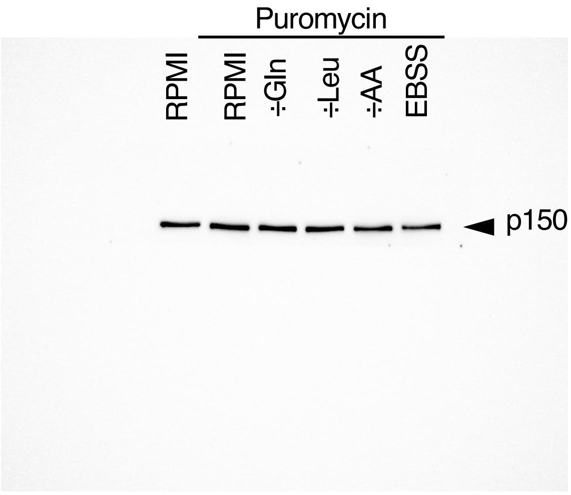
Puromycin
RPMI
RPMI
÷Gln
÷Leu
÷AA
EBSS
p150

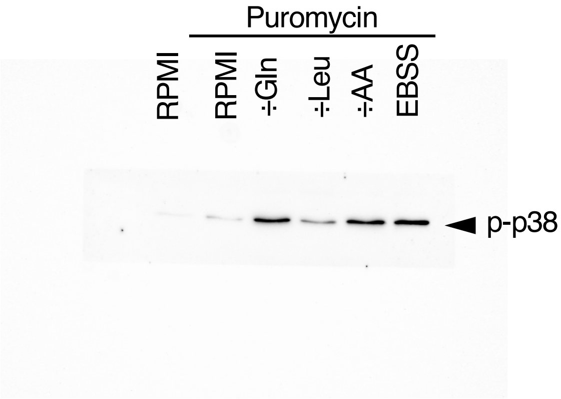
Puromycin
RPMI
RPMI
÷Gln
÷Leu
÷AA
EBSS
p-p38

Figure S1 d

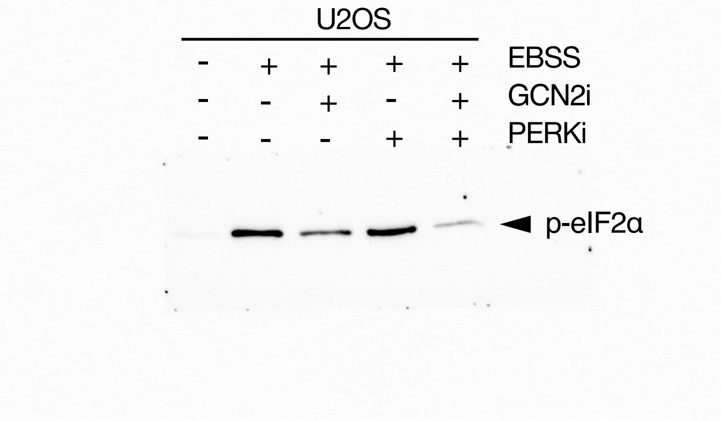

U2OS
EBSS
GCN2i
PERKi
p-eIF2α

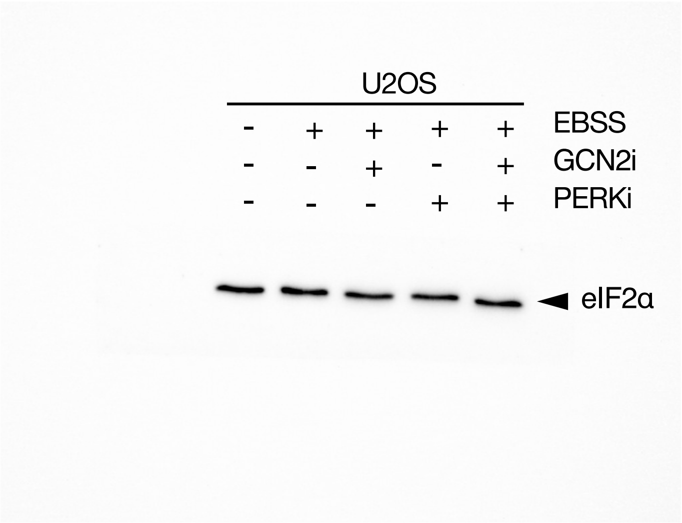

U2OS
EBSS
GCN2i
PERKi
eIF2α

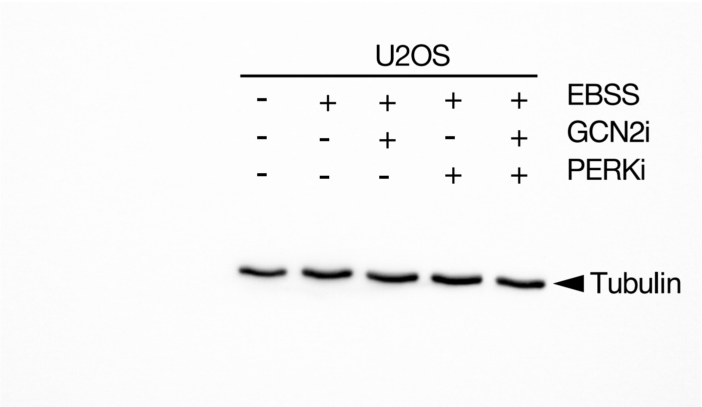

U2OS
EBSS
GCN2i
PERKi
Tubulin

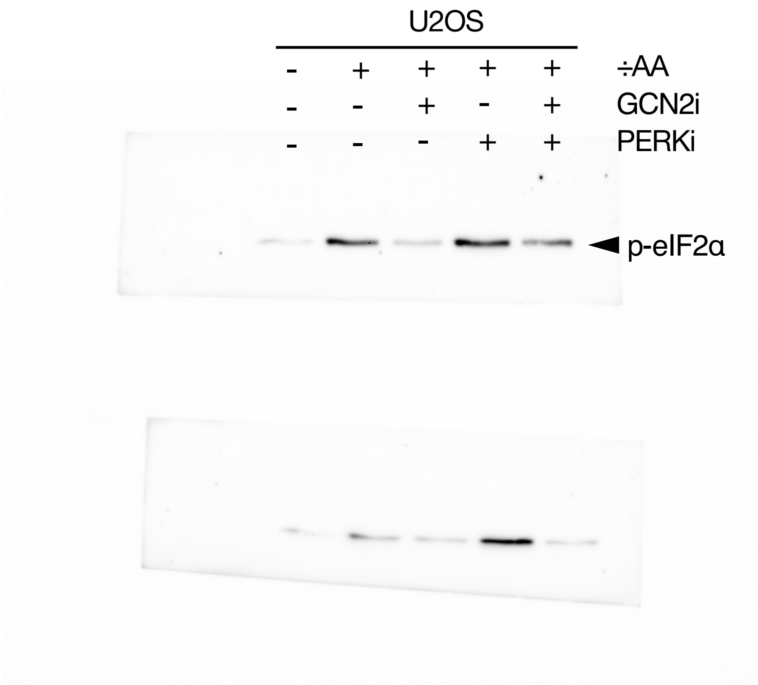
U2OS
- + + + + ÷AA
- - + - + GCN2i
- - - + + PERKi
p-eIF2α

Figure S1e

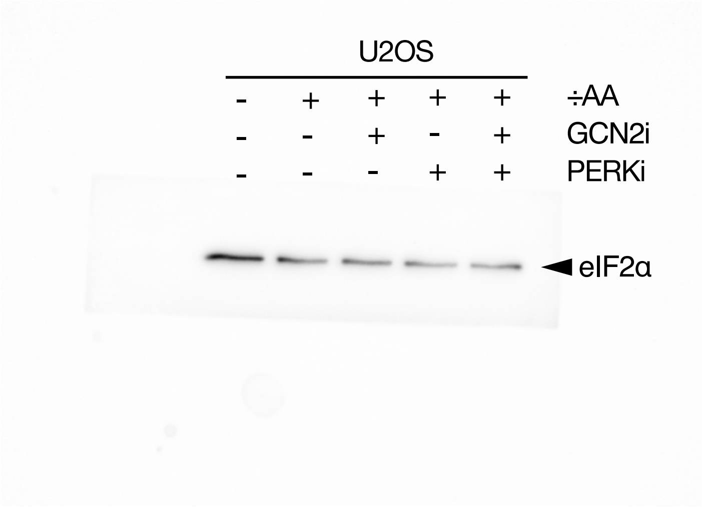
U2OS
- + + + + ÷AA
- - + - + GCN2i
- - - + + PERKi
eIF2α

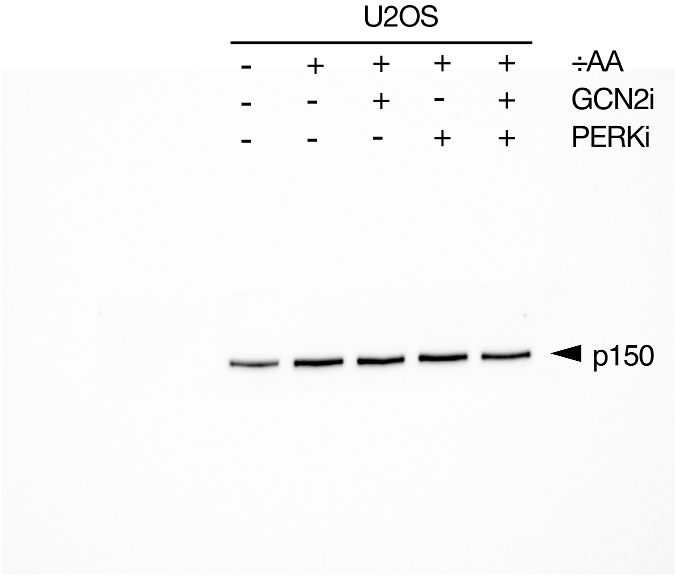
U2OS
- + + + + ÷AA
- - + - + GCN2i
- - - + + PERKi
p150

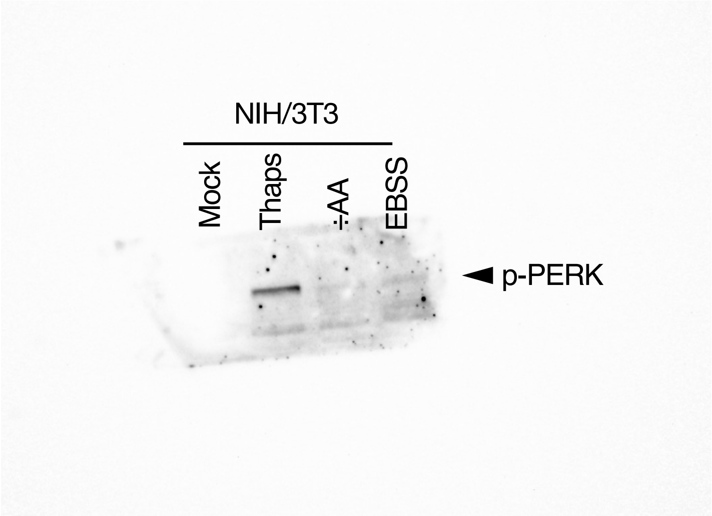
NIH/3T3
Mock
Thaps
÷AA
EBSS
p-PERK

Figure S1f

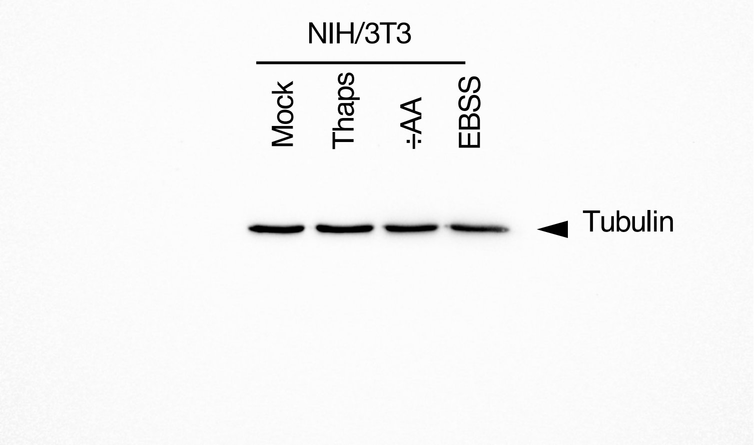
NIH/3T3
Mock
Thaps
÷AA
EBSS
Tubulin

Figure S1h

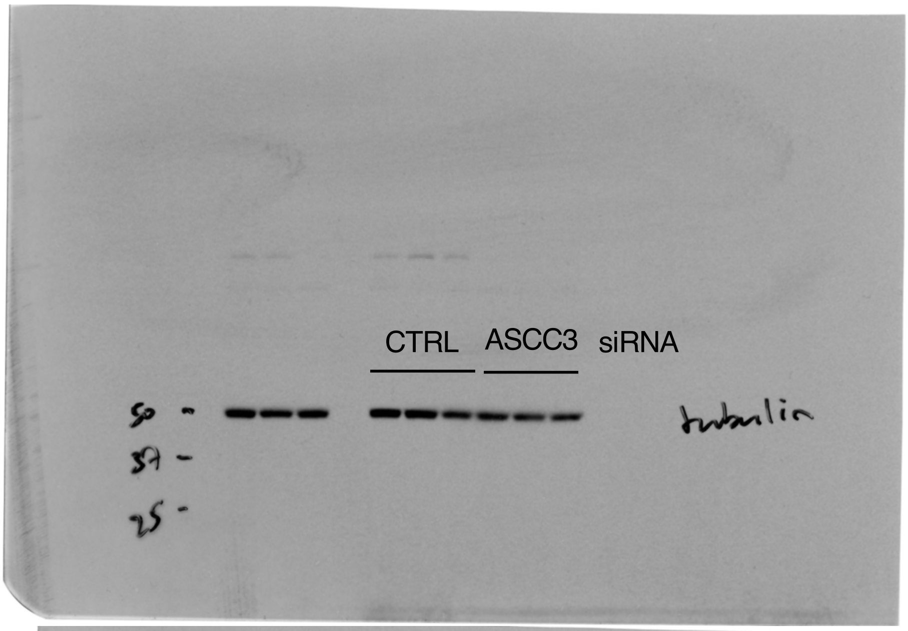
CTRL
ASCC3
siRNA
50
37
25
tubulin

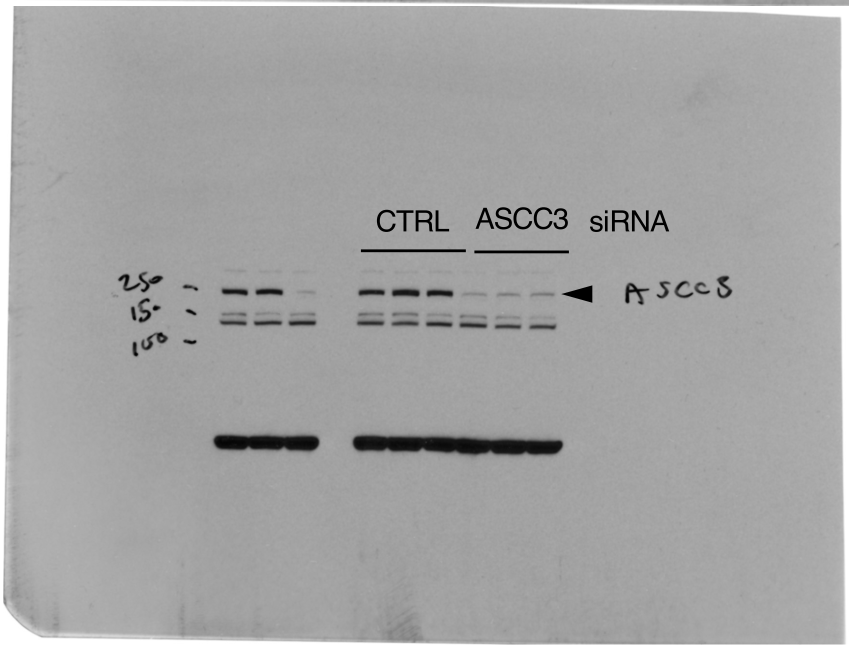
CTRL
ASCC3
siRNA
250
150
100
ASCC3

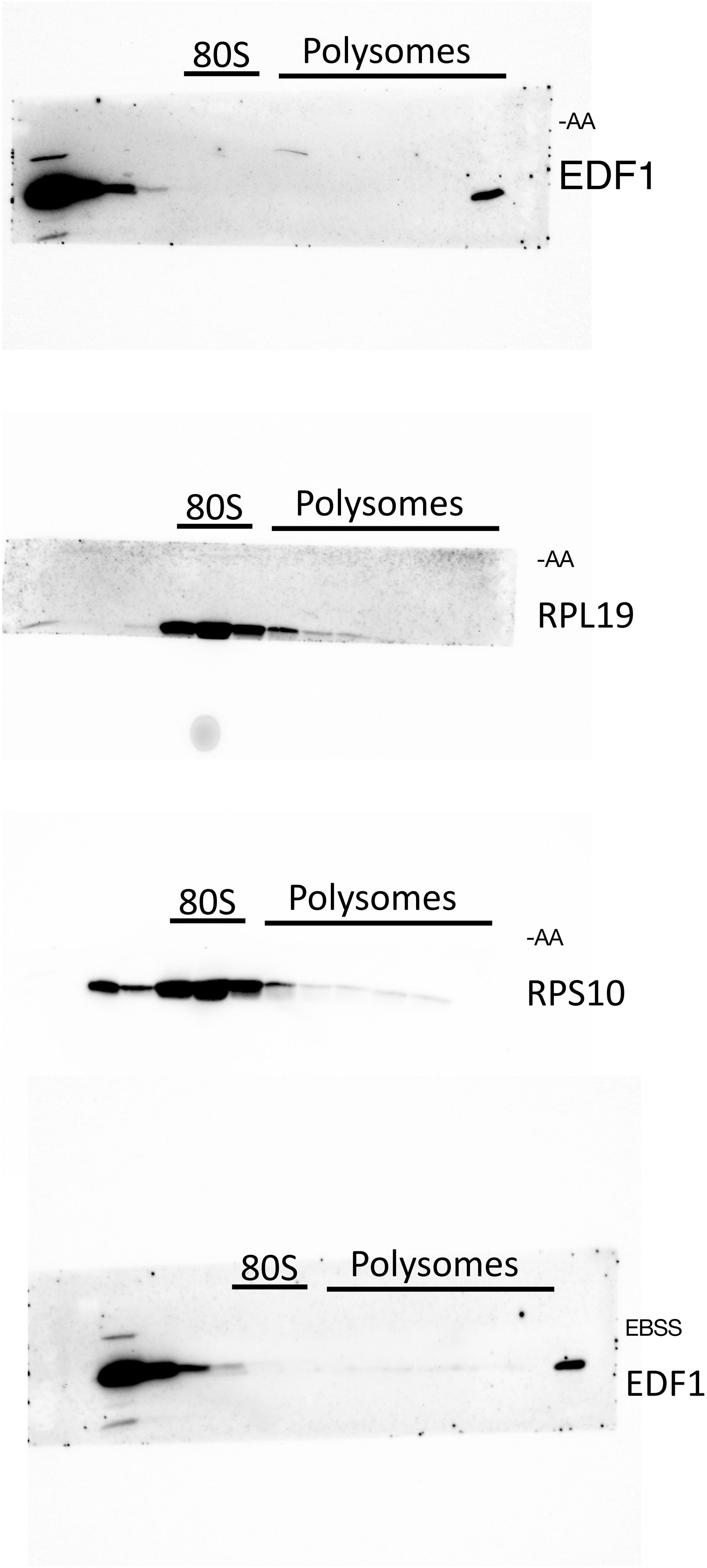
80S
Polysomes
-AA
EDF1
80S
Polysomes
-AA
RPL19
80S
Polysomes
-AA
RPS10
80S
Polysomes
EBSS
EDF1

Figure S1j

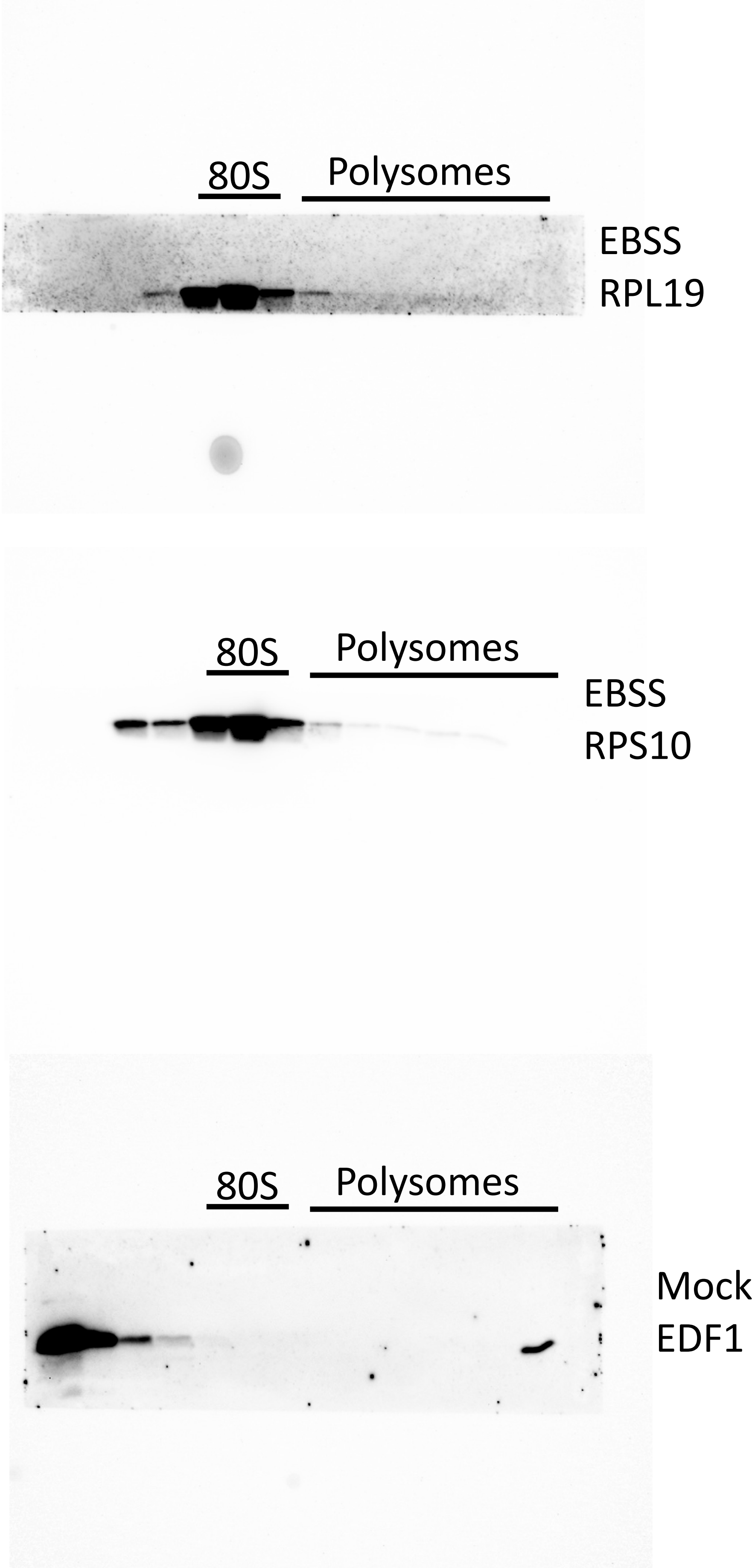
80S
Polysomes
EBSS
RPL19
80S
Polysomes
EBSS
RPS10
80S
Polysomes
Mock
EDF1

Figure S1j

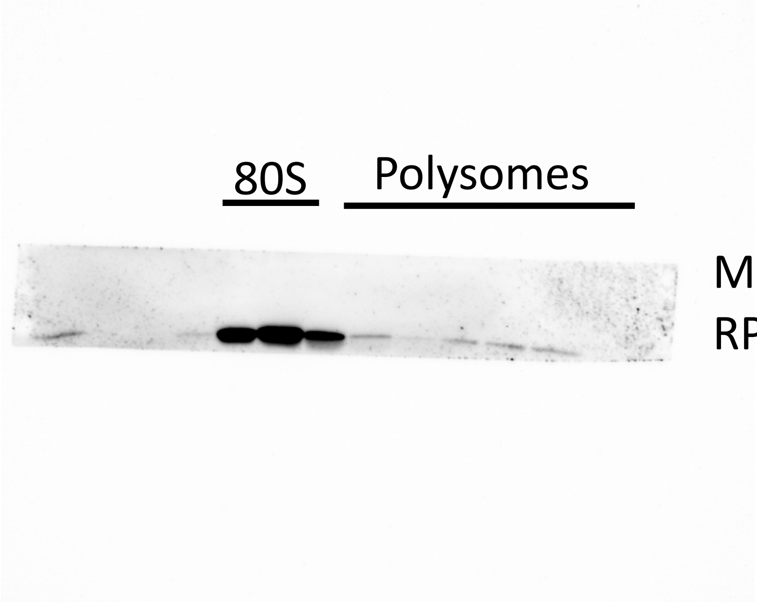

Mock
RPL19

Figure S1j

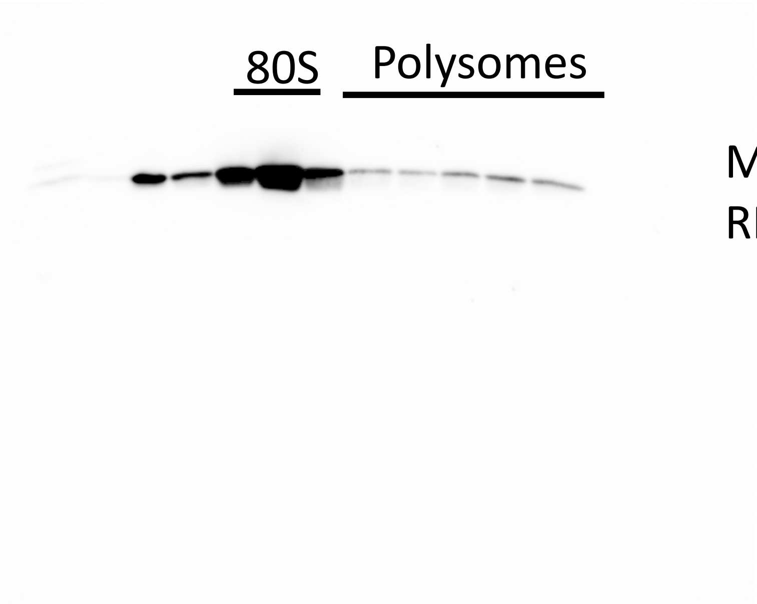

Mock
RPS10

Figure S2a

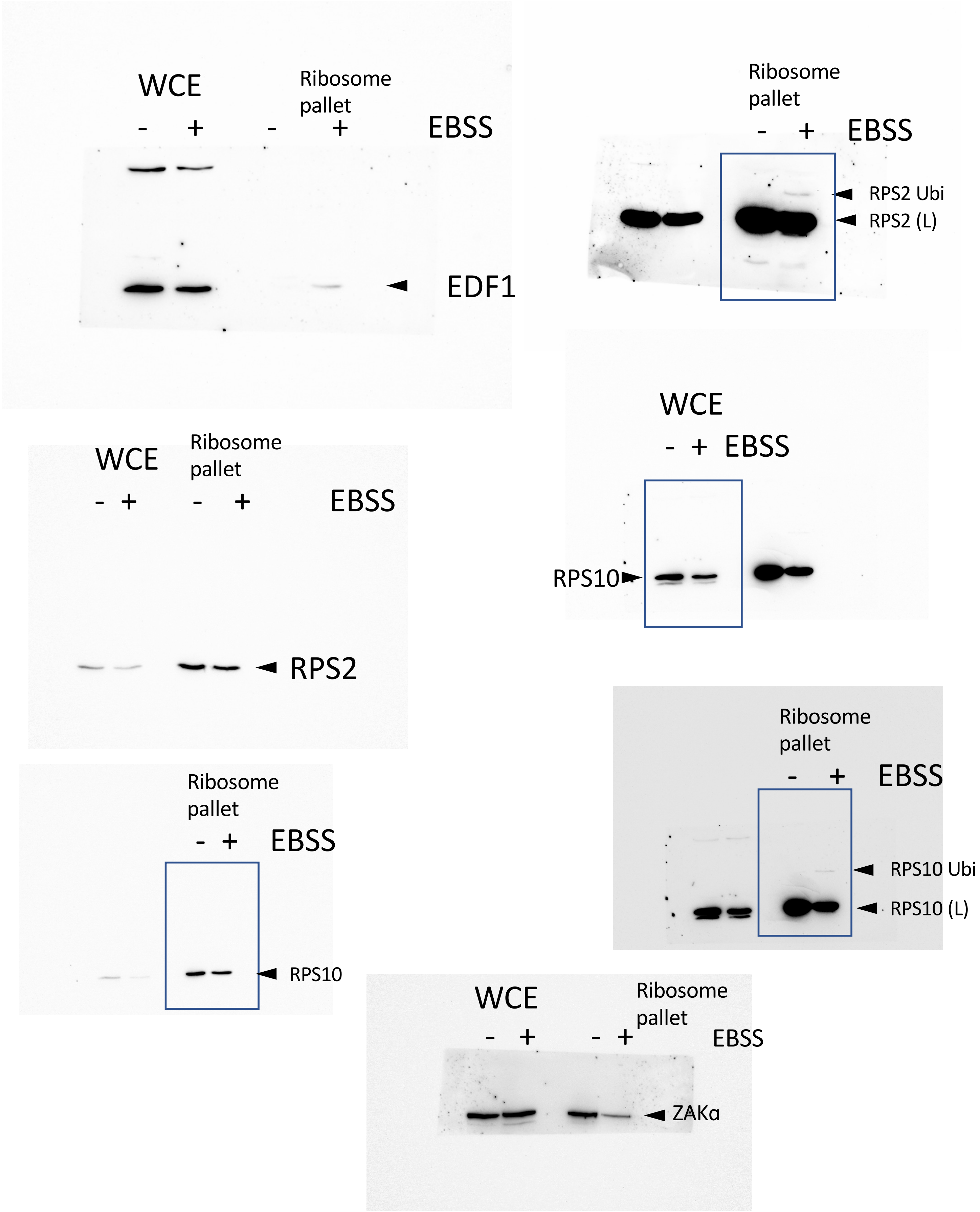
WCE
Ribosome pallet
- + - +
EBSS
EDF1
Ribosome pallet
- +
EBSS
RPS2 Ubi
RPS2 (L)
WCE
- +
EBSS
RPS10
WCE
Ribosome pallet
- + - +
EBSS
RPS2
Ribosome pallet
- +
EBSS
RPS10 Ubi
RPS10 (L)
Ribosome pallet
- +
EBSS
RPS10
WCE
Ribosome pallet
- + - +
EBSS
ZAKα

Figure S2b

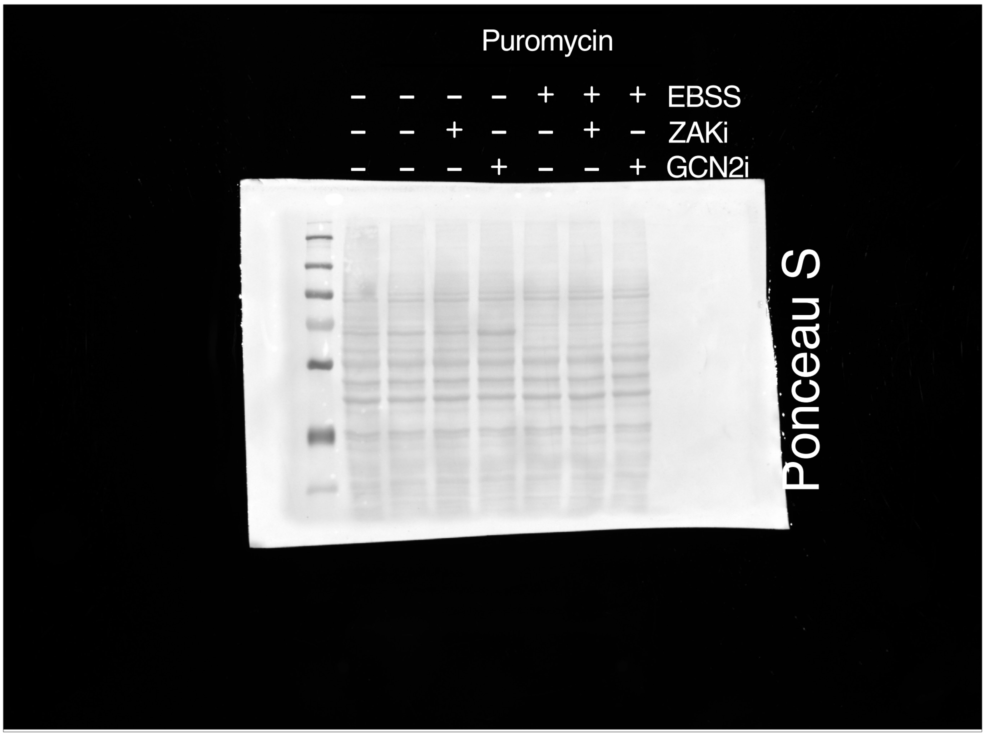

Puromycin
– – – – + + + EBSS
– – + – – + – ZAKi
– – – + – – + GCN2i
Ponceau S

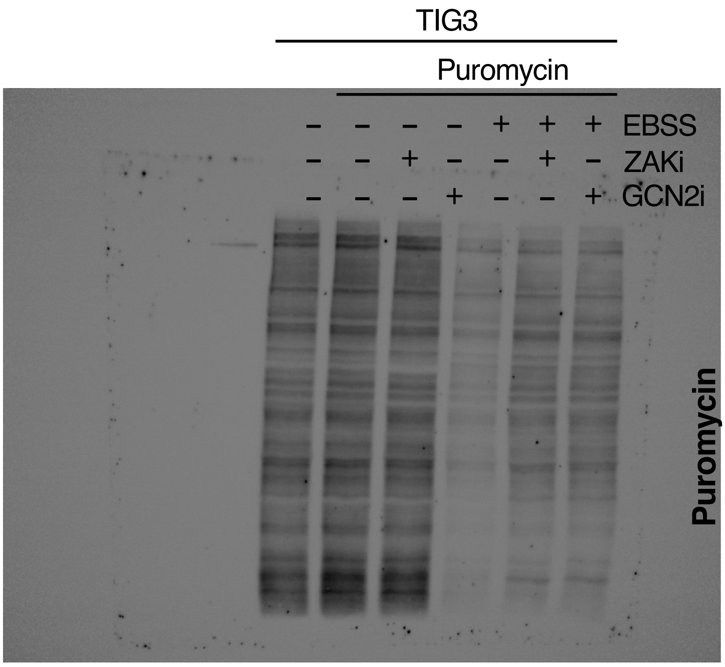

TIG3
Puromycin
– – – – + + + EBSS
– – + – – + – ZAKi
– – – + – – + GCN2i
Puromycin

Figure S2e

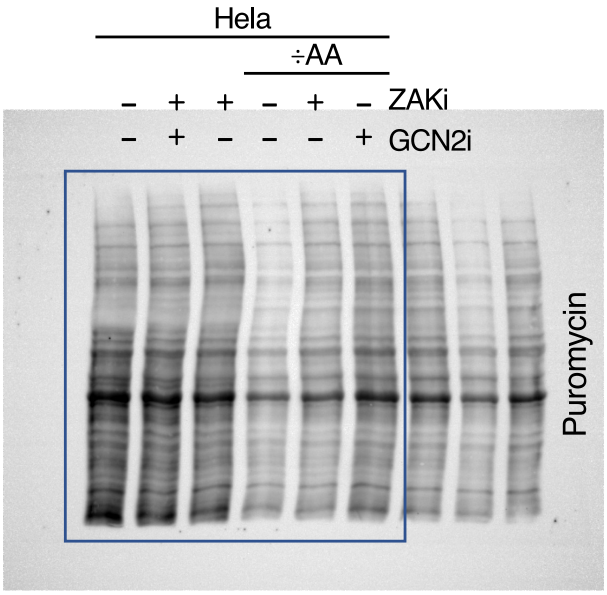

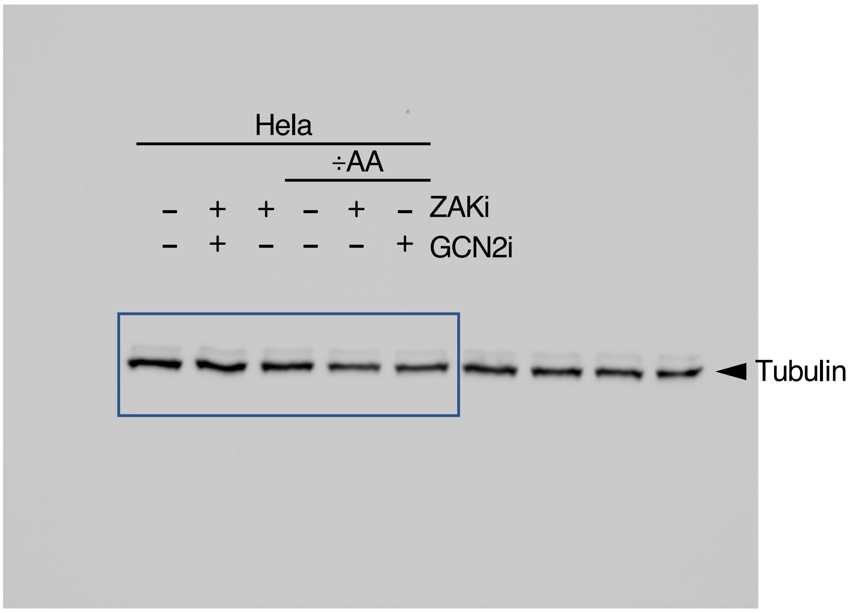

Figure S2g

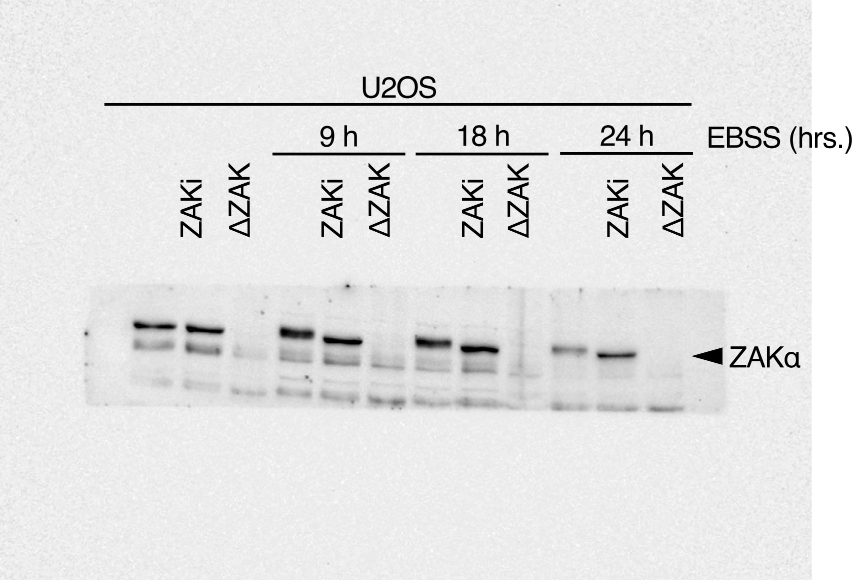
U2OS
9 h
18 h
24 h
EBSS (hrs.)
ZAKi
ΔZAK
ZAKi
ΔZAK
ZAKi
ΔZAK
ZAKi
ΔZAK
ZAKα

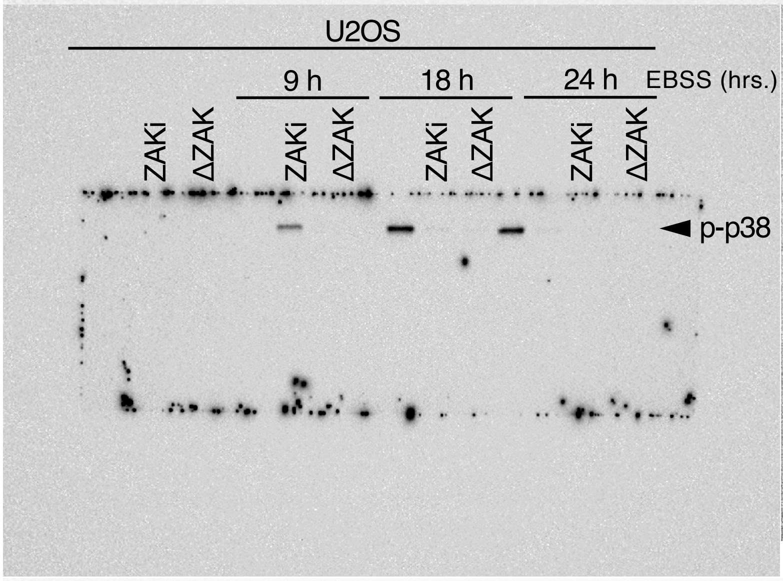
U2OS
9 h
18 h
24 h
EBSS (hrs.)
ZAKi
ΔZAK
ZAKi
ΔZAK
ZAKi
ΔZAK
ZAKi
ΔZAK
p-p38

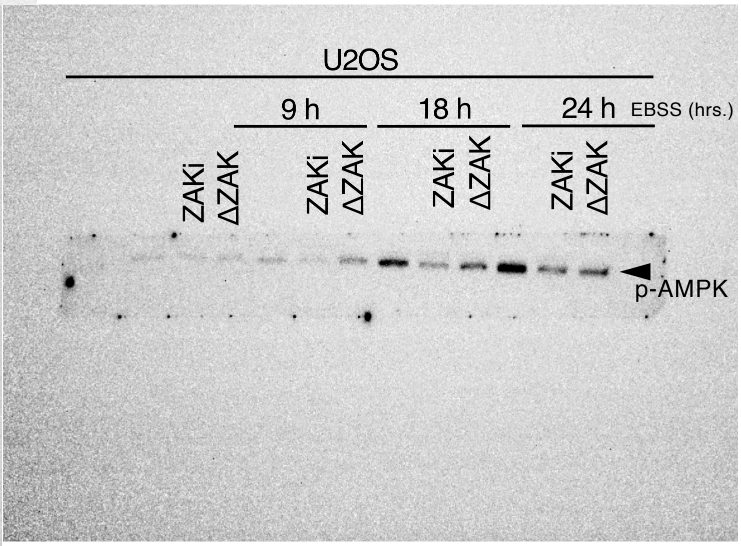
U2OS
9 h
18 h
24 h
EBSS (hrs.)
ZAKi
ΔZAK
ZAKi
ΔZAK
ZAKi
ΔZAK
ZAKi
ΔZAK
p-AMPK

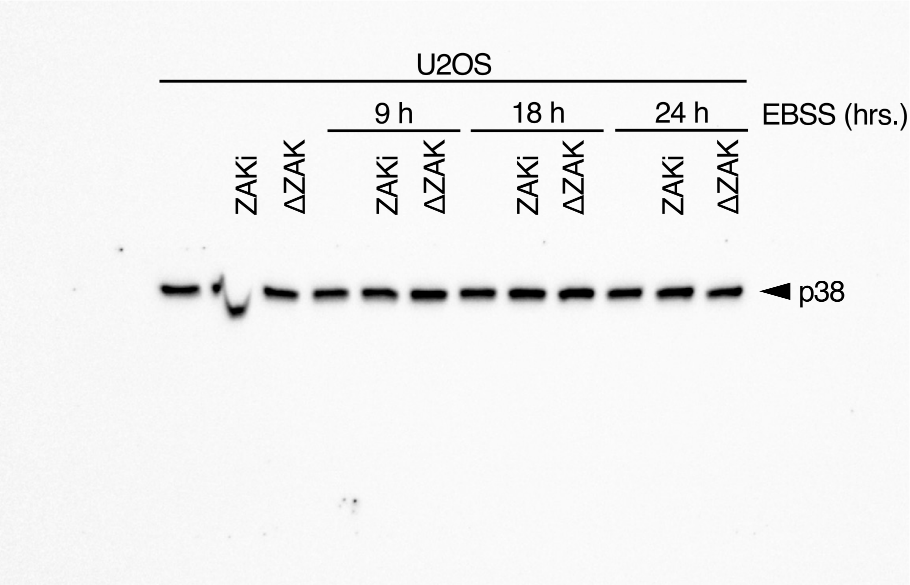
U2OS
9 h
18 h
24 h
EBSS (hrs.)
ZAKi
ΔZAK
ZAKi
ΔZAK
ZAKi
ΔZAK
ZAKi
ΔZAK
p38

Figure S2h

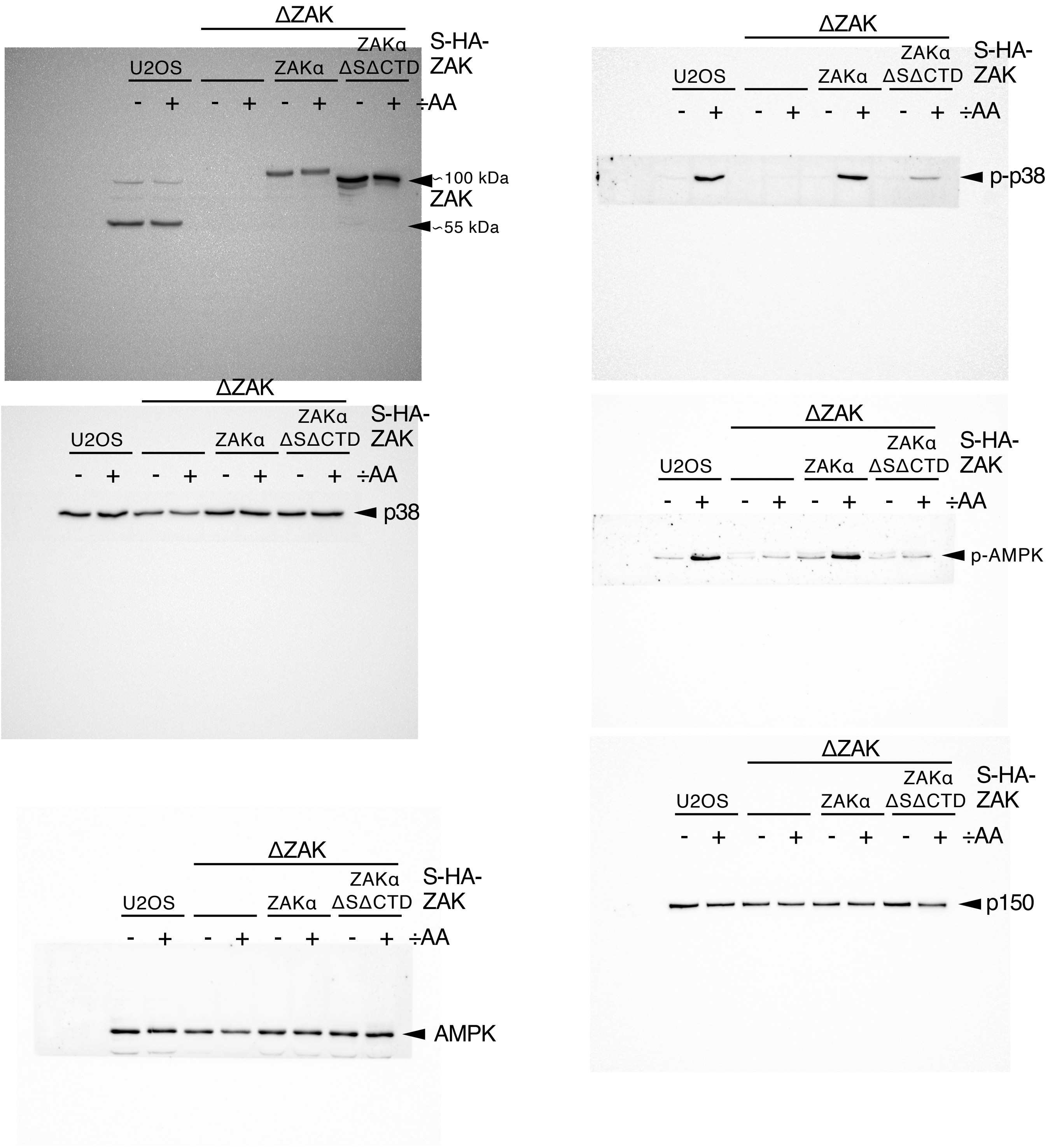
ΔZAK
U2OS
ZAKα
ZAKα ΔSΔCTD
S-HA-ZAK
- + - + - + - + ÷AA
~100 kDa
ZAK
~55 kDa
p-p38
p38
p-AMPK
AMPK
p150

Figure S2j

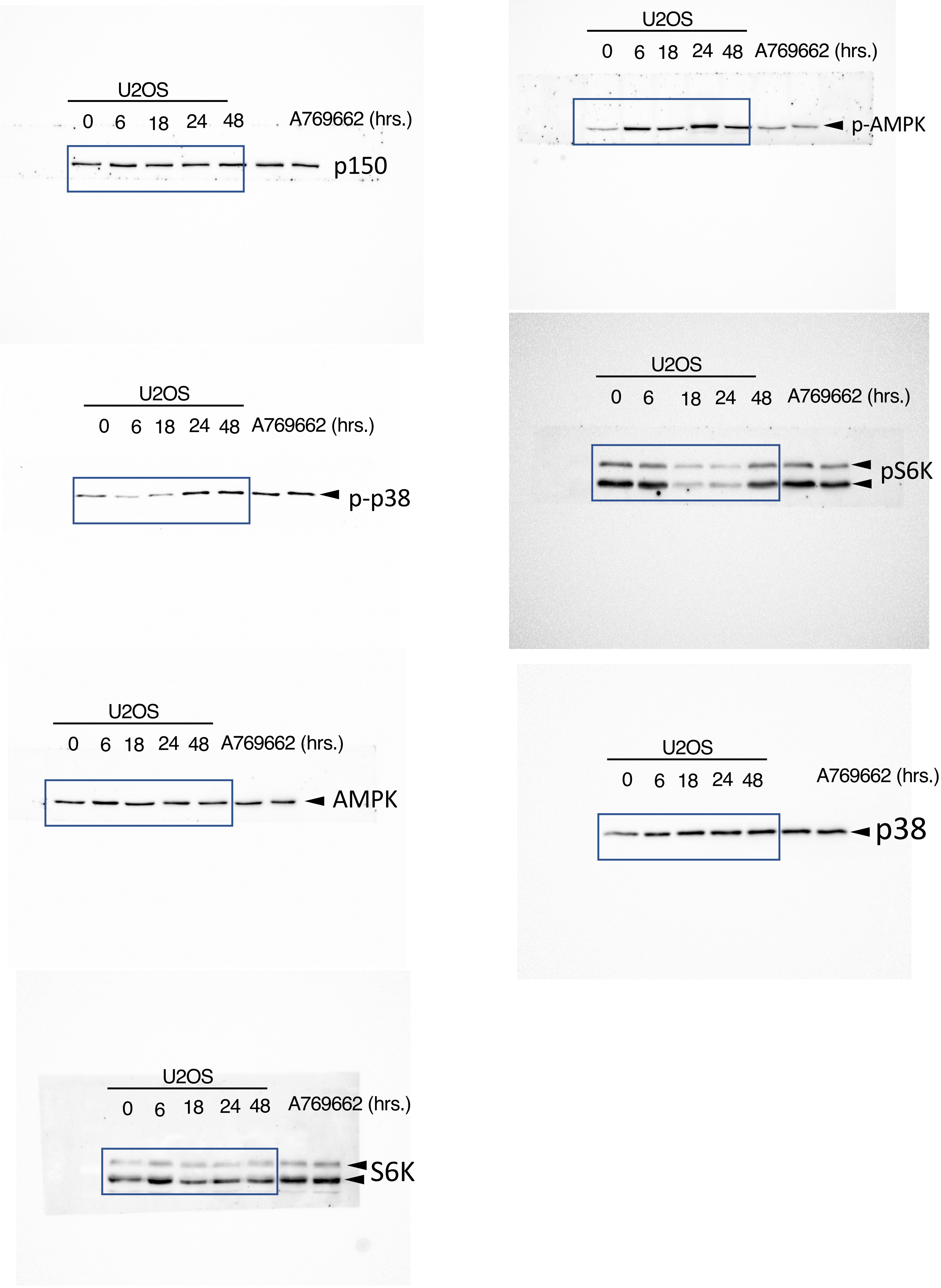
U2OS
0 6 18 24 48 A769662 (hrs.)
p150
U2OS
0 6 18 24 48 A769662 (hrs.)
p-AMPK
U2OS
0 6 18 24 48 A769662 (hrs.)
p-p38
U2OS
0 6 18 24 48 A769662 (hrs.)
pS6K
U2OS
0 6 18 24 48 A769662 (hrs.)
AMPK
U2OS
0 6 18 24 48 A769662 (hrs.)
p38
U2OS
0 6 18 24 48 A769662 (hrs.)
S6K

Figure S3i

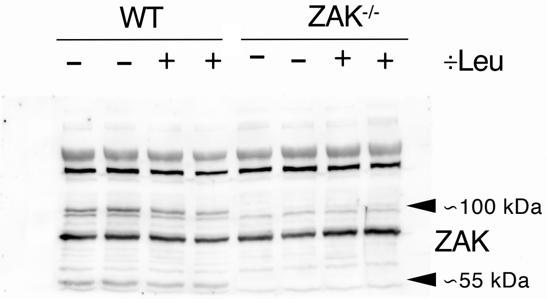

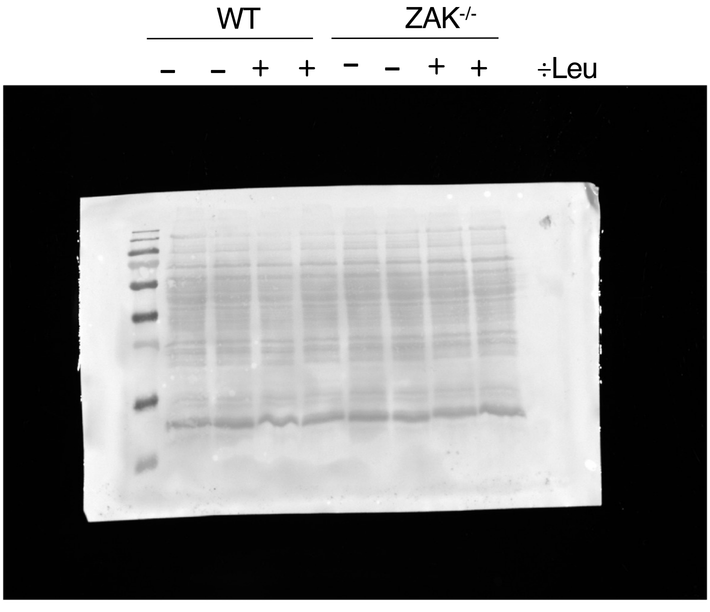

Ponceau S

Figure S3j

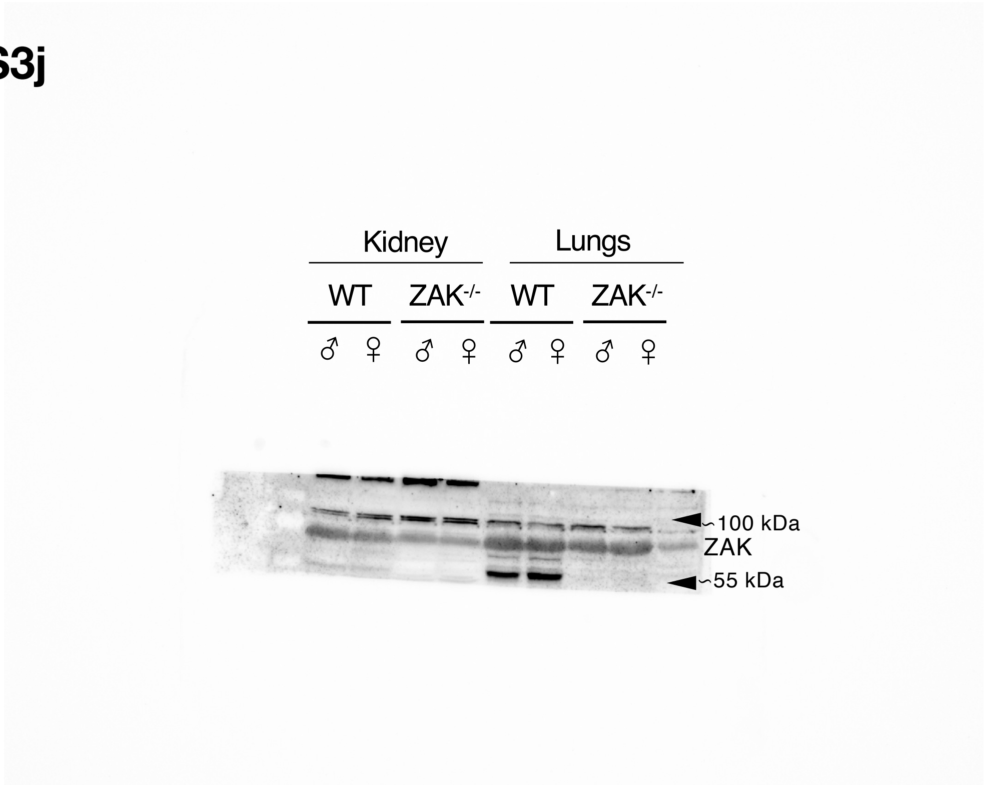
Kidney
Lungs
WT
ZAK-/-
WT
ZAK-/-
♂ ♀ ♂ ♀ ♂ ♀ ♂ ♀
~100 kDa
ZAK
~55 kDa

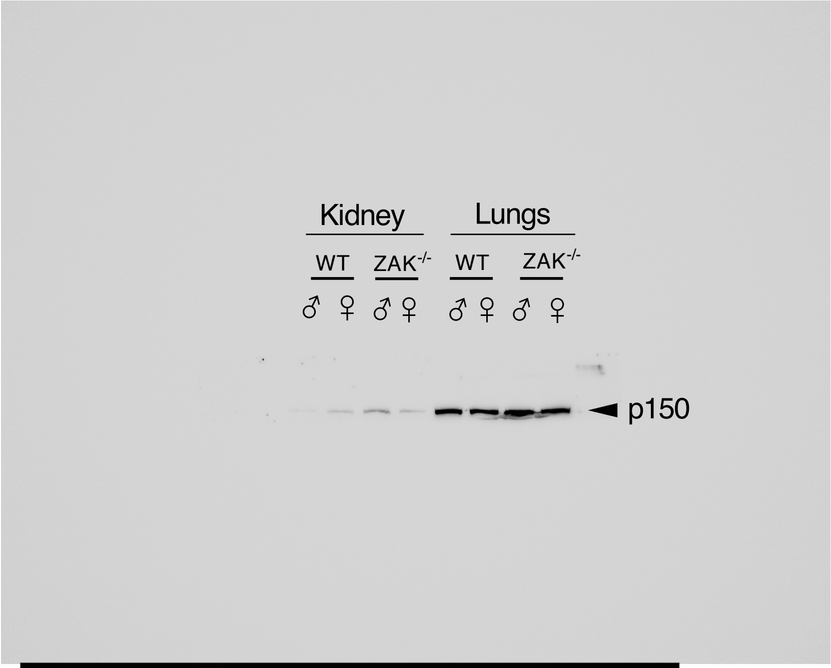
Kidney
Lungs
WT
ZAK-/-
WT
ZAK-/-
♂ ♀ ♂ ♀ ♂ ♀ ♂ ♀
p150

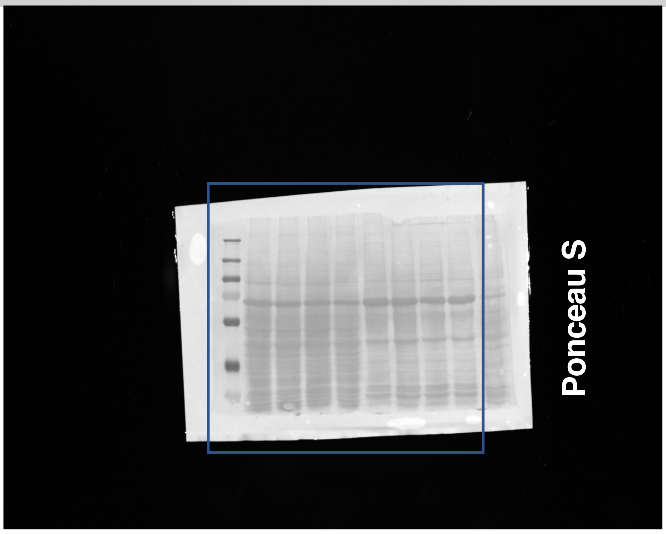
Ponceau S

Figure S3k

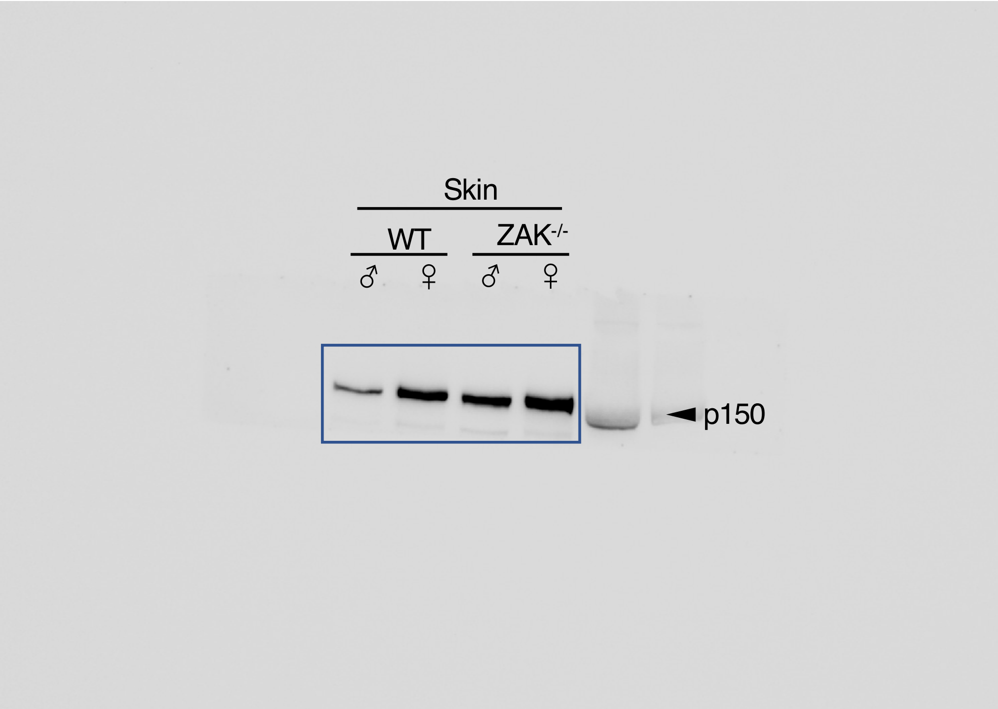

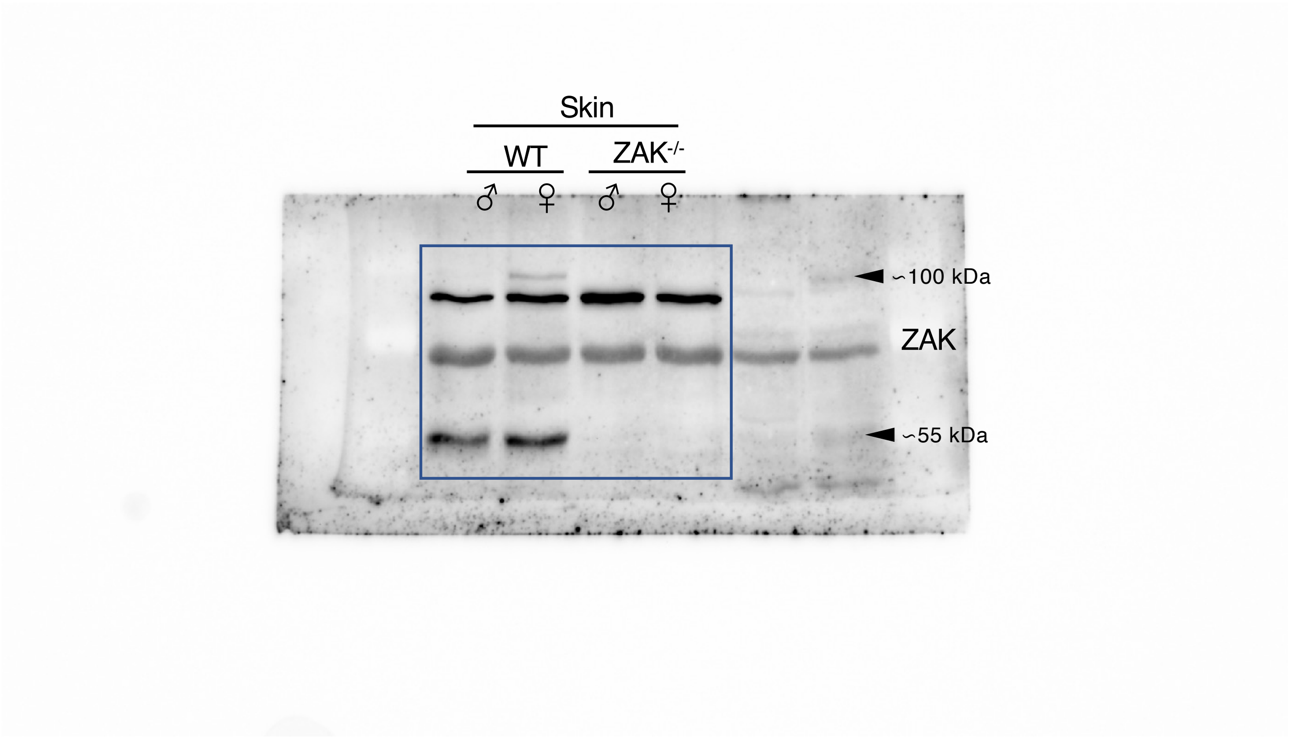

Figure S3I

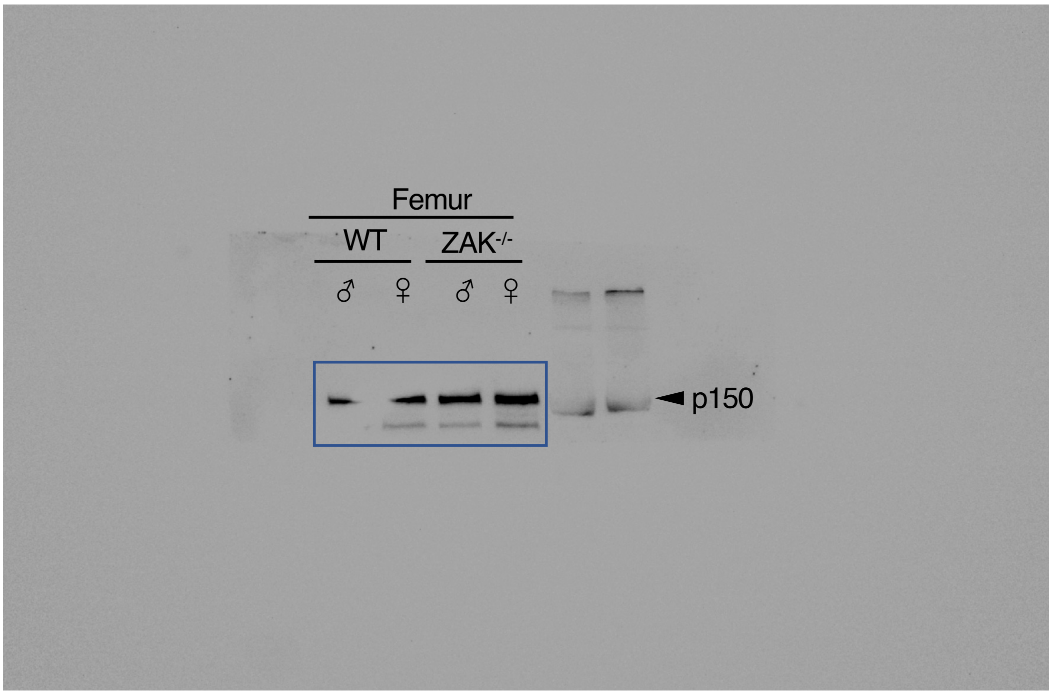

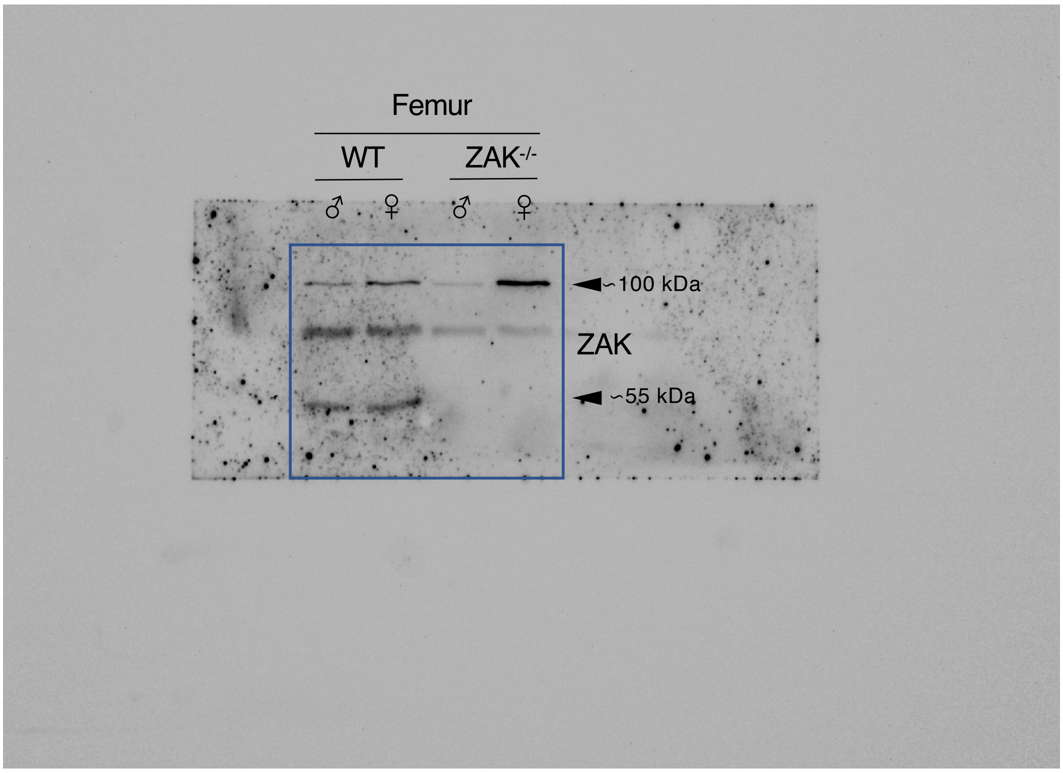

Supplement: Data S1. Source data — 1: A PDF file containing uncropped hi-resolution scans of all the western blots presented in the paper, including those in the supplemental figures. 2: an Excel file containing the values that were used to create all graphs in the paper. [file mmc2.zip › Data S1/WB files.pdf]
